# Impact of onset age of type 2 diabetes mellitus on risk of renal complications compared to age‐matched non‐diabetic patients: Two cohort studies in the United Kingdom and Hong Kong

**DOI:** 10.1111/dom.70061

**Published:** 2025-09-01

**Authors:** Boyuan Wang, Kiki Sze Nga Liu, Ivy Lynn Mak, Edmond Pui Hang Choi, Cindy Lo Kuen Lam, Eric Yuk Fai Wan

**Affiliations:** ^1^ Department of Family Medicine and Primary Care, Li Ka Shing Faculty of Medicine The University of Hong Kong, Hong Kong Special Administrative Region China; ^2^ School of Nursing, Li Ka Shing Faculty of Medicine The University of Hong Kong Hong Kong Special Administrative Region China; ^3^ Department of Family Medicine The University of Hong Kong‐Shenzhen Hospital Shenzhen China; ^4^ Centre for Safe Medication Practice and Research, Department of Pharmacology and Pharmacy, Li Ka Shing Faculty of Medicine The University of Hong Kong Hong Kong Special Administrative Region China; ^5^ Advanced Data Analytics for Medical Science Limited Hong Kong Special Administrative Region China; ^6^ The Institute of Cardiovascular Science and Medicine, Li Ka Shing Faculty of Medicine The University of Hong Kong Hong Kong Special Administrative Region China

**Keywords:** cohort study, diabetes complications, primary care, type 2 diabetes

## Abstract

**Objective:**

With the increasing incidence of early‐onset type 2 diabetes mellitus (T2DM) and the significant burden of renal complications in younger patients, this study aimed to investigate the association between the age at onset of T2DM and the risks of renal complications.

**Methods:**

Two retrospective cohort studies were conducted among adults without chronic kidney disease (CKD) from 2008 to 2013, utilising electronic health records from the United Kingdom (UK) and Hong Kong (HK). A total of 124 610 and 183 114 patients newly diagnosed with T2DM were included in the T2DM group, and 4 968 819 and 3 290 862 individuals without diabetes were included in the comparison group for the UK and HK cohorts, respectively. Participants were categorised into six age groups at baseline (18–39, 40–49, 50–59, 60–69, 70–79, and ≥ 80 years). Propensity score fine stratification weights were applied within each age group, and Cox regression analyses were performed to evaluate the association between T2DM and the risks of renal complications, including CKD, renal decline, and end‐stage renal disease (ESRD).

**Results:**

Over a median followup of 11.6 years (approximately 57 million person‐years) in the UK cohort and 9.7 years (approximately 35 million person‐years) in the HK cohort, T2DM was associated with an increased risk of CKD, renal decline, and ESRD. However, the risks decreased with increasing age at T2DM onset. In the UK cohort, the hazard ratios (HRs) [95% confidence intervals (CIs)] for the three outcomes among individuals with T2DM onset at 18–39 years versus those without diabetes were 1.87 (1.65, 2.11), 4.87 (4.38, 5.40), and 4.49 (3.03, 6.68), respectively. For individuals with T2DM onset at ≥80 years, the HRs decreased to 1.26 (1.21, 1.32), 1.66 (1.55, 1.78), and 1.48 (1.12, 1.96), respectively. A similar trend was observed in the HK cohort, with risks for all outcomes decreasing as the age of T2DM onset increased.

**Conclusion:**

Early‐onset T2DM is associated with substantially higher risks of CKD, renal decline, and ESRD, underscoring the need for targeted prevention and management strategies to reduce renal complications in younger patients with T2DM.

## 1 INTRODUCTION

Diabetes mellitus (DM), being a significant health issue that affects approximately 463 million adults globally in 2019,^1^ is known for its microvascular and macrovascular complications leading to multiorgan dysfunction.^2^ Renal pathology associated with DM is well‐recognised,^3^ contributing to increased risks of chronic kidney disease (CKD) in patients with DM.^4^, ^5^ With a rising incidence of type 2 diabetes mellitus (T2DM) in young people,^6^, ^7^ the increase in microvascular complication rates among individuals with early‐onset T2DM, defined as that diagnosed before 40 years of age,^8^ as compared to late‐onset T2DM,^9^, ^10^ has been gaining global attention. However, limited evidence is available for the differences in the renal risks at various ages of onset by comparing individuals with and without T2DM.

A study investigating the differences in risks of renal complications between early‐onset and late‐onset T2DM has demonstrated a higher prevalence of end‐stage renal disease (ESRD) in early‐onset than late‐onset DM among patients with both T2DM and CKD.^11^ Another study revealed a lower incidence of ESRD in early‐onset T2DM in the initial 15 years after DM onset, followed by a higher incidence beyond 20 years of follow‐up.^12^ A recent study in Hong Kong measured the incidence rate ratios of CKD in patients with T2DM that were diagnosed 5 years apart and compared the ratios at different ages at the diagnosis of T2DM.^13^ Stratification by age at diagnosis allowed a comparison of CKD risks in each 5‐year increase in DM duration at different onset ages, which demonstrated the most significant increase in CKD risks in patients diagnosed at 20–29 years old, and the effect of the 5‐year increase in duration is reduced with increasing age at DM diagnosis, yielding no significant association at or above 70 years old. The results highlighted the amplification effect of T2DM onset age on the positive correlation between T2DM duration and CKD risks, thus suggesting that an earlier age of onset of DM yielded a higher risk of CKD regardless of DM duration. However, confounders such as the presence of other comorbidities, including metabolic syndrome, may affect the progression of both DM and CKD among patients with existing DM in previous studies.^14^, ^15^ Comparison between individuals with and without DM is highly important to identify the actual impact of DM onset across different age groups adjusting for comorbidities. Hence, this study aimed to evaluate the impact of T2DM onset age on the risks of CKD and ESRD through comparing patients with DM against age‐matched individuals without DM in Hong Kong and the United Kingdom.

## 2 METHOD

### 2.1 Study design

This retrospective cohort study targeted patients aged 18 and above who attended health services between January 2008 and December 2013 by adapting the electronic health record database in the United Kingdom (UK) and Hong Kong (HK). For the UK database, records were obtained from the Health Improvement Network (THIN), a database established in 2003 that includes data from over 828 practices, covering more than 12 million individuals. THIN has been validated as being representative of the UK general population.^16^ This database has been widely used and validated in various studies.^17^, ^18^, ^19^, ^20^ In the United Kingdom, 87% of the population is Caucasian.^21^ While the database in HK was provided by the Hospital Authority (HA). The HA is responsible for managing all public hospital services in Hong Kong, including 43 hospitals and institutions, 49 specialist Out‐patient Clinics, and 73 general Out‐patient Clinics.^22^ Records were extracted from the Clinical Management System (CMS) which has been built since 1994.^23^ The validity and coding accuracy of the electronic health records were shown in previous epidemiological studies.^24^, ^25^, ^26^ In HK, Chinese covers 90% of population.^27^

We included all eligible subjects who attended the doctor consultation from January 2008 to December 2013. The new‐onset T2DM group included patients who were either newly diagnosed with T2DM or prescribed anti‐diabetic medications for the first time. In the UK cohort, T2DM diagnosis was extracted by using the Read Codes, which is shown in Supplementary Table 1. For the HK cohort, T2DM was defined by International Classification of Primary Care, Second edition (ICPC‐2) code T90, the International Classification of Diseases, Ninth Edition, Clinical Modification (ICD‐9‐CM) code of 250.x0 and 250.x2. The British National Formulary (BNF) codes of 6.1.1 and 6.1.2 were used to define the prescription of anti‐diabetic drugs for both cohorts. The baseline was then defined by the corresponding date of T2DM diagnosis or prescription of anti‐diabetic drugs. Meanwhile, for the comparison group, subjects who had attended at least one doctor consultation during the inclusion period were considered eligible. The index date was defined as the date of the first doctor consultation during the inclusion period. Subjects with a diagnosis of T2DM, Type 1 DM, or any prescription of anti‐diabetic medications on or before the index date were excluded from the comparison group. Patients diagnosed with CKD before baseline were also excluded from the analysis. CKD is defined using the ICD‐9‐CM code of 585.3–585.6 or 586, the Read Codes summarised in Supplementary Table 1, or by an estimated glomerular filtration rate (eGFR) of <60 mL/min/1.73 m.^2^ The eGFR was calculated based on the CKD‐EPI Creatinine Equation (2021): eGFR = 142 × min (*S*_cr_/*κ*, 1)^α^ × max (*S*_cr_/*κ*, 1)^−1.200^ × 0.9938^Age^ × 1.012 [if female], where eGFR is the eGFR in mL/min/1.73 m^2^; min (*S*_cr_/*κ*, 1) is the minimum of *S*_cr_/*κ* or 1.0; max (*S*_cr_/*κ*, 1) is the maximum of *S*_cr_/κ or 1.0.^28^ All patients were followed until the first date of any outcome event, mortality, transferring out of the practice, 31 December 2018 (HK cohort) or 31 December 2019 (UK cohort), whichever occurred first.

### 2.2 Outcome measures

The primary outcomes were CKD, ESRD, and renal decline after baseline. The ESRD diagnoses were defined by an eGFR of <15 mL/min/1.73 m,^2^ or a diagnosis of ESRD, which was identified by Read Codes in the UK cohort and by ICD‐9‐CM codes 585.5, 585.6, or 586 in the HK cohort. Renal decline was defined as a reduction in eGFR by 30% over 2 years. This threshold has been suggested by previous studies to be strongly predictive of subsequent end‐stage kidney disease and other adverse outcomes.^29^, ^30^, ^31^, ^32^ Other outcomes included all‐cause mortality. In the UK cohort, disease definitions using Read Codes were based on the previous study,^33^, ^34^, ^35^ and are summarised in Supplementary Table 1. Deaths were identified by a valid death date recorded in the THIN dataset. In the HK cohort, data on all‐cause mortality were collected from the Hong Kong Deaths Registry, the governmental registry for the death of Hong Kong residents.

### 2.3 Baseline characteristics

Baseline characteristics included age, sex, smoking status, comorbidities (i.e., obesity, atrial fibrillation, peripheral vascular disease, amputation, dementia, chronic lung disease, connective tissue disease, peptic ulcer disease, liver disease, cardiovascular disease, hemiplegia, leukaemia, malignant lymphoma, cancer, hypertension, retinopathy, and hyperfiltration), and medication use within 1 year prior to baseline, including renin‐angiotensin system agents, beta‐blockers, calcium channel blockers, diuretics, statins, fibrates, and other lipid‐lowering agents. Specifically, obesity was defined by either a diagnosis record or a recorded BMI (i.e., BMI ≥30 kg/m^2^ in the UK cohort and BMI ≥25 kg/m^2^ in the Hong Kong cohort), and hyperfiltration was defined as a recorded eGFR greater than 135 mL/min/1.73 m.^2^ Subjects without an obesity diagnosis and without a recorded BMI above the respective cut‐off values were classified as non‐obese. All other baseline characteristics were defined based on diagnosis or medication prescription records, and there were no missing data for the baseline variables.

### 2.4 Statistical analysis

Analyses were conducted separately for the United Kingdom and Hong Kong cohorts due to differences in coding systems and follow‐up periods. Patients in both the exposed and comparison groups were further divided into six age groups based on the age of T2DM onset or age on the index date, namely (i) 18–39; (ii) 40–49; (iii) 50–59; (iv) 60–69; (v) 70–79 (vi) ≥80 years old, for between‐group comparisons of the risk of outcomes. Within each age group, a fine stratification weighting method based on propensity scores was applied to adjust for confounding. The weights were estimated using the “MMWS” package in Stata. Specifically, within each age group, propensity scores were first estimated as the probability of new‐onset T2DM using a multivariable logistic regression model that included all baseline characteristics described above. Subjects were then stratified into 50 equally sized strata according to their propensity scores. The weight for each subject in age group α was calculated as follows:

$$w_{i,\alpha }=\frac{n_{s,\alpha }\times \mathrm{Pr}\left(Z=z|\alpha \right)}{n_{z=z,s,\alpha }}$$

where $n_{s,\alpha }$ is the total number of individuals in stratum s within age group α, $\mathrm{Pr}\left(Z=z|\alpha \right)$ is the probability of assignment to group z in age group α, and $n_{z=z,s,\alpha }$ is the total number of individuals in stratum s within age group α who were actually assigned to group *z*.^36^, ^37^, ^38^ After weighting, covariate balance in the weighted cohort was assessed using the standardised mean difference (SMD), with an SMD <0.2 considered to indicate sufficient balance between the exposed and comparison groups.^39^

Summary of baseline characteristics was presented in mean and standard deviation for continuous variables or count and proportion for categorical variables. The incidence rates of outcomes by different age groups and the corresponding confidence interval (CI) were estimated based on Poisson distribution. The association between new‐onset T2DM and the risk of outcome events was analysed using multivariable Cox proportional hazard regression adjusted for the patient's baseline characteristics, with the scaled Schoenfeld residuals plotted for each covariate against time to assess the proportional hazard assumptions. To assess multicollinearity among covariates, the variance inflation factor (VIF) was calculated for all variables included in the models. A VIF value of <5 was considered to indicate acceptable collinearity, consistent with commonly used thresholds in epidemiological research.^40^

To ensure the robustness of the results, a total of 15 sensitivity analyses were conducted. The first sensitivity analysis included only subjects with at least one‐year follow‐up period. The second sensitivity analysis applied exact one‐to‐one matching based on age and sex. The third sensitivity analysis excluded subjects in the comparison group who were diagnosed with DM within the first year after baseline. The fourth sensitivity analysis excluded subjects in the comparison group who were diagnosed with DM after baseline. In the fifth sensitivity analysis, one‐to‐one matching based on propensity score rather than fine stratification weights was used. Sixthly, subjects with a diagnosis of DM after baseline were censored in the comparison group. In the seventh sensitivity analysis, the Fine‐Grey subdistribution hazard model was used to evaluate the association between new‐onset DM and the risk of outcomes, with death considered as a competing event.^41^ In the eighth sensitivity analysis, baseline age was treated as a continuous variable. Restricted cubic spline functions with knots at ages 39, 49, 59, 69, and 79 were applied to flexibly model the association between age and renal outcomes. Additionally, interaction terms between DM onset and age (modelled using restricted cubic splines) were included in the model. In the ninth sensitivity analysis, a cut‐off value of 40% was used instead of 30% for the renal decline outcome. In the tenth analysis, subjects without baseline eGFR data were excluded. In the eleventh analysis, renal decline was defined as a 30% decrease in eGFR within 2 years, confirmed by two consecutive eGFR measurements. In the twelfth sensitivity analysis, Cox regression with time‐dependent covariates was applied; all age groups were combined, and all baseline variables considered in the main analysis were treated as time‐dependent covariates. In the thirteenth sensitivity analysis, only subjects with available baseline information on blood pressure and low‐density lipoprotein cholesterol (LDL‐cholesterol, LDL‐C) were included, and these variables were further adjusted for in both the weighting model and the Cox regression model. In the fourteenth sensitivity analysis, only subjects without a history of albuminuria (urine albumin‐to‐creatinine ratio [UACR] ≥ 3 mg/mmol) at baseline were included. CKD was defined based on diagnosis records, eGFR <60 mL/min/1.73 m^2^, or UACR ≥3 mg/mmol. In the fifteenth sensitivity analysis, hypertension was defined as the presence of a diagnosis of hypertension, elevated blood pressure (meeting the guideline‐recommended threshold of systolic ≥140 mmHg and/or diastolic ≥90 mmHg), or the use of antihypertensive medications—including renin–angiotensin system agents, beta‐blockers, calcium channel blockers, and thiazide diuretics—within 1 year prior to baseline. Diuretics were further classified into thiazide diuretics and other diuretics, and both categories were adjusted for in the weighting model and the Cox regression model. Subgroup analysis by stratifying with sex (male, female) was conducted in both cohorts. Additionally, in the UK cohort, a subgroup analysis was performed based on participants' Asian descent.

All significant tests were two‐tailed and applied a significance level at *p* <0.05. All statistical analyses were performed with Stata 15.1.

## 3 RESULTS

Overall, 5 093 429 and 3 473 976 subjects were included in this study in the UK and HK cohort, respectively, including 124 610 and 183 114 subjects in the T2DM onset group, and 4 968 819 and 3 290 862 subjects in the comparison group. Tables 1 and 2 provide the details of baseline characteristics for the new‐onset T2DM and comparison groups after weighting. The SMD was <0.2 for all characteristics, indicating well balanced. Supplementary Tables 2 and 3 summarise the baseline characteristics and clinical characteristics before weighting. All variables in our models had VIF values well below the pre‐specified threshold of 5, suggesting that multicollinearity was unlikely to have affected the estimates. Detailed VIF results are provided in Supplementary Table 5.

**TABLE 1:** Baseline characteristics of subjects in different age groups after weighting for the UK cohort.

(a)									
	Age 18–39 (*N* = 2 443 787)			Age 40–49 (*N* = 965 386)			Age 50–59 (*N* = 711 892)		
	No T2DM (*N* = 2 420 308)	New onset T2DM (*N* = 23 479)	SMD	No T2DM (*N* = 945 033)	New onset T2DM (*N* = 20 353)	SMD	No T2DM (*N* = 683 301)	New onset T2DM (*N* = 28 591)	SMD
United Kingdom									
Male	1 146 096 (47.4%)	11 516 (49.0%)	0.03	487 712 (51.6%)	10 561 (51.9%)	0.01	347 409 (50.8%)	14 619 (51.1%)	0.01
Age, years	28.4 (6.2)	28.9 (6.1)	0.09	44.3 (2.8)	44.3 (2.8)	0.01	54.3 (2.9)	54.3 (2.9)	0.01
Current smoker	587 634 (24.3%)	6722 (28.6%)	0.10	215 796 (22.8%)	4494 (22.1%)	0.02	138 843 (20.3%)	5523 (19.3%)	0.03
Obesity	212 777 (8.8%)	1958 (8.3%)	0.02	158 042 (16.7%)	3349 (16.5%)	0.01	132 421 (19.4%)	5528 (19.3%)	0.00
Atrial fibrillation	984 (0.0%)	27 (0.1%)	0.03	1972 (0.2%)	75 (0.4%)	0.03	4184 (0.6%)	189 (0.7%)	0.01
Peripheral vascular disease	114 (0.0%)	6 (0.0%)	0.02	483 (0.1%)	18 (0.1%)	0.01	2227 (0.3%)	106 (0.4%)	0.01
Amputation	1843 (0.1%)	21 (0.1%)	0.00	1563 (0.2%)	25 (0.1%)	0.01	1467 (0.2%)	58 (0.2%)	0.00
Dementia	0 (0.0%)	0 (0.0%)	0.00	77 (0.0%)	1 (0.0%)	0.00	374 (0.1%)	16 (0.1%)	0.00
Chronic lung disease	652 (0.0%)	9 (0.0%)	0.01	3208 (0.3%)	47 (0.2%)	0.02	9820 (1.4%)	343 (1.2%)	0.02
Connective tissue disease	6982 (0.3%)	87 (0.4%)	0.01	7600 (0.8%)	135 (0.7%)	0.02	9726 (1.4%)	363 (1.3%)	0.01
Peptic ulcer disease	4342 (0.2%)	45 (0.2%)	0.00	7715 (0.8%)	169 (0.8%)	0.00	10 388 (1.5%)	394 (1.4%)	0.01
Liver disease	1359 (0.1%)	14 (0.1%)	0.00	1722 (0.2%)	33 (0.2%)	0.00	1895 (0.3%)	85 (0.3%)	0.00
Cardiovascular disease	2736 (0.1%)	256 (1.1%)	0.13	7143 (0.8%)	387 (1.9%)	0.10	19 413 (2.8%)	1077 (3.8%)	0.05
Hemiplegia	1546 (0.1%)	34 (0.1%)	0.03	855 (0.1%)	10 (0.1%)	0.02	830 (0.1%)	27 (0.1%)	0.01
Leukaemia	694 (0.0%)	17 (0.1%)	0.02	281 (0.0%)	6 (0.0%)	0.00	429 (0.1%)	16 (0.1%)	0.00
Malignant lymphoma	1250 (0.1%)	23 (0.1%)	0.02	1071 (0.1%)	18 (0.1%)	0.01	1135 (0.2%)	42 (0.1%)	0.00
Cancer	6042 (0.2%)	129 (0.5%)	0.05	9202 (1.0%)	123 (0.6%)	0.04	16 005 (2.3%)	622 (2.2%)	0.01
Hypertension	16 917 (0.7%)	473 (2.0%)	0.11	49 197 (5.2%)	1245 (6.1%)	0.04	96 554 (14.1%)	3962 (13.9%)	0.01
Retinopathy	1133 (0.0%)	18 (0.1%)	0.01	1343 (0.1%)	23 (0.1%)	0.01	1826 (0.3%)	73 (0.3%)	0.00
Hyperfiltration	2283 (0.1%)	30 (0.1%)	0.01	789 (0.1%)	18 (0.1%)	0.00	624 (0.1%)	31 (0.1%)	0.01
Renin‐angiotensin‐system agents	7511 (0.3%)	225 (1.0%)	0.08	30 164 (3.2%)	706 (3.5%)	0.02	63 325 (9.3%)	2696 (9.4%)	0.01
Beta blockers	20 954 (0.9%)	302 (1.3%)	0.04	24 620 (2.6%)	731 (3.6%)	0.06	40 124 (5.9%)	1884 (6.6%)	0.03
Calcium channel blockers	4073 (0.2%)	102 (0.4%)	0.05	14 473 (1.5%)	411 (2.0%)	0.04	34 445 (5.0%)	1501 (5.2%)	0.01
Diuretics	4443 (0.2%)	140 (0.6%)	0.07	16 110 (1.7%)	444 (2.2%)	0.03	38 426 (5.6%)	1722 (6.0%)	0.02
Thiazide diuretics	12 537 (0.5%)	340 (1.4%)	0.09	35 702 (3.8%)	950 (4.7%)	0.04	67 030 (9.8%)	2737 (9.6%)	0.01
Other diuretics	15 265 (0.6%)	459 (2.0%)	0.12	24 686 (2.6%)	983 (4.8%)	0.12	37 365 (5.5%)	2306 (8.1%)	0.10
Statin	3553 (0.1%)	126 (0.5%)	0.07	19 702 (2.1%)	586 (2.9%)	0.05	56 263 (8.2%)	2474 (8.7%)	0.02
Fibrate	176 (0.0%)	11 (0.0%)	0.02	663 (0.1%)	29 (0.1%)	0.02	1232 (0.2%)	66 (0.2%)	0.01
Other lipid lowering agents	459 (0.0%)	38 (0.2%)	0.05	1416 (0.1%)	65 (0.3%)	0.04	3519 (0.5%)	184 (0.6%)	0.02

(b)									
	Age 60–69 (*N* = 535 743)			Age 70–79 (*N* = 271 408)			Age ≥80 (*N* = 165 191)		
	No T2DM (*N* = 506 387)	New onset T2DM (*N* = 29 356)	SMD	No T2DM (*N* = 254 841)	New onset T2DM (*N* = 16 567)	SMD	No T2DM (*N* = 158 927)	New onset T2DM (*N* = 6264)	SMD
United Kingdom									
Male	254 907 (50.3%)	14 588 (49.7%)	0.01	117 125 (46.0%)	7523 (45.4%)	0.01	52 989 (33.3%)	2044 (32.6%)	0.02
Age, years	63.9 (2.8)	63.9 (2.8)	0.01	74.0 (2.9)	74.0 (2.8)	0.01	85.6 (4.6)	85.4 (4.5)	0.04
Current smoker	79 897 (15.8%)	4257 (14.5%)	0.04	26 715 (10.5%)	1585 (9.6%)	0.03	8349 (5.3%)	303 (4.8%)	0.02
Obesity	97 683 (19.3%)	5561 (18.9%)	0.01	42 737 (16.8%)	2767 (16.7%)	0.00	12 651 (8.0%)	498 (7.9%)	0.00
Atrial fibrillation	8630 (1.7%)	518 (1.8%)	0.00	11 261 (4.4%)	745 (4.5%)	0.00	13 523 (8.5%)	545 (8.7%)	0.01
Peripheral vascular disease	4877 (1.0%)	310 (1.1%)	0.01	4779 (1.9%)	337 (2.0%)	0.01	3443 (2.2%)	142 (2.3%)	0.01
Amputation	1496 (0.3%)	91 (0.3%)	0.00	1060 (0.4%)	76 (0.5%)	0.01	858 (0.5%)	34 (0.5%)	0.00
Dementia	1540 (0.3%)	86 (0.3%)	0.00	5492 (2.2%)	354 (2.1%)	0.00	15 477 (9.7%)	574 (9.2%)	0.02
Chronic lung disease	19 374 (3.8%)	1088 (3.7%)	0.01	16 992 (6.7%)	1042 (6.3%)	0.02	9429 (5.9%)	369 (5.9%)	0.00
Connective tissue disease	11 045 (2.2%)	661 (2.2%)	0.00	8960 (3.5%)	570 (3.4%)	0.00	6966 (4.4%)	279 (4.5%)	0.00
Peptic ulcer disease	11 181 (2.2%)	667 (2.3%)	0.00	8183 (3.2%)	498 (3.0%)	0.01	5307 (3.3%)	188 (3.0%)	0.02
Liver disease	1460 (0.3%)	89 (0.3%)	0.00	674 (0.3%)	46 (0.3%)	0.00	278 (0.2%)	10 (0.2%)	0.00
Cardiovascular disease	34 213 (6.8%)	2500 (8.5%)	0.07	33 888 (13.3%)	2425 (14.6%)	0.04	28 965 (18.2%)	1299 (20.7%)	0.06
Hemiplegia	737 (0.1%)	49 (0.2%)	0.01	529 (0.2%)	40 (0.2%)	0.01	353 (0.2%)	14 (0.2%)	0.00
Leukaemia	688 (0.1%)	32 (0.1%)	0.01	599 (0.2%)	44 (0.3%)	0.01	455 (0.3%)	19 (0.3%)	0.00
Malignant lymphoma	1403 (0.3%)	81 (0.3%)	0.00	979 (0.4%)	56 (0.3%)	0.01	577 (0.4%)	22 (0.3%)	0.00
Cancer	24 146 (4.8%)	1329 (4.5%)	0.01	19 269 (7.6%)	1202 (7.3%)	0.01	13 196 (8.3%)	540 (8.6%)	0.01
Hypertension	125 116 (24.7%)	7650 (26.1%)	0.03	88 852 (34.9%)	5962 (36.0%)	0.02	56 647 (35.6%)	2442 (39.0%)	0.07
Retinopathy	2131 (0.4%)	114 (0.4%)	0.01	1712 (0.7%)	117 (0.7%)	0.00	1242 (0.8%)	55 (0.9%)	0.01
Hyperfiltration	482 (0.1%)	36 (0.1%)	0.01	228 (0.1%)	18 (0.1%)	0.01	137 (0.1%)	7 (0.1%)	0.01
Renin‐angiotensin‐system agents	78 506 (15.5%)	4742 (16.2%)	0.02	53 191 (20.9%)	3541 (21.4%)	0.01	27 028 (17.0%)	1090 (17.4%)	0.01
Beta blockers	51 949 (10.3%)	3134 (10.7%)	0.01	36 993 (14.5%)	2394 (14.4%)	0.00	19 793 (12.5%)	828 (13.2%)	0.02
Calcium channel blockers	55 043 (10.9%)	3427 (11.7%)	0.03	43 893 (17.2%)	2971 (17.9%)	0.02	25 519 (16.1%)	1054 (16.8%)	0.02
Diuretics	60 427 (11.9%)	3651 (12.4%)	0.02	51 825 (20.3%)	3372 (20.4%)	0.00	37 848 (23.8%)	1508 (24.1%)	0.01
Thiazide diuretics	92 302 (18.2%)	4892 (16.7%)	0.04	67 043 (26.3%)	3661 (22.1%)	0.10	35 663 (22.4%)	1171 (18.7%)	0.09
Other diuretics	58 018 (11.5%)	4087 (13.9%)	0.07	61 286 (24.0%)	3964 (23.9%)	0.00	56 820 (35.8%)	2260 (36.1%)	0.01
Statin	93 838 (18.5%)	5593 (19.1%)	0.01	68 521 (26.9%)	4494 (27.1%)	0.01	26 756 (16.8%)	1059 (16.9%)	0.00
Fibrate	1531 (0.3%)	111 (0.4%)	0.01	1030 (0.4%)	67 (0.4%)	0.00	301 (0.2%)	13 (0.2%)	0.00
Other lipid lowering agents	5567 (1.1%)	370 (1.3%)	0.01	3374 (1.3%)	226 (1.4%)	0.00	851 (0.5%)	38 (0.6%)	0.01

*Note*: All parameters are expressed in either number (percentage) or mean (SD).

Abbreviations: DM, diabetes mellitus; SMD, standard mean difference.

**TABLE 2:** Baseline characteristics of subjects in different age groups after weighting for the HK cohort.

(a)									
	Age 18–39 (*N* = 1 285 221)			Age 40–49 (*N* = 707 828)			Age 50–59 (*N* = 711 418)		
	No T2DM (*N* = 1 276 436)	New onset T2DM (*N* = 8785)	SMD	No T2DM (*N* = 680 891)	New onset T2DM (*N* = 26 937)	SMD	No T2DM (*N* = 653 332)	New onset T2DM (*N* = 58 086)	SMD
Hong Kong									
Male	564 188 (44.2%)	3930 (44.7%)	0.01	289 158 (42.5%)	11 565 (42.9%)	0.01	306 064 (46.8%)	27 808 (47.9%)	0.02
Age, years	29.0 (6.2)	28.9 (6.2)	0.01	44.8 (2.9)	44.8 (2.9)	0.01	54.2 (2.9)	54.2 (2.8)	0.02
Current smoker	4432 (0.3%)	30 (0.3%)	0.00	4109 (0.6%)	162 (0.6%)	0.00	5796 (0.9%)	518 (0.9%)	0.00
Obesity	33 203 (2.6%)	235 (2.7%)	0.00	53 142 (7.8%)	1929 (7.2%)	0.02	70 722 (10.8%)	6097 (10.5%)	0.01
Atrial fibrillation	444 (0.0%)	12 (0.1%)	0.03	1135 (0.2%)	47 (0.2%)	0.00	3033 (0.5%)	318 (0.5%)	0.01
Peripheral vascular disease	99 (0.0%)	1 (0.0%)	0.00	194 (0.0%)	9 (0.0%)	0.00	409 (0.1%)	44 (0.1%)	0.01
Amputation	510 (0.0%)	5 (0.1%)	0.01	459 (0.1%)	27 (0.1%)	0.01	493 (0.1%)	47 (0.1%)	0.00
Dementia	0 (0.0%)	0 (0.0%)	0.00	51 (0.0%)	0 (0.0%)	0.01	195 (0.0%)	20 (0.0%)	0.00
Chronic lung disease	571 (0.0%)	6 (0.1%)	0.01	767 (0.1%)	25 (0.1%)	0.01	2723 (0.4%)	220 (0.4%)	0.01
Connective tissue disease	1644 (0.1%)	33 (0.4%)	0.05	1316 (0.2%)	68 (0.3%)	0.01	1375 (0.2%)	130 (0.2%)	0.00
Peptic ulcer disease	4039 (0.3%)	15 (0.2%)	0.03	4193 (0.6%)	171 (0.6%)	0.00	6998 (1.1%)	614 (1.1%)	0.00
Liver disease	3959 (0.3%)	31 (0.4%)	0.01	4571 (0.7%)	167 (0.6%)	0.01	6348 (1.0%)	546 (0.9%)	0.00
Cardiovascular disease	2143 (0.2%)	130 (1.5%)	0.15	7326 (1.1%)	778 (2.9%)	0.13	19 891 (3.0%)	2652 (4.6%)	0.08
Hemiplegia	993 (0.1%)	9 (0.1%)	0.01	973 (0.1%)	83 (0.3%)	0.03	2001 (0.3%)	273 (0.5%)	0.03
Leukaemia	493 (0.0%)	6 (0.1%)	0.01	331 (0.0%)	8 (0.0%)	0.01	323 (0.0%)	21 (0.0%)	0.01
Malignant lymphoma	301 (0.0%)	1 (0.0%)	0.01	234 (0.0%)	5 (0.0%)	0.01	409 (0.1%)	26 (0.0%)	0.01
Cancer	7305 (0.6%)	59 (0.7%)	0.01	12 959 (1.9%)	393 (1.5%)	0.03	18 664 (2.9%)	1344 (2.3%)	0.03
Hypertension	6560 (0.5%)	129 (1.5%)	0.10	40 172 (5.9%)	1729 (6.4%)	0.02	100 893 (15.4%)	8614 (14.8%)	0.02
Retinopathy	49 (0.0%)	2 (0.0%)	0.02	114 (0.0%)	15 (0.1%)	0.02	342 (0.1%)	54 (0.1%)	0.02
Hyperfiltration	7642 (0.6%)	44 (0.5%)	0.01	143 (0.0%)	5 (0.0%)	0.00	39 (0.0%)	4 (0.0%)	0.00
Renin‐angiotensin‐system agents	3928 (0.3%)	81 (0.9%)	0.08	16 102 (2.4%)	840 (3.1%)	0.05	35 036 (5.4%)	3554 (6.1%)	0.03
Beta blockers	16 056 (1.3%)	216 (2.5%)	0.09	33 037 (4.9%)	1896 (7.0%)	0.09	66 013 (10.1%)	6563 (11.3%)	0.04
Calcium channel blockers	6245 (0.5%)	132 (1.5%)	0.10	27 067 (4.0%)	1226 (4.6%)	0.03	63 768 (9.8%)	5773 (9.9%)	0.01
Diuretics	2524 (0.2%)	79 (0.9%)	0.10	10 208 (1.5%)	534 (2.0%)	0.04	26 673 (4.1%)	2564 (4.4%)	0.02
Thiazide diuretics	745 (0.1%)	15 (0.2%)	0.03	4609 (0.7%)	199 (0.7%)	0.01	12 635 (1.9%)	1088 (1.9%)	0.00
Other diuretics	1820 (0.1%)	67 (0.8%)	0.09	5780 (0.8%)	340 (1.3%)	0.04	14 491 (2.2%)	1506 (2.6%)	0.02
Statin	1287 (0.1%)	32 (0.4%)	0.05	6337 (0.9%)	376 (1.4%)	0.04	18 457 (2.8%)	1985 (3.4%)	0.03
Fibrate	198 (0.0%)	10 (0.1%)	0.04	1234 (0.2%)	93 (0.3%)	0.03	3071 (0.5%)	347 (0.6%)	0.02
Other lipid lowering agents	72 (0.0%)	1 (0.0%)	0.01	141 (0.0%)	11 (0.0%)	0.01	302 (0.0%)	58 (0.1%)	0.02

(b)									
	Age 60–69 (*N* = 396 830)			Age 70–79 (*N* = 257 281)			Age ≥80 (*N* = 115 133)		
	No T2DM (*N* = 345 676)	New onset T2DM (*N* = 51 154)	SMD	No T2DM (*N* = 228 629)	New onset T2DM (*N* = 28 652)	SMD	No T2DM (*N* = 105 667)	New onset T2DM (*N* = 9466)	SMD
Hong Kong									
Male	179 467 (51.9%)	26 547 (51.9%)	0.00	114 495 (50.1%)	14 043 (49.0%)	0.02	41 184 (39.0%)	3709 (39.2%)	0.00
Age, years	63.8 (2.9)	63.8 (2.8)	0.01	74.1 (2.8)	74.1 (2.8)	0.01	84.5 (4.1)	84.4 (4.0)	0.03
Current smoker	4496 (1.3%)	666 (1.3%)	0.00	3177 (1.4%)	353 (1.2%)	0.01	758 (0.7%)	59 (0.6%)	0.01
Obesity	41 708 (12.1%)	6511 (12.7%)	0.02	22 260 (9.7%)	2994 (10.5%)	0.02	5657 (5.4%)	538 (5.7%)	0.01
Atrial fibrillation	3850 (1.1%)	503 (1.0%)	0.01	4991 (2.2%)	637 (2.2%)	0.00	4020 (3.8%)	344 (3.6%)	0.01
Peripheral vascular disease	422 (0.1%)	72 (0.1%)	0.01	479 (0.2%)	70 (0.2%)	0.01	319 (0.3%)	31 (0.3%)	0.00
Amputation	183 (0.1%)	18 (0.0%)	0.01	123 (0.1%)	19 (0.1%)	0.01	86 (0.1%)	3 (0.0%)	0.02
Dementia	510 (0.1%)	64 (0.1%)	0.01	2055 (0.9%)	247 (0.9%)	0.00	4083 (3.9%)	356 (3.8%)	0.01
Chronic lung disease	5208 (1.5%)	737 (1.4%)	0.01	9608 (4.2%)	1155 (4.0%)	0.01	6428 (6.1%)	617 (6.5%)	0.02
Connective tissue disease	615 (0.2%)	71 (0.1%)	0.01	440 (0.2%)	47 (0.2%)	0.01	154 (0.1%)	12 (0.1%)	0.01
Peptic ulcer disease	5348 (1.5%)	688 (1.3%)	0.02	5381 (2.4%)	641 (2.2%)	0.01	3592 (3.4%)	309 (3.3%)	0.01
Liver disease	3881 (1.1%)	573 (1.1%)	0.00	2473 (1.1%)	298 (1.0%)	0.00	1117 (1.1%)	96 (1.0%)	0.00
Cardiovascular disease	24 343 (7.0%)	3728 (7.3%)	0.01	28 205 (12.3%)	3706 (12.9%)	0.02	18 080 (17.1%)	1943 (20.5%)	0.09
Hemiplegia	2342 (0.7%)	357 (0.7%)	0.00	2749 (1.2%)	385 (1.3%)	0.01	2005 (1.9%)	214 (2.3%)	0.03
Leukaemia	148 (0.0%)	22 (0.0%)	0.00	65 (0.0%)	7 (0.0%)	0.00	33 (0.0%)	3 (0.0%)	0.00
Malignant lymphoma	281 (0.1%)	37 (0.1%)	0.00	242 (0.1%)	24 (0.1%)	0.01	88 (0.1%)	7 (0.1%)	0.00
Cancer	12 801 (3.7%)	1667 (3.3%)	0.02	11 571 (5.1%)	1330 (4.6%)	0.02	5598 (5.3%)	449 (4.7%)	0.03
Hypertension	94 359 (27.3%)	13 674 (26.7%)	0.01	84 153 (36.8%)	10 139 (35.4%)	0.03	42 066 (39.8%)	3622 (38.3%)	0.03
Retinopathy	392 (0.1%)	69 (0.1%)	0.01	326 (0.1%)	52 (0.2%)	0.01	131 (0.1%)	15 (0.2%)	0.01
Hyperfiltration	13 (0.0%)	2 (0.0%)	0.00	5 (0.0%)	1 (0.0%)	0.00	3 (0.0%)	0 (0.0%)	0.01
Renin‐angiotensin‐system agents	31 020 (9.0%)	4544 (8.9%)	0.00	25 902 (11.3%)	3418 (11.9%)	0.02	12 317 (11.7%)	1209 (12.8%)	0.03
Beta blockers	55 556 (16.1%)	7956 (15.6%)	0.01	45 641 (20.0%)	5645 (19.7%)	0.01	18 685 (17.7%)	1751 (18.5%)	0.02
Calcium channel blockers	62 005 (17.9%)	9347 (18.3%)	0.01	58 828 (25.7%)	7476 (26.1%)	0.01	30 931 (29.3%)	2714 (28.7%)	0.01
Diuretics	25 885 (7.5%)	3804 (7.4%)	0.00	27 070 (11.8%)	3337 (11.6%)	0.01	14 773 (14.0%)	1373 (14.5%)	0.01
Thiazide diuretics	12 264 (3.5%)	1712 (3.3%)	0.01	11 363 (5.0%)	1311 (4.6%)	0.02	5387 (5.1%)	435 (4.6%)	0.02
Other diuretics	14 078 (4.1%)	2153 (4.2%)	0.01	16 296 (7.1%)	2092 (7.3%)	0.01	9698 (9.2%)	972 (10.3%)	0.04
Statin	20 939 (6.1%)	3251 (6.4%)	0.01	16 354 (7.2%)	2123 (7.4%)	0.01	5602 (5.3%)	542 (5.7%)	0.02
Fibrate	2936 (0.8%)	459 (0.9%)	0.01	2418 (1.1%)	315 (1.1%)	0.00	831 (0.8%)	90 (0.9%)	0.02
Other lipid lowering agents	236 (0.1%)	48 (0.1%)	0.01	175 (0.1%)	27 (0.1%)	0.01	54 (0.1%)	8 (0.1%)	0.01

*Note*: All parameters are expressed in either number (percentage) or mean (SD).

Abbreviations: DM, diabetes mellitus; SMD, standard mean difference.

**FIGURE 1:**
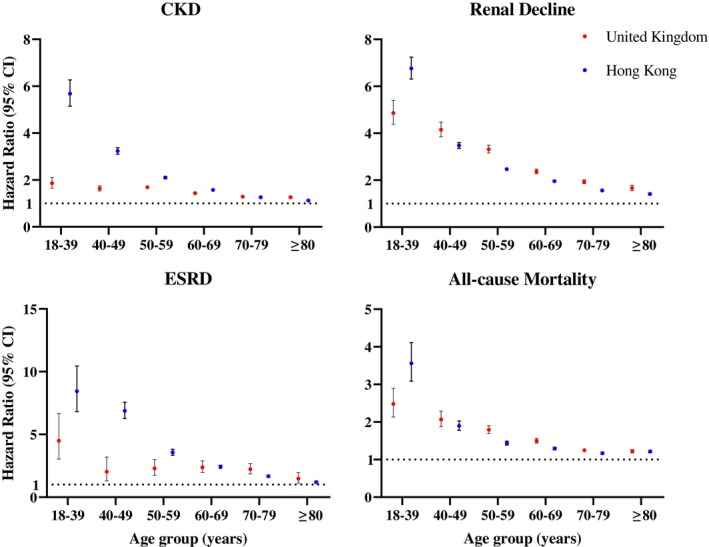
The association between onset of diabetes mellitus and kidney disease/mortality in different age groups using Cox regression. Hazard ratio with 95% confidence interval adjusted by age, sex, smoking status, comorbidities (i.e., obesity, atrial fibrillation, peripheral vascular disease, amputation, dementia, chronic lung disease, connective tissue disease, peptic ulcer disease, liver disease, cardiovascular disease, hemiplegia, leukaemia, malignant lymphoma, cancer, hypertension, retinopathy, and hyperfiltration), and the use of renin‐angiotensin system agents, beta‐blockers, calcium channel blockers, diuretics, statins, fibrates, other lipid‐lowering agents, and weighting. CI, confidence interval, CKD, chronic kidney disease; ESRD, end‐stage renal disease.

After 11.6 median follow‐up years (around 57 million person‐years) in the UK cohort and 9.7 median follow‐up years (around 35 million person‐years) in the HK cohort, a total of 323 597 and 395 025 CKD, 103 441 and 346 508 renal decline, 5367 and 38 029 ESRD were recorded in the UK and HK cohorts, respectively. The incidence rates of each outcome by age groups are listed in Table 3. The incidence rates of all outcomes increased with age and were higher in the exposed group than in the comparison group. In the UK cohort, the incidence rate (95% CI) per 10 000 person‐years for CKD was 18.27 (14.55, 23.27) in the exposed group and 9.74 (9.60, 9.89) in the comparison group; 25.58 (20.13, 33.02) in the exposed group and 4.68 (4.57, 4.78) in the comparison group for renal decline; and 1.79 (0.77, 5.15) in the exposed group and 0.33 (0.30, 0.35) in the comparison group for ESRD at age 18–39. While at age 80 or above, the incidence rate (95% CI) per 10 000 person‐years for CKD was 1217.31 (1149.31, 1289.60) in the exposed group and 864.91 (856.66, 873.25) in the comparison group; 325.66 (299.42, 354.72) in the exposed group and 182.33 (179.38, 185.34) in the comparison group for renal decline; and 19.02 (13.45, 27.80) in the exposed group and 12.05 (11.34, 12.82) in the comparison group for ESRD. Table 2 and Figure 1 show the hazard ratios (HRs) of all outcome events and mortality for subjects with new‐onset T2DM in comparison with the subjects without DM in each age group. The patients with new‐onset T2DM had a higher risk of all outcome events and mortality in all age groups, particularly for those subjects at a younger age. The HR (95% CI) for renal decline risk was 4.87 (4.38, 5.40) for subjects with T2DM onset compared to those without at age 18–39, and it decreased to 1.66 (1.55, 1.78) at age ≥80. For CKD and ESRD, the HRs were also higher among younger subjects with onset of T2DM at age 18–39 (CKD, 1.87 (1.65, 2.11); ESRD, HR: 4.49 (3.03, 6.68)) compared with those at age≥80 (CKD, 1.26 (1.21, 1.32); ESRD, HR: 1.48 (1.12, 1.96)). Results for the HK cohort showed a similar pattern, with the risk for all four outcomes decreasing with increasing age. The HR (95% CI) of CKD in patients with new onset of T2DM was 5.68 (5.15, 6.27) at age 18–39 and 1.13 (1.10, 1.16) at age ≥80. Regarding ESRD, the HR (95% CI) was 8.44 (6.81, 10.46) at age 18–39 and 1.20 (1.11, 1.30) at age ≥80.

**TABLE 3:** Incidence rate of chronic kidney, end‐stage renal disease, and death outcomes in different age groups.

	United Kingdom					Hong Kong				
Age group	No T2DM		New onset T2DM			No T2DM		New onset T2DM		
	Event	Incidence rate^a^	Event	Incidence rate^a^	Hazard ratio^b^	Event	Incidence rate^a^	Event	Incidence rate^a^	Hazard ratio^b^
Chronic kidney disease										
18–39	16 721	9.74 (9.60, 9.89)	266	18.27 (14.55, 23.27)	1.87 (1.65, 2.11)	11 705	10.21 (10.02, 10.39)	415	60.95 (49.32, 76.20)	5.68 (5.15, 6.27)
40–49	31 467	37.71 (37.29, 38.13)	893	62.08 (56.18, 68.77)	1.64 (1.53, 1.75)	23 476	37.80 (37.31, 38.30)	2441	116.03 (109.56, 122.98)	3.24 (3.11, 3.38)
50–59	50 274	82.22 (81.49, 82.95)	2904	143.83 (135.22, 153.13)	1.69 (1.63, 1.76)	55 309	94.44 (93.62, 95.26)	7989	184.96 (179.61, 190.51)	2.10 (2.05, 2.15)
60–69	80 328	186.45 (185.13, 187.78)	5524	289.21 (277.49, 301.52)	1.44 (1.40, 1.48)	77 606	272.69 (270.69, 274.70)	13 808	400.02 (391.24, 409.03)	1.58 (1.55, 1.60)
70–79	73 513	408.24 (405.15, 411.37)	5210	591.06 (568.63, 614.49)	1.29 (1.26, 1.33)	111 715	725.02 (720.79, 729.28)	14 002	887.88 (868.34, 907.90)	1.26 (1.24, 1.28)
≥80	54 140	864.91 (856.66, 873.25)	2357	1217.31 (1149.31, 1289.60)	1.26 (1.21, 1.32)	70 633	1473.77 (1462.69, 1484.94)	5926	1677.25 (1616.73, 1740.03)	1.13 (1.10, 1.16)
Renal decline										
18–39	8045	4.68 (4.57, 4.78)	371	25.58 (20.13, 33.02)	4.87 (4.38, 5.40)	20 869	18.26 (18.02, 18.52)	850	129.09 (110.15, 152.19)	6.76 (6.31, 7.25)
40–49	10 297	12.16 (11.93, 12.40)	741	51.38 (46.04, 57.52)	4.15 (3.85, 4.47)	30 767	49.78 (49.21, 50.35)	3402	165.32 (157.27, 173.89)	3.48 (3.36, 3.60)
50–59	15 418	24.37 (23.98, 24.76)	1674	80.56 (74.40, 87.36)	3.32 (3.15, 3.49)	56 531	96.40 (95.58, 97.23)	9423	221.59 (215.55, 227.83)	2.47 (2.42, 2.53)
60–69	23 736	50.73 (50.08, 51.39)	2505	120.82 (113.99, 128.17)	2.37 (2.27, 2.47)	61 099	206.68 (204.97, 208.41)	12 758	363.14 (354.78, 371.74)	1.96 (1.92, 2.00)
70–79	22 921	107.38 (105.99, 108.79)	2225	213.35 (201.99, 225.50)	1.93 (1.85, 2.02)	81 408	460.35 (457.27, 463.46)	11 438	653.47 (637.78, 669.60)	1.56 (1.53, 1.59)
≥ 80	14 688	182.33 (179.38, 185.34)	820	325.66 (299.42, 354.72)	1.66 (1.55, 1.78)	53 046	863.62 (856.66, 870.64)	4917	1149.10 (1106.20, 1193.77)	1.41 (1.37, 1.45)
End‐stage renal disease										
18–39	562	0.33 (0.30, 0.35)	26	1.79 (0.77, 5.15)	4.49 (3.03,6.68)	1689	1.47 (1.40, 1.54)	89	12.94 (9.57, 17.93)	8.44 (6.81,10.46)
40–49	518	0.61 (0.56, 0.66)	20	1.34 (0.72, 2.77)	2.03 (1.30,3.19)	2397	3.81 (3.66, 3.97)	538	24.78 (21.88, 28.18)	6.89 (6.26,7.57)
50–59	735	1.15 (1.07, 1.24)	57	2.65 (1.75, 4.23)	2.29 (1.75,3.00)	4488	7.43 (7.20, 7.66)	1123	24.68 (22.84, 26.72)	3.57 (3.34,3.82)
60–69	1022	2.13 (2.01, 2.27)	112	5.15 (3.93, 6.87)	2.38 (1.96,2.90)	5392	17.17 (16.70, 17.67)	1397	35.88 (33.44, 38.54)	2.39 (2.25,2.54)
70–79	1109	4.97 (4.69, 5.28)	131	11.65 (8.99, 15.37)	2.23 (1.86,2.68)	9966	50.01 (49.02, 51.03)	1510	72.89 (68.15, 78.04)	1.67 (1.58,1.76)
≥80	1023	12.05 (11.34, 12.82)	52	19.02 (13.45, 27.80)	1.48 (1.12,1.96)	8747	120.15 (117.66, 122.70)	693	129.73 (117.44, 143.67)	1.20 (1.11,1.30)
All‐cause mortality										
18–39	6799	3.94 (3.85, 4.04)	169	11.52 (8.72, 15.53)	2.48 (2.13, 2.89)	8734	7.59 (7.43, 7.75)	191	27.49 (19.28, 40.55)	3.56 (3.08, 4.11)
40–49	10 791	12.67 (12.43, 12.91)	394	26.64 (23.04, 30.97)	2.07 (1.87, 2.29)	15 089	23.99 (23.61, 24.38)	952	43.62 (39.82, 47.87)	1.90 (1.78, 2.03)
50–59	20 279	31.67 (31.23, 32.11)	1216	56.61 (51.69, 62.13)	1.79 (1.69, 1.90)	31 189	51.55 (50.97, 52.14)	3268	71.45 (68.24, 74.86)	1.44 (1.38, 1.49)
60–69	36 299	75.78 (74.99, 76.57)	2529	116.07 (109.48, 123.15)	1.50 (1.44, 1.56)	36 579	116.23 (115.00, 117.47)	5463	139.44 (134.56, 144.54)	1.29 (1.26, 1.33)
70–79	46 625	208.89 (206.98, 210.82)	3092	274.89 (261.86, 288.70)	1.25 (1.20, 1.30)	63 743	317.89 (315.46, 320.35)	7069	337.83 (327.43, 348.63)	1.17 (1.14, 1.20)
≥80	66 272	779.18 (772.77, 785.65)	2742	1003.24 (953.04, 1056.30)	1.22 (1.18, 1.27)	61 994	841.48 (835.23, 847.79)	5069	938.49 (903.65, 974.82)	1.21 (1.18, 1.25)

^a^ Incidence rate (cases/10 000 person‐years) with 95% confidence interval based on Poisson distribution.

^b^ Hazard ratio with 95% confidence interval adjusted by age, sex, smoking status, comorbidities (i.e., obesity, atrial fibrillation, peripheral vascular disease, amputation, dementia, chronic lung disease, connective tissue disease, peptic ulcer disease, liver disease, cardiovascular disease, hemiplegia, leukaemia, malignant lymphoma, cancer, hypertension, retinopathy, and hyperfiltration), and the use of renin‐angiotensin system agents, beta‐blockers, calcium channel blockers, diuretics, statins, fibrates, other lipid‐lowering agents, and weighting.

**FIGURE 2:**
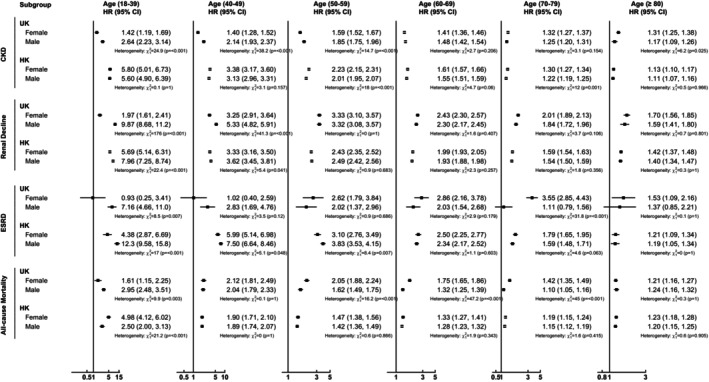
Association between new onset T2DM and risk of kidney disease and mortality within subgroups stratified by gender. Hazard ratio with 95% confidence interval adjusted by age, smoking status, comorbidities (i.e., obesity, atrial fibrillation, peripheral vascular disease, amputation, dementia, chronic lung disease, connective tissue disease, peptic ulcer disease, liver disease, cardiovascular disease, hemiplegia, leukaemia, malignant lymphoma, cancer, hypertension, retinopathy, and hyperfiltration), and the use of renin–angiotensin system agents, beta‐blockers, calcium channel blockers, diuretics, statins, fibrates, other lipid‐lowering agents, and weighting. CI, confidence interval; CKD, chronic kidney disease; ESRD, end stage renal disease.

**FIGURE 3:**
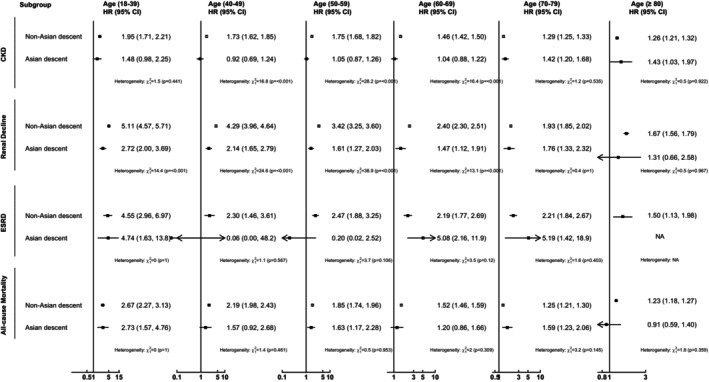
Association between new onset T2DM and risk of kidney disease and mortality within subgroups stratified by Asian descent in the United Kingdom cohort. Hazard ratio with 95% confidence interval adjusted by age, sex, smoking status, comorbidities (i.e., obesity, atrial fibrillation, peripheral vascular disease, amputation, dementia, chronic lung disease, connective tissue disease, peptic ulcer disease, liver disease, cardiovascular disease, hemiplegia, leukaemia, malignant lymphoma, cancer, hypertension, retinopathy, and hyperfiltration), and the use of renin‐angiotensin system agents, beta‐blockers, calcium channel blockers, diuretics, statins, fibrates, other lipid‐lowering agents, and weighting. CI, confidence interval; CKD, chronic kidney disease; ESRD, end‐stage renal disease; NA, not applicable due to insufficient number of cases.

The associations between new‐onset T2DM and risk of all outcome events across different age groups, stratified by sex and Asian descent, are presented in Figures 2 and 3. The patterns of risk of CKD and mortality were similar to those observed in the main analysis within the sex subgroup, with lower HRs among females aged 18–39 and 40–49 in the UK cohort. Similar patterns were observed among Asian and non‐Asian subjects in the UK cohort, except for an irregular trend in ESRD risk and nonsignificant associations with CKD risk in the non‐Asian subgroup. The supplementary figures display the results of sensitivity analyses, which demonstrated similar patterns of decreasing risk of all outcome events with increasing age.

## 4 DISCUSSION

This large‐scale retrospective cohort study, comparing participants with and without T2DM in Hong Kong and the United Kingdom, demonstrated a heightened risk of CKD, renal decline, and ESRD across age groups, and that a younger age of T2DM onset was associated with higher risks. Compared to age‐matched individuals without DM in the United Kingdom and Hong Kong, patients with early‐onset T2DM diagnosed before 40 years old had up to 2–6‐times higher risk of CKD, 5–7‐times higher risk of renal decline, and 4–8‐times higher ESRD risk, respectively. Renal disease risks decreased with increasing T2DM onset age in both Hong Kong and the United Kingdom, with participants with T2DM onset age ≥80 having less than 2‐times increased risks of CKD, renal decline, and ESRD compared to age‐matched individuals without DM. Similar trends were observed in data from both Hong Kong and the United Kingdom, although a less pronounced difference in CKD risks at various ages of onset was noted in the United Kingdom. Early‐onset DM significantly impacts individuals' physical health in terms of CKD, renal decline, and ESRD, suggesting the need to prioritise, monitor, and advocate for early disease control and regular screening of renal complications among young patients to achieve tertiary prevention. Current DM management for older patients should also be strengthened to prevent CKD complications, given the high incidence rates observed among older groups.

A recent study in Hong Kong compared the risks of CKD in patients diagnosed with T2DM 5 years apart and found an increased risk with longer T2DM duration within each age group, which diminished with increasing age.^13^ In contrast, our current study adopted a different approach by examining the differences in CKD risks between participants with and without DM across different age groups. This method provided a more valid and direct assessment of the heightened CKD risks in patients with DM of different onset ages, supporting the conclusion of the previous study. Additionally, a longer follow‐up period of 9.6 years was utilised in the current study as compared to 6.3 years in the previous study, which can better capture the development of chronic renal complications over extended periods. Similar findings were observed in another study in the Chinese population, where an earlier onset age of T2DM was associated with a higher risk of progressing to ESRD in patients with known diabetic kidney disease.^11^ Alternatively, a study conducted in Australia focused on the effects of the duration and age of onset of T2DM on the incidence of ESRD. It reported a lower incidence rate of ESRD in patients with early‐onset T2DM compared to those with late‐onset T2DM in the initial10–15 years, followed by a higher incidence rate afterwards.^12^ The initial lower incidence of ESRD in early‐onset T2DM was possibly due to the lack of non‐DM controls in the study, which hindered adjustments for baseline ESRD risks at older ages, given that the incidence of ESRD in both DM and non‐DM participants typically increases with age.

Data from the United Kingdom revealed lower risks of CKD and ESRD compared to Hong Kong among patients with early‐onset T2DM, resulting in a less significant difference in CKD and ESRD risks between early‐onset and late‐onset T2DM. The lower risks may be explained by the difference in the source of data and ethnic disparities between the two cohorts. The data for the UK cohort were collected at primary care clinics across the country, covering the general population, whereas those for the Hong Kong cohort were extracted from the electronic health records of the Hospital Authority, which provides healthcare services in the public setting. The characteristics of patients receiving private and public health care can vary by income, symptom presentation, and health awareness, with poorer attributes often observed in the latter group. This may explain the generally lower incidence rates of outcomes in the UK cohort. On the other hand, the majority of the Hong Kong population is of Chinese origin. Several multi‐ethnic studies generally demonstrated a higher incidence rate of renal complications, such as proteinuria and ESRD, in Chinese DM patients compared to other populations.^42^, ^43^, ^44^ Although our subgroup analysis did not show an increased risk of renal outcomes among Asian subjects in the UK cohort—likely due to the low incidence of events—ethnic differences in disease profile may be attributable to genetic and environmental factors, including healthcare policies and sociocultural practices such as dietary preferences affecting salt intake.^44^, ^45^ While the United Kingdom has a smaller proportion of Chinese residents compared to Hong Kong, leading to lower renal complication risks among patients with T2DM, both the UK and HK cohorts showed higher risks of renal complications among a younger age of T2DM onset. The current study, involving a large sample size from Hong Kong and the United Kingdom, aimed to enhance the generalisability by including individuals with and without DM for fair age‐matched comparisons. By examining the changes in renal function using multiple outcomes, such as renal decline, CKD, and ESRD, our study provided insight into the impact of onset age of DM on overall renal complications.

The mechanism by which the onset age of DM affects the risks of renal complications has not been clearly elucidated. Genetic factors that predispose individuals to both early‐onset DM^46^ and related microvascular or macrovascular complications^47^, ^48^ have been suggested. Apart from monogenic DM such as maturity onset diabetes of the young, DM often arises from a complex interplay of genetic and environmental factors, with polygenic inheritance influencing the onset age of T2DM through beta‐cell dysfunction.^49^ Various genetic variants associated with diabetic nephropathy, including CKD and ESRD, have been identified,^50^, ^51^ such as the *UMOD* locus.^50^, ^52^ The age of onset of T2DM and the susceptibility to DM complications are significantly influenced by hereditary genome differences, which may explain the potential association between early‐onset of DM and renal risks. Other possible causes include inherent differences between younger and older people, including psychosocial factors and awareness of health conditions. Older adults, as compared to working‐age adults, may have more free time to adopt a healthy lifestyle, such as healthy eating and sufficient physical activity, and adhere to medical interventions,^53^ leading to better glycaemic control that prevents progression to diabetic nephropathy after DM onset. This contrast may be exacerbated by the potential lack of awareness of DM status in young people, as early stages of T2DM can be asymptomatic^54^ and those with good past health may not seek medical care due to the absence of physical symptoms. This may also explain the lower risks observed among young females in the subgroup analysis since they are generally more health conscious than their male counterparts.^55^ Differences between young and old participants were supported by variations in baseline characteristics among patients with new onset T2DM, including body mass index, fasting glucose levels, and haemoglobin A1c, which all decreased with increasing onset age in this study. These findings suggest a poorer lifestyle and suboptimal glycaemic control in patients with an earlier DM onset age, consistent with previous studies demonstrating poorer glycaemic control in patients with early‐onset DM.^56^, ^57^ Poor glycaemic control in young people with DM may in turn lead to long‐term hyperglycaemia, resulting in vascular complications including diabetic nephropathy.^58^, ^59^, ^60^ Human physiology changes with age, with hyperresponsive beta‐cells being characteristics of youth, leading to a quicker decline in function when triggered, as well as a faster deterioration in glycaemic control and a poorer response to oral glucose‐lowering drugs in patients with early‐onset T2DM.^61^, ^62^ There is also natural deterioration of physiological functions with aging, which may contribute to a higher incidence of renal complications among older patients regardless of diabetes status, resulting in lower relative HRs in these age groups. It is recommended to educate the young T2DM patients on the heightened risks of CKD complications and promote the adoption of a healthy lifestyle including diet and exercise for glycaemic control.

To identify the impact of the onset age of T2DM on the risks of renal diseases, this study evaluated the effects of the onset age of DM in a large population of around 3.5 million participants from Hong Kong and 4.9 million participants from the United Kingdom. This increases generalisability by incorporating two distinct populations, each with a large sample size that is representative of the general population in their respective localities, potentially eliminating the effects of individual differences among participants, such as lifestyle factors. However, a causal relationship was not determined, and the underlying mechanism of this association remains to be clarified. The follow‐up period, with a median of 9.6 years, may not suffice for identifying long‐term complications, which could lead to underestimated risks of chronic diseases with an insidious onset, including ESRD. The two electronic health record databases have their own weaknesses. The data source of the UK cohort was from hundreds of practices, resulting in lower data completion, such as eGFR, whereas the HK cohort only represented the residents using public healthcare services. The two databases also lacked information on factors such as diet, sedentary lifestyle, socioeconomic status, diabetes treatment intensity, treatment adherence, and potential hereditary factors, which may differ by age group and contribute to the progression of T2DM and the development of complications such as CKD. The frequency of eGFR testing may also differ by age and T2DM onset group, potentially introducing bias in the identification of renal outcomes. Due to the very low completion rate of HbA1c in the comparison group (less than 5%), HbA1c was not included as a covariate in the primary analyses. This should be considered when interpreting the results. The propensity matching was also limited by these unmeasured confounders. There is survivorship bias—particularly among older age groups—due to the potential exclusion or underrepresentation of individuals who experience early events or mortality. To enhance the understanding of the association between the onset age of T2DM and renal complications, future studies examining its causality and biological mechanism should be performed. The scope of kidney outcomes can be expanded to include renal artery stenosis and kidney stones for a more comprehensive investigation. The impact of ethnicity on renal complications could also be further analysed by stratifying participants by ethnic origin, as well as by including global data that would provide a larger sample size and a more diversified population.

## 5 CONCLUSION

In this large‐scale cohort study in Hong Kong and the United Kingdom, early T2DM onset was identified to be associated with worse physical health outcomes, including increased renal complications for patients, which are especially important given the current rising trend of early‐onset DM and the significant global burden of renal complications of DM in terms of quality of life. Such effects necessitate increased awareness and control of DM and related kidney complications in order to prevent further health deterioration. This includes preventing DM from a young age and prioritising management of early‐onset T2DM.

## AUTHOR CONTRIBUTIONS

BW and KL contributed to the study design, interpretation of the results, and prepared the first draft of the manuscript. EYFW and BW contributed to the acquisition of data, statistical analysis, and interpretation of the results. All authors contributed to the interpretation of the analysis, critically reviewed and revised the manuscript, and approved the final manuscript as submitted. EYFW is the guarantor of this work and, as such, had full access to all the data in the study and takes responsibility for the integrity of the data and the accuracy of the data analysis.

## FUNDING INFORMATION

Excellent Young Scientists Fund, National Natural Science Foundation of China (Principal Investigator: Eric Yuk Fai Wan; Ref No. 82222902). The funders have no role in the study design, data collection, data analysis, interpretation, and report drafting. The corresponding authors had full access to all the data in the study and took final responsibility for the decision to submit for publication.

## CONFLICT OF INTEREST STATEMENT

EYFW has received research grants from the Health Bureau, the Hong Kong Research Grants Council, Narcotics Division, Security Bureau, Social Welfare Department, Labour and Welfare Bureau of the Government of the Hong Kong SAR and National Natural Science Foundation of China; serves on member of Core Team for Expert Group on Drug Registration of Pharmacy and Poisons Board, and is the director of Advance Data Analytics for Medical Science (ADAMS) Limited (HK). These are outside the submitted work. CLKL has received research grants from the Food and Health Bureau of the Government of the Hong Kong SAR, the Hong Kong Research Grant Council, the Hong Kong College of Family Physicians, and Kerry Group Kuok Foundation, outside the submitted work. The remaining authors have nothing to disclose.

## PEER REVIEW

The peer review history for this article is available at https://www.webofscience.com/api/gateway/wos/peer‐review/10.1111/dom.70061.

## Supporting information


Data S1

Data S2

## Data Availability

The electronic medical records used in the current study are provided by the Hospital Authority of Hong Kong and IQVIA. The data can be accessed upon request to the Hospital Authority of Hong Kong and IQVIA.

## References

[1] IDF . IDF Diabetes Atlas Ninth Edition 2019. updated 20 June 2021. Available from: www.diabetesatlas.org

[2] Fowler MJ . Microvascular and macrovascular complications of diabetes. Clinical Diabetes. 2008;26(2):77‐82. doi:10.2337/diaclin.26.2.77

[3] Eknoyan G , Nagy J . A history of diabetes mellitus or how a disease of the kidneys evolved into a kidney disease. Adv Chronic Kidney Dis. 2005;12(2):223‐229. doi:10.1053/j.ackd.2005.01.002 15822058

[4] Costacou T , Orchard TJ . Cumulative kidney complication risk by 50 years of type 1 diabetes: the effects of sex, age, and calendar year at onset. Diabetes Care. 2018;41(3):426‐433. doi:10.2337/dc17-1118 28931542 PMC5829956

[5] USRDS . USRDS Annual Data Report: Epidemiology of Kidney Disease in the United States. National Institutes of Health, National Institute of Diabetes and Digestive and Kidney Diseases; 2020.

[6] Mayer‐Davis EJ , Lawrence JM , Dabelea D , et al. Incidence trends of type 1 and type 2 diabetes among youths, 2002–2012. N Engl J Med. 2017;376(15):1419‐1429. doi:10.1056/NEJMoa1610187 28402773 PMC5592722

[7] Luk AOY , Ke C , Lau ESH , et al. Secular trends in incidence of type 1 and type 2 diabetes in Hong Kong: a retrospective cohort study. PLoS Med. 2020;17(2):e1003052. doi:10.1371/journal.pmed.1003052 [published Online First: 2020/02/23]. 32078650 PMC7032690

[8] Chan JCN , Lim LL , Wareham NJ , et al. The lancet commission on diabetes: using data to transform diabetes care and patient lives. Lancet. 2021;396(10267):2019‐2082. doi:10.1016/s0140-6736(20)32374-6 33189186

[9] Chan JCN , Lau ESH , Luk AOY , et al. Premature mortality and comorbidities in young‐onset diabetes: a 7‐year prospective analysis. Am J Med. 2014;127(7):616‐624. doi:10.1016/j.amjmed.2014.03.018 24680795

[10] Nanayakkara N , Curtis AJ , Heritier S , et al. Impact of age at type 2 diabetes mellitus diagnosis on mortality and vascular complications: systematic review and meta‐analyses. Diabetologia. 2021;64(2):275‐287. doi:10.1007/s00125-020-05319-w 33313987 PMC7801294

[11] Zheng L , Chen X , Luo T , et al. Early‐onset type 2 diabetes as a risk factor for end‐stage renal disease in patients with diabetic kidney disease. Prev Chronic Dis. 2020;17:17. doi:10.5888/pcd17.200076 PMC7367068 32614772

[12] Morton JI , Liew D , McDonald SP , Shaw JE , Magliano DJ . The association between age of onset of type 2 diabetes and the long‐term risk of end‐stage kidney disease: a National Registry Study. Diabetes Care. 2020;43(8):1788‐1795. doi:10.2337/dc20-0352 32540924

[13] Wu H , Lau ESH , Yang A , et al. Young age at diabetes diagnosis amplifies the effect of diabetes duration on risk of chronic kidney disease: a prospective cohort study. Diabetologia. 2021;64:1990‐2000. doi:10.1007/s00125-021-05494-4 34121143

[14] Shin J‐A , Lee J‐H , Lim S‐Y , et al. Metabolic syndrome as a predictor of type 2 diabetes, and its clinical interpretations and usefulness. J Diabetes Investig. 2013;4(4):334‐343. doi:10.1111/jdi.12075 [published Online First: 2013/05/28]. PMC4020225 24843675

[15] Zhang X , Lerman LO . The metabolic syndrome and chronic kidney disease. Transl Res. 2017;183:14‐25. doi:10.1016/j.trsl.2016.12.004 28025032 PMC5393937

[16] UK EMR . IQVIA Medical Research Data. 2020 [Available from: https://www.iqvia.com/library/fact‐sheets/uk‐emr‐iqvia‐medical‐research‐data accessed November 2021]

[17] Raman SR , Man KK , Bahmanyar S , et al. Trends in attention‐deficit hyperactivity disorder medication use: a retrospective observational study using population‐based databases. Lancet Psychiatry. 2018;5(10):824‐835. 30220514 10.1016/S2215-0366(18)30293-1

[18] Brauer R , Alfageh B , Blais JE , et al. Psychotropic medicine consumption in 65 countries and regions, 2008–19: a longitudinal study. Lancet Psychiatry. 2021;8(12):1071‐1082. 34801129 10.1016/S2215-0366(21)00292-3 PMC9766760

[19] Ma T‐T , Wong IC , Whittlesea C , et al. Impact of multiple cardiovascular medications on mortality after an incidence of ischemic stroke or transient ischemic attack. BMC Med. 2021;19(1):1‐11. 33530992 10.1186/s12916-021-01900-1 PMC7856718

[20] Blak BT , Thompson M , Dattani H , Blak B , Bourke A . Generalisability of the health improvement network (THIN) database: demographics, chronic disease prevalence and mortality rates. Inform Prim Care. 2011;19(4):251‐255. doi:10.14236/jhi.v19i4.820 [published Online First: 2011/01/01]. 22828580

[21] Statistics OfN . Census: Key Statistics and Quick Statistics for Local Authorities in the United Kingdom 2013. 2011 Available from: https://www.ons.gov.uk/peoplepopulationandcommunity/populationandmigration/populationestimates/bulletins/keystatisticsandquickstatisticsforlocalauthoritiesintheunitedkingdom/2013‐10‐11 accessed Mar 28 2022

[22] Authority H . Clusters, Hospitals & Institutions. 2023 [cited 2023 12/04]. Available from: https://www.ha.org.hk/visitor/ha_visitor_index.asp?Content_ID=10036&Dimension=100 accessed 12/04/2023.

[23] Cheung NT , Fung V , Wong WN , et al. Principles‐based medical informatics for success—how Hong Kong built one of the world's largest integrated longitudinal electronic patient records. Stud Health Technol Inform. 2007;129(Pt 1):307‐310. 17911728

[24] Wong AY , Root A , Douglas IJ , et al. Cardiovascular outcomes associated with use of clarithromycin: population based study. BMJ. 2016;352:h6926. doi:10.1136/bmj.h6926 26768836

[25] Wan EYF , Chin WY , Yu EYT , et al. The impact of cardiovascular disease and chronic kidney disease on life expectancy and direct medical cost in a 10‐year diabetes cohort study. Diabetes Care. 2020;43(8):1750‐1758. doi:10.2337/dc19-2137 32457057

[26] Wan EYF , Yu EYT , Chin WY , Fong DYT , Choi EPH , Lam CLK . Association of blood pressure and risk of cardiovascular and chronic kidney disease in Hong Kong hypertensive patients. Hypertension. 2019;74(2):331‐340. doi:10.1161/HYPERTENSIONAHA.119.13123 31230539 PMC6635057

[27] Census and Statistics Department, Hong Kong Special Administrative Region . Hong Kong 2016 Population by‐Census Summary Results. Hong Kong Government Press; 2017.

[28] Inker LA , Eneanya ND , Coresh J , et al. New creatinine‐ and cystatin C‐based equations to estimate GFR without race. N Engl J Med. 2021;385(19):1737‐1749. doi:10.1056/NEJMoa2102953 34554658 PMC8822996

[29] Matsushita K , Chen J , Sang Y , et al. Risk of end‐stage renal disease in Japanese patients with chronic kidney disease increases proportionately to decline in estimated glomerular filtration rate. Kidney Int. 2016;90(5):1109‐1114. doi:10.1016/j.kint.2016.08.003 27666758

[30] Levey AS , Inker LA , Matsushita K , et al. GFR decline as an end point for clinical trials in CKD: a scientific workshop sponsored by the National Kidney Foundation and the US Food and Drug Administration. Am J Kidney Dis. 2014;64(6):821‐835. doi:10.1053/j.ajkd.2014.07.030 25441437

[31] Coresh J , Turin TC , Matsushita K , et al. Decline in estimated glomerular filtration rate and subsequent risk of end‐stage renal disease and mortality. JAMA. 2014;311(24):2518‐2531. doi:10.1001/jama.2014.6634 24892770 PMC4172342

[32] Gerstein HC , Colhoun HM , Dagenais GR , et al. Dulaglutide and cardiovascular outcomes in type 2 diabetes (REWIND): a double‐blind, randomised placebo‐controlled trial. Lancet. 2019;394(10193):121‐130. doi:10.1016/s0140-6736(19)31149-3 31189511

[33] Iwagami M , Caplin B , Smeeth L , et al. Clinical Codelist—Read Codes for Coronary Heart Disease. London School of Hygiene & Tropical Medicine; 2018.

[34] Matthews A , Bhaskaran K , Hygiene LS , et al. Clinical Code List—Stroke. London School of Hygiene & Tropical Medicine; 2018.

[35] Iwagami M , Tomlinson LA , Hygiene LS , et al. Clinical Codelist—Heart Failure Read Codes. London School of Hygiene & Tropical Medicine; 2020.

[36] Linden A . Combining propensity score‐based stratification and weighting to improve causal inference in the evaluation of health care interventions. J Eval Clin Pract. 2014;20(6):1065‐1071. doi:10.1111/jep.12254 25266868

[37] Hong G . Marginal mean weighting through stratification: adjustment for selection bias in multilevel data. J Educ Behav Stat. 2010;35(5):499‐531.

[38] Hong G . Marginal mean weighting through stratification: a generalized method for evaluating multivalued and multiple treatments with nonexperimental data. Psychol Methods. 2012;17(1):44‐60. doi:10.1037/a0024918 21843003

[39] Austin PC . Some methods of propensity‐score matching had superior performance to others: results of an empirical investigation and Monte Carlo simulations. Biom J. 2009;51(1):171‐184. doi:10.1002/bimj.200810488 19197955

[40] James Gareth DW , Hastie T , Tibshirani R . An Introduction to Statistical Learning: with Applications in R. Springer; 2013.

[41] Fine JP , Gray RJ . A proportional hazards model for the subdistribution of a competing risk. J Am Stat Assoc. 1999;94(446):496‐509. doi:10.2307/2670170

[42] Kong AP , Xu G , Brown N , So WY , Ma RC , Chan JC . Diabetes and its comorbidities—where east meets west. Nat Rev Endocrinol. 2013;9(9):537‐547. 23712250 10.1038/nrendo.2013.102

[43] Ma RC , Chan JC . Type 2 diabetes in east Asians: similarities and differences with populations in Europe and the United States. Ann N Y Acad Sci. 2013;1281(1):64‐91. 23551121 10.1111/nyas.12098 PMC3708105

[44] Liu J , Lim S , Yeoh L , et al. Ethnic disparities in risk of cardiovascular disease, end‐stage renal disease and all‐cause mortality: a prospective study among Asian people with type 2 diabetes. Diabet Med. 2016;33(3):332‐339. 26514089 10.1111/dme.13020

[45] Mooyaart A , Valk E , Van Es L , et al. Genetic associations in diabetic nephropathy: a meta‐analysis. Diabetologia. 2011;54(3):544‐553. 21127830 10.1007/s00125-010-1996-1 PMC3034040

[46] Piko P , Werissa NA , Fiatal S , Sandor J , Adany R . Impact of genetic factors on the age of onset for type 2 diabetes mellitus in addition to the conventional risk factors. J Pers Med. 2020;11(1):6. doi:10.3390/jpm11010006 33375163 PMC7822179

[47] Ahlqvist E , van Zuydam NR , Groop LC , McCarthy MI . The genetics of diabetic complications. Nat Rev Nephrol. 2015;11(5):277‐287. doi:10.1038/nrneph.2015.37 25825086

[48] Cole JB , Florez JC . Genetics of diabetes mellitus and diabetes complications. Nat Rev Nephrol. 2020;16(7):377‐390. doi:10.1038/s41581-020-0278-5 32398868 PMC9639302

[49] Kong X , Xing X , Zhang X , Hong J , Yang W . Early‐onset type 2 diabetes is associated with genetic variants of *β*‐cell function in the Chinese Han population. Diabetes Metab Res Rev. 2020;36(2):e3214. doi:10.1002/dmrr.3214 31465628

[50] van Zuydam NR , Ahlqvist E , Sandholm N , et al. A genome‐wide association study of diabetic kidney disease in subjects with type 2 diabetes. Diabetes. 2018;67(7):1414‐1427. doi:10.2337/db17-0914 29703844 PMC6014557

[51] Taira M , Imamura M , Takahashi A , et al. A variant within the FTO confers susceptibility to diabetic nephropathy in Japanese patients with type 2 diabetes. PLoS One. 2018;13(12):e0208654. doi:10.1371/journal.pone.0208654 30566433 PMC6300288

[52] Pattaro C , Teumer A , Gorski M , et al. Genetic associations at 53 loci highlight cell types and biological pathways relevant for kidney function. Nat Commun. 2016;7:10023. doi:10.1038/ncomms10023 26831199 PMC4735748

[53] Quah JHM , Liu YP , Luo N , How CH , Tay EG . Younger adult type 2 diabetic patients have poorer glycaemic control: a cross‐sectional study in a primary care setting in Singapore. BMC Endocr Disord. 2013;13:18. doi:10.1186/1472-6823-13-18 23725198 PMC3674913

[54] Patel P , Macerollo A . Diabetes mellitus: diagnosis and screening. Am Fam Physician. 2010;81(7):863‐870. 20353144

[55] Hiller J , Schatz K , Drexler H . Gender influence on health and risk behavior in primary prevention: a systematic review. J Public Health. 2017;25(4):339‐349. doi:10.1007/s10389-017-0798-z PMC7088168 32215245

[56] Berkowitz SA , Meigs JB , Wexler DJ . Age at type 2 diabetes onset and glycaemic control: results from the National Health and nutrition examination survey (NHANES) 2005‐2010. Diabetologia. 2013;56(12):2593‐2600. doi:10.1007/s00125-013-3036-4 23995472 PMC3818392

[57] Wright AK , Welsh P , Gill JMR , et al. Age‐, sex‐ and ethnicity‐related differences in body weight, blood pressure, HbA(1c) and lipid levels at the diagnosis of type 2 diabetes relative to people without diabetes. Diabetologia. 2020;63(8):1542‐1553. doi:10.1007/s00125-020-05169-6 32435821 PMC7351865

[58] Wu T , Ding L , Andoh V , Zhang J , Chen L . The mechanism of hyperglycemia‐induced renal cell injury in diabetic nephropathy disease: an update. Life. 2023;13(2):539. doi:10.3390/life13020539 [published Online First: 20230215]. 36836895 PMC9967500

[59] Fu H , Liu S , Bastacky SI , Wang X , Tian XJ , Zhou D . Diabetic kidney diseases revisited: a new perspective for a new era. Mol Metab. 2019;30:250‐263. doi:10.1016/j.molmet.2019.10.005 31767176 PMC6838932

[60] Alicic RZ , Rooney MT , Tuttle KR . Diabetic kidney disease: challenges, Progress, and possibilities. Clin J Am Soc Nephrol. 2017;12(12):2032‐2045. doi:10.2215/cjn.11491116 28522654 PMC5718284

[61] Effects of treatment of impaired glucose tolerance or recently diagnosed type 2 diabetes with metformin alone or in combination with insulin glargine on *β*‐Cell function: comparison of responses in youth and adults. Diabetes. 2019;68(8):1670‐1680. doi:10.2337/db19-0299 31178433 PMC6692818

[62] Ke C , Stukel TA , Shah BR , et al. Age at diagnosis, glycemic trajectories, and responses to oral glucose‐lowering drugs in type 2 diabetes in Hong Kong: a population‐based observational study. PLoS Med. 2020;17(9):e1003316. doi:10.1371/journal.pmed.1003316 32946450 PMC7500681

