# Supplementary material for: Impact of onset age of type 2 diabetes mellitus on risk of renal complications compared to age‐matched non‐diabetic patients: Two cohort studies in the United Kingdom and Hong Kong
Source: Diabetes Obes Metab. 2025 Sep 1;27(11):6577–93. doi: 10.1111/dom.70061 (PMC12515758; doi:10.1111/dom.70061)
Supplement: Supplementary file 1 — Data S1 [file DOM-27-6577-s002.docx]

**Supplementary table 1. Read code list for identifying type-2 diabetes, chronic kidney disease, end-stage renal disease in THIN dataset**

| **Read code** | **Definition** |
| --- | --- |
| ***Type 2 diabetes mellitus*** | |
| C10F.00 | Type 2 diabetes mellitus |
| C10FJ00 | Insulin treated Type 2 diabetes mellitus |
| C10FM00 | Type 2 diabetes mellitus with persistent microalbuminuria |
| C10FL00 | Type 2 diabetes mellitus with persistent proteinuria |
| C10F.11 | Type II diabetes mellitus |
| C10FC00 | Type 2 diabetes mellitus with nephropathy |
| C10F600 | Type 2 diabetes mellitus with retinopathy |
| C10F700 | Type 2 diabetes mellitus - poor control |
| C10F900 | Type 2 diabetes mellitus without complication |
| C10FN00 | Type 2 diabetes mellitus with ketoacidosis |
| C10FK00 | Hyperosmolar non-ketotic state in type 2 diabetes mellitus |
| C10FB00 | Type 2 diabetes mellitus with polyneuropathy |
| C10F911 | Type II diabetes mellitus without complication |
| C10FQ00 | Type 2 diabetes mellitus with exudative maculopathy |
| C10F000 | Type 2 diabetes mellitus with renal complications |
| C10FH00 | Type 2 diabetes mellitus with neuropathic arthropathy |
| C10F200 | Type 2 diabetes mellitus with neurological complications |
| C10FE00 | Type 2 diabetes mellitus with diabetic cataract |
| C10F400 | Type 2 diabetes mellitus with ulcer |
| C10FR00 | Type 2 diabetes mellitus with gastroparesis |
| C10FJ11 | Insulin treated Type II diabetes mellitus |
| C10F711 | Type II diabetes mellitus - poor control |
| C10FD00 | Type 2 diabetes mellitus with hypoglycaemic coma |
| C10FA00 | Type 2 diabetes mellitus with mononeuropathy |
| C10FL11 | Type II diabetes mellitus with persistent proteinuria |
| C10F100 | Type 2 diabetes mellitus with ophthalmic complications |
| C10F500 | Type 2 diabetes mellitus with gangrene |
| C10FF00 | Type 2 diabetes mellitus with peripheral angiopathy |
| C10FG00 | Type 2 diabetes mellitus with arthropathy |
| C10F611 | Type II diabetes mellitus with retinopathy |
| C10F300 | Type 2 diabetes mellitus with multiple complications |
| C10FP00 | Type 2 diabetes mellitus with ketoacidotic coma |
| C10FA11 | Type II diabetes mellitus with mononeuropathy |
| C10F311 | Type II diabetes mellitus with multiple complications |
| C10FE11 | Type II diabetes mellitus with diabetic cataract |
| C10FD11 | Type II diabetes mellitus with hypoglycaemic coma |
| C10FC11 | Type II diabetes mellitus with nephropathy |
| C10F411 | Type II diabetes mellitus with ulcer |
| C10FG11 | Type II diabetes mellitus with arthropathy |
| C10FB11 | Type II diabetes mellitus with polyneuropathy |
| C10F011 | Type II diabetes mellitus with renal complications |
| C10FM11 | Type II diabetes mellitus with persistent microalbuminuria |
| C10F111 | Type II diabetes mellitus with ophthalmic complications |
| C10F211 | Type II diabetes mellitus with neurological complications |
| C10FF11 | Type II diabetes mellitus with peripheral angiopathy |
| C10F511 | Type II diabetes mellitus with gangrene |
| C109.00 | Non-insulin dependent diabetes mellitus |
| C109.12 | Type 2 diabetes mellitus |
| C109.11 | NIDDM - Non-insulin dependent diabetes mellitus |
| C109.13 | Type II diabetes mellitus |
| C109J00 | Insulin treated Type 2 diabetes mellitus |
| C109700 | Non-insulin dependent diabetes mellitus - poor control |
| C109900 | Non-insulin-dependent diabetes mellitus without complication |
| C109400 | Non-insulin dependent diabetes mellitus with ulcer |
| C109600 | Non-insulin-dependent diabetes mellitus with retinopathy |
| C109C00 | Non-insulin dependent diabetes mellitus with nephropathy |
| C10z100 | Diabetes mellitus, adult onset, + unspecified complication |
| L180600 | Pre-existing diabetes mellitus, non-insulin-dependent |
| C109J12 | Insulin treated Type II diabetes mellitus |
| C109711 | Type II diabetes mellitus - poor control |
| C109712 | Type 2 diabetes mellitus - poor control |
| C109K00 | Hyperosmolar non-ketotic state in type 2 diabetes mellitus |
| C107400 | NIDDM with peripheral circulatory disorder |
| C109611 | Type II diabetes mellitus with retinopathy |
| C109411 | Type II diabetes mellitus with ulcer |
| C109J11 | Insulin treated non-insulin dependent diabetes mellitus |
| C109100 | Non-insulin-dependent diabetes mellitus with ophthalm comps |
| C109000 | Non-insulin-dependent diabetes mellitus with renal comps |
| C109612 | Type 2 diabetes mellitus with retinopathy |
| C109200 | Non-insulin-dependent diabetes mellitus with neuro comps |
| C109212 | Type 2 diabetes mellitus with neurological complications |
| C109412 | Type 2 diabetes mellitus with ulcer |
| C109E00 | Non-insulin depend diabetes mellitus with diabetic cataract |
| C109B00 | Non-insulin dependent diabetes mellitus with polyneuropathy |
| C109C12 | Type 2 diabetes mellitus with nephropathy |
| C109E11 | Type II diabetes mellitus with diabetic cataract |
| C109H12 | Type 2 diabetes mellitus with neuropathic arthropathy |
| C109500 | Non-insulin dependent diabetes mellitus with gangrene |
| C109H00 | Non-insulin dependent d m with neuropathic arthropathy |
| C109D00 | Non-insulin dependent diabetes mellitus with hypoglyca coma |
| C109011 | Type II diabetes mellitus with renal complications |
| C109300 | Non-insulin-dependent diabetes mellitus with multiple comps |
| C109012 | Type 2 diabetes mellitus with renal complications |
| C109E12 | Type 2 diabetes mellitus with diabetic cataract |
| C109H11 | Type II diabetes mellitus with neuropathic arthropathy |
| C109511 | Type II diabetes mellitus with gangrene |
| C10y100 | Diabetes mellitus, adult, + other specified manifestation |
| C109F00 | Non-insulin-dependent d m with peripheral angiopath |
| C109D12 | Type 2 diabetes mellitus with hypoglycaemic coma |
| C109G00 | Non-insulin dependent diabetes mellitus with arthropathy |
| C109512 | Type 2 diabetes mellitus with gangrene |
| C109C11 | Type II diabetes mellitus with nephropathy |
| C109F11 | Type II diabetes mellitus with peripheral angiopathy |
| C109B11 | Type II diabetes mellitus with polyneuropathy |
| C109211 | Type II diabetes mellitus with neurological complications |
| C109D11 | Type II diabetes mellitus with hypoglycaemic coma |
| C109111 | Type II diabetes mellitus with ophthalmic complications |
| C109F12 | Type 2 diabetes mellitus with peripheral angiopathy |
| C109A00 | Non-insulin dependent diabetes mellitus with mononeuropathy |
| C109G11 | Type II diabetes mellitus with arthropathy |
| C109G12 | Type 2 diabetes mellitus with arthropathy |
| C109A11 | Type II diabetes mellitus with mononeuropathy |
| C109112 | Type 2 diabetes mellitus with ophthalmic complications |
| 66A3.00 | Diabetic on diet only |
| 66A4.00 | Diabetic on oral treatment |
| ***Chronic kidney disease*** | |
| 1Z12.00 | Chronic kidney disease stage 3 |
| 1Z15.00 | Chronic kidney disease stage 3A |
| 1Z16.00 | Chronic kidney disease stage 3B |
| 1Z1B.11 | CKD stage 3 with proteinuria |
| 1Z1C.00 | Chronic kidney disease stage 3 without proteinuria |
| 1Z1C.11 | CKD stage 3 without proteinuria |
| 1Z1D.00 | Chronic kidney disease stage 3A with proteinuria |
| 1Z1D.11 | CKD stage 3A with proteinuria |
| 1Z1E.00 | Chronic kidney disease stage 3A without proteinuria |
| 1Z1E.11 | CKD stage 3A without proteinuria |
| 1Z1F.00 | Chronic kidney disease stage 3B with proteinuria |
| 1Z1F.11 | CKD stage 3B with proteinuria |
| 1Z1G.00 | Chronic kidney disease stage 3B without proteinuria |
| 1Z1G.11 | CKD stage 3B without proteinuria |
| 1Z1T.00 | CKD with GFR category G3a & albuminuria category A1 |
| 1Z1V.00 | CKD with GFR category G3a & albuminuria category A2 |
| 1Z1W.00 | CKD with GFR category G3a & albuminuria category A3 |
| 1Z1X.00 | CKD with GFR category G3b & albuminuria category A1 |
| 1Z1Y.00 | CKD with GFR category G3b & albuminuria category A2 |
| 1Z1Z.00 | CKD with GFR category G3b & albuminuria category A3 |
| K053.00 | Chronic kidney disease stage 3 |
| 1Z13.00 | Chronic kidney disease stage 4 |
| 1Z14.00 | Chronic kidney disease stage 5 |
| 1Z1a.00 | CKD with GFR category G4 & albuminuria category A1 |
| 1Z1b.00 | CKD with GFR category G4 & albuminuria category A2 |
| 1Z1c.00 | CKD with GFR category G4 & albuminuria category A3 |
| 1Z1d.00 | CKD with GFR category G5 & albuminuria category A1 |
| 1Z1e.00 | CKD with GFR category G5 & albuminuria category A2 |
| 1Z1f.00 | CKD with GFR category G5 & albuminuria category A3 |
| 1Z1H.00 | Chronic kidney disease stage 4 with proteinuria |
| 1Z1H.11 | CKD stage 4 with proteinuria |
| 1Z1J.00 | Chronic kidney disease stage 4 without proteinuria |
| 1Z1J.11 | CKD stage 4 without proteinuria |
| 1Z1K.00 | Chronic kidney disease stage 5 with proteinuria |
| 1Z1K.11 | CKD stage 5 with proteinuria |
| 1Z1L.00 | Chronic kidney disease stage 5 without proteinuria |
| 1Z1L.11 | CKD stage 5 without proteinuria |
| D215.00 | Anaemia secondary to renal failure |
| D215000 | Anaemia secondary to chronic renal failure |
| G222.00 | Hypertensive renal disease with renal failure |
| G233.00 | Hypertensive heart and renal disease with renal failure |
| G234.00 | Hyperten heart&renal dis+both(congestv)heart and renal fail |
| G752000 | Goodpasture's syndrome |
| G752100 | Goodpasture's disease |
| K01..00 | Nephrotic syndrome |
| K010.00 | Nephrotic syndrome with proliferative glomerulonephritis |
| K011.00 | Nephrotic syndrome with membranous glomerulonephritis |
| K012.00 | Nephrotic syndrome+membranoproliferative glomerulonephritis |
| K013.00 | Nephrotic syndrome with minimal change glomerulonephritis |
| K013.11 | Lipoid nephrosis |
| K013.12 | Steroid sensitive nephrotic syndrome |
| K014.00 | Nephrotic syndrome minor glomerular abnormality |
| K015.00 | Nephrotic syndrome focal and segmental glomerular lesions |
| K016.00 | Nephrotic syndrome diffuse membranous glomerulonephritis |
| K017.00 | Nephrotic syn difus mesangial prolifertiv glomerulonephritis |
| K018.00 | Nephrotic syn difus endocapilary proliftv glomerulonephritis |
| K019.00 | Nephrotic syn diffuse mesangiocapillary glomerulonephritis |
| K01A.00 | Nephrotic syndrome dense deposit disease |
| K01B.00 | Nephrotic syndrome diffuse crescentic glomerulonephritis |
| K01w.00 | Congenital nephrotic syndrome |
| K01w000 | Finnish nephrosis syndrome |
| K01w100 | Drash syndrome |
| K01w111 | Nephrotic syndrome with pseudohermaphroditism |
| K01w112 | Wilms' tumour + nephrotic syndrome + pseudohermaphroditism |
| K01w200 | Congenital nephrotic syndrome with focal glomerulosclerosis |
| K01wz00 | Congenital nephrotic syndrome NOS |
| K01x.00 | Nephrotic syndrome in diseases EC |
| K01x000 | Nephrotic syndrome in amyloidosis |
| K01x100 | Nephrotic syndrome in diabetes mellitus |
| K01x200 | Nephrotic syndrome in malaria |
| K01x300 | Nephrotic syndrome in polyarteritis nodosa |
| K01x400 | Nephrotic syndrome in systemic lupus erythematosus |
| K01y.00 | Nephrotic syndrome with other pathological kidney lesions |
| K01z.00 | Nephrotic syndrome NOS |
| K023.00 | Chronic rapidly progressive glomerulonephritis |
| K033.00 | Rapidly progressive nephritis unspecified |
| K05..00 | Chronic renal failure |
| K05..12 | End stage renal failure |
| K050.00 | End stage renal failure |
| K054.00 | Chronic kidney disease stage 4 |
| K055.00 | Chronic kidney disease stage 5 |
| K0A0700 | Acute nephrotic syndrm diffuse crescentic glomerulonephritis |
| K0A1.00 | Rapidly progressive nephritic syndrome |
| K0A1100 | Rapid progres nephritic syn focal+segmental glomerulr lesion |
| K0A1200 | Rapid progres neph syn diffuse membranous glomerulonephritis |
| K0A1300 | Rpd prog neph syn df mesangial prolifratv glomerulonephritis |
| K0A1400 | Rapid progres neph syn df endocapilary prolifv glomnephritis |
| K0A1600 | Rapid progressive nephritic syndrome dense deposit disease |
| K0A1700 | Rapid progres nephritic syn df crescentic glomerulonephritis |
| K0A8.00 | Rapidly progressive glomerulonephritis |
| K0B5.00 | Renal tubulo-interstitial disordrs in transplant rejectn |
| K0D..00 | End-stage renal disease |
| K0E..00 | Acute-on-chronic renal failure |
| Kyu2100 | [X]Other chronic renal failure |
| ***End-stage renal disease*** | |
| 7L1A100 | peritoneal dialysis |
| G760.00 | acquired arteriovenous fistula |
| 7L1A200 | haemodialysis nec |
| 7B00.00 | transplantation of kidney |
| 7A60100 | creation of arteriovenous fistula nec |
| 7B00z00 | transplantation of kidney nos |
| ZV42000 | [v]kidney transplanted |
| K050.00 | end stage renal failure |
| 7L1B000 | insertion of ambulatory peritoneal dialysis catheter |
| K0D..00 | end-stage renal disease |
| 7A61400 | ligation of acquired arteriovenous fistula |
| SP08300 | kidney transplant failure and rejection |
| 7B00100 | transplantation of kidney from live donor |
| 7L1A.11 | dialysis for renal failure |
| 1Z14.00 | chronic kidney disease stage 5 |
| 8L50.00 | renal transplant planned |
| TB00111 | renal transplant with complication, without blame |
| 7A61100 | repair of acquired arteriovenous fistula |
| 7L1A000 | renal dialysis |
| 14V2.00 | h/o: renal dialysis |
| ZV45100 | [v]renal dialysis status |
| 7L1B100 | removal of ambulatory peritoneal dialysis catheter |
| 7A60.00 | arteriovenous shunt |
| 7B00200 | transplantation of kidney from cadaver |
| 7A60112 | creation of brachial-cephalic fistula |
| 7B06300 | exploration of renal transplant |
| TB11.00 | kidney dialysis with complication, without blame |
| 7A60111 | creation of radial-cephalic fistula |
| 7L1C000 | insertion of temporary peritoneal dialysis catheter |
| 7L1A500 | continuous ambulatory peritoneal dialysis |
| 7A60300 | removal of infected arteriovenous shunt |
| 7L1A.00 | compensation for renal failure |
| TA22.00 | failure of sterile precautions during perfusion |
| 7L1B.11 | placement ambulatory dialysis apparatus - compens renal fail |
| 14V2.11 | h/o: kidney dialysis |
| ZV56y11 | [v]aftercare involving peritoneal dialysis |
| ZV56011 | [v]aftercare involving renal dialysis nos |
| SP05613 | [x] peritoneal dialysis associated peritonitis |
| 7L1Ay00 | other specified compensation for renal failure |
| K0B5.00 | renal tubulo-interstitial disordrs in transplant rejectn |
| 7B01500 | transplant nephrectomy |
| SP01500 | mechanical complication of dialysis catheter |
| SP01700 | mechanical complication of arterio-venous surgical fistula |
| 14S2.00 | h/o: kidney recipient |
| ZV56100 | [v]preparatory care for dialysis |
| K05..12 | end stage renal failure |
| U612200 | [x]failure sterile precautions dur kidney dialys/other perf |
| TB00100 | kidney transplant with complication, without blame |
| 7L1B.00 | placement ambulatory apparatus compensation renal failure |
| 7A60z00 | arteriovenous shunt nos |
| 7L1By00 | placement ambulatory apparatus- compensate renal failure os |
| SP07G00 | stenosis of arteriovenous dialysis fistula |
| 7A60600 | creation of graft fistula for dialysis |
| Z919.00 | care of haemodialysis equipment |
| Z919300 | reversing haemodialysis lines |
| ZV56.00 | [v]aftercare involving intermittent dialysis |
| 7A60000 | insertion of arteriovenous prosthesis |
| 7A60200 | attention to arteriovenous shunt |
| 7A60500 | thrombectomy of arteriovenous fistula |
| ZV56y00 | [v]other specified aftercare involving intermittent dialysis |
| Z91A.00 | peritoneal dialysis bag procedure |
| 7L1Az00 | compensation for renal failure nos |
| 7L1A600 | peritoneal dialysis nec |
| 7L1Cz00 | placement other apparatus- compensate for renal failure nos |
| 7A60y00 | other specified arteriovenous shunt |
| 7B00111 | allotransplantation of kidney from live donor |
| TB11.11 | renal dialysis with complication, without blame |
| TA22000 | failure of sterile precautions during kidney dialysis |
| ZVu3G00 | [x]other dialysis |
| SP08011 | det.ren.func.after ren.transpl |
| 7B00y00 | other specified transplantation of kidney |
| 7L1A300 | haemofiltration |
| 7B01511 | excision of rejected transplanted kidney |
| Z919100 | priming haemodialysis lines |
| Z1A2.00 | haemodialysis training |
| 7L1C.00 | placement other apparatus for compensation for renal failure |
| 4N2..00 | dialysis fluid glucose level |
| 7L1A400 | automated peritoneal dialysis |
| 9Ot5.00 | predicted stage chronic kidney disease |
| 7B00300 | allotransplantation of kidney from cadaver heart-beating |
| 7B0F100 | pre-transplantation of kidney work-up, recipient |
| 7B0F.00 | interventions associated with transplantation of kidney |
| 1Z1L.00 | chronic kidney disease stage 5 without proteinuria |
| 1Z1K.00 | chronic kidney disease stage 5 with proteinuria |
| 7A60400 | banding of arteriovenous fistula |
| 7B00400 | allotransplantation kidney from cadaver heart non-beating |
| TA02000 | accid cut,puncture,perf,h'ge - kidney dialysis |
| 7A61900 | ligation of arteriovenous dialysis fistula |
| 1Z1L.11 | ckd stage 5 without proteinuria |
| 7B00211 | allotransplantation of kidney from cadaver |
| 4I29.00 | peritoneal dialysis sample |
| 1Z1K.11 | ckd stage 5 with proteinuria |
| ZV56000 | [v]aftercare involving extracorporeal dialysis |
| 7A61111 | ligation of acquired arteriovenous fistula |
| Kyu1C00 | [x]renal tubulo-interstitial disorders/transplant rejection |
| SP06B00 | continuous ambulatory peritoneal dialysis associated perit |
| 4N0..00 | dialysis fluid urea level |
| 7L1A011 | thomas intravascular shunt for dialysis |
| Z1A..00 | dialysis training |
| 7B0F300 | post-transplantation of kidney examination, recipient |
| 9b8K.00 | transplantation surgery |
| 7B0Fz00 | interventions associated with transplantation of kidney nos |
| 7B0Fy00 | os interventions associated with transplantation of kidney |
| 7L1B200 | flushing of peritoneal dialysis catheter |
| Z1A1.00 | peritoneal dialysis training |
| K055.00 | chronic kidney disease stage 5 |
| 7B00212 | cadaveric renal transplant |
| SP0G.00 | anaphylactoid reaction due to haemodialysis |
| SP08N00 | unexplained episode of renal transplant dysfunction |
| G72D200 | aneurysm of anastomotic site of dialysis av fistula |
| G72C.00 | ruptured aneurysm of dialysis vascular access |
| 7B00600 | xenograft renal transplant |
| SP08R00 | renal transplant rejection |
| SP08J00 | chronic rejection of renal transplant |
| Gy21.00 | thrombosis of dialysis arteriovenous fistula |
| SP08W00 | vascular complication of renal transplant |
| Gy51.00 | haemorrhage of dialysis arteriovenous fistula |
| Gy31.00 | occlusion of dialysis arteriovenous fistula |
| 67P4100 | discussion about kidney transplantation |
| G72D.00 | aneurysm of dialysis arteriovenous fistula |
| SP07N00 | arteriovenous fistula thrombosis |
| G72D100 | aneurysm of needle site of dialysis arteriovenous fistula |
| Gy41.00 | infection of dialysis arteriovenous fistula |
| 7A61A00 | ligation of arteriovenous dialysis graft |
| Gy1..00 | stenosis of dialysis vascular access |
| SP08T00 | urological complication of renal transplant |
| SP0E.00 | disorders associated with peritoneal dialysis |
| 7L1Cy00 | placement other apparatus- compensate for renal failure os |
| Gy3..00 | occlusion of dialysis vascular access |
| Gy40.00 | infection of dialysis arteriovenous graft |
| Gy60.00 | rupture of dialysis arteriovenous graft |

**Supplementary table 2a. Baseline characteristics of subjects in different age groups before weighting**

|  | Age 18-39 (N=2,443,807) | | | Age 40-49 (N=965,386) | | | Age 50-59 (N=711,892) | | |
| --- | --- | --- | --- | --- | --- | --- | --- | --- | --- |
|  | No T2DM (N=2,420,328) | New onset T2DM (N=23,479) | SMD | No T2DM (N=945,033) | New onset T2DM (N=20,353) | SMD | No T2DM (N=683,301) | New onset T2DM (N=28,591) | SMD |
| ***United Kingdom*** |  | | |  | | |  | | |
| Male | 1,152,788 (47.6%) | 5,029 (21.4%) | 0.57 | 486,082 (51.4%) | 12,011 (59.0%) | 0.15 | 344,298 (50.4%) | 17,735 (62.0%) | 0.24 |
| Age, years | 28.3 (6.2) | 31.1 (5.5) | 0.44 | 44.3 (2.8) | 44.9 (2.9) | 0.22 | 54.3 (2.9) | 54.6 (2.9) | 0.11 |
| Current smoker | 588,797 (24.3%) | 4,448 (18.9%) | 0.13 | 215,867 (22.8%) | 4,608 (22.6%) | 0.00 | 138,725 (20.3%) | 5,683 (19.9%) | 0.01 |
| Obesity | 203,251 (8.4%) | 11,290 (48.1%) | 0.98 | 149,408 (15.8%) | 12,163 (59.8%) | 1.02 | 121,390 (17.8%) | 16,761 (58.6%) | 0.93 |
| Atrial fibrillation | 974 (0.0%) | 25 (0.1%) | 0.02 | 1,914 (0.2%) | 100 (0.5%) | 0.05 | 3,945 (0.6%) | 402 (1.4%) | 0.08 |
| Peripheral vascular disease | 112 (0.0%) | 5 (0.0%) | 0.01 | 450 (0.0%) | 47 (0.2%) | 0.05 | 2,041 (0.3%) | 271 (0.9%) | 0.08 |
| Amputation | 1,832 (0.1%) | 35 (0.1%) | 0.02 | 1,517 (0.2%) | 57 (0.3%) | 0.03 | 1,406 (0.2%) | 119 (0.4%) | 0.04 |
| Dementia | 20 (0.0%) | 0 (0.0%) | 0.00 | 75 (0.0%) | 3 (0.0%) | 0.01 | 365 (0.1%) | 19 (0.1%) | 0.01 |
| Chronic lung disease | 643 (0.0%) | 24 (0.1%) | 0.03 | 3,019 (0.3%) | 232 (1.1%) | 0.10 | 9,263 (1.4%) | 947 (3.3%) | 0.13 |
| Connective tissue disease | 6,917 (0.3%) | 151 (0.6%) | 0.05 | 7,455 (0.8%) | 265 (1.3%) | 0.05 | 9,476 (1.4%) | 559 (2.0%) | 0.04 |
| Peptic ulcer disease | 4,322 (0.2%) | 68 (0.3%) | 0.02 | 7,624 (0.8%) | 229 (1.1%) | 0.03 | 10,224 (1.5%) | 578 (2.0%) | 0.04 |
| Liver disease | 1,325 (0.1%) | 56 (0.2%) | 0.05 | 1,625 (0.2%) | 106 (0.5%) | 0.06 | 1,757 (0.3%) | 217 (0.8%) | 0.07 |
| Cardiovascular disease | 2,709 (0.1%) | 114 (0.5%) | 0.07 | 6,599 (0.7%) | 768 (3.8%) | 0.21 | 17,621 (2.6%) | 2,479 (8.7%) | 0.27 |
| Hemiplegia | 1,542 (0.1%) | 21 (0.1%) | 0.01 | 821 (0.1%) | 43 (0.2%) | 0.03 | 793 (0.1%) | 62 (0.2%) | 0.02 |
| Leukemia | 688 (0.0%) | 14 (0.1%) | 0.01 | 262 (0.0%) | 24 (0.1%) | 0.03 | 410 (0.1%) | 31 (0.1%) | 0.02 |
| Malignant lymphoma | 1,244 (0.1%) | 20 (0.1%) | 0.01 | 1,030 (0.1%) | 47 (0.2%) | 0.03 | 1,108 (0.2%) | 60 (0.2%) | 0.01 |
| Cancer | 5,999 (0.2%) | 112 (0.5%) | 0.04 | 8,916 (0.9%) | 343 (1.7%) | 0.07 | 15,699 (2.3%) | 914 (3.2%) | 0.06 |
| Hypertension | 16,428 (0.7%) | 1,187 (5.1%) | 0.26 | 44,886 (4.7%) | 4,809 (23.6%) | 0.56 | 88,932 (13.0%) | 10,901 (38.1%) | 0.60 |
| Retinopathy | 1,105 (0.0%) | 58 (0.2%) | 0.05 | 1,254 (0.1%) | 107 (0.5%) | 0.07 | 1,694 (0.2%) | 217 (0.8%) | 0.07 |
| Hyperfiltration | 2,200 (0.1%) | 153 (0.7%) | 0.09 | 711 (0.1%) | 124 (0.6%) | 0.09 | 530 (0.1%) | 179 (0.6%) | 0.09 |
| Renin-angiotensin-system agents | 7,196 (0.3%) | 926 (3.9%) | 0.26 | 26,383 (2.8%) | 4,296 (21.1%) | 0.59 | 56,460 (8.3%) | 9,469 (33.1%) | 0.64 |
| Beta blockers | 20,645 (0.9%) | 759 (3.2%) | 0.17 | 23,324 (2.5%) | 1,830 (9.0%) | 0.28 | 37,325 (5.5%) | 4,289 (15.0%) | 0.32 |
| Calcium channel blockers | 3,937 (0.2%) | 411 (1.8%) | 0.16 | 12,811 (1.4%) | 1,982 (9.7%) | 0.37 | 30,769 (4.5%) | 5,099 (17.8%) | 0.43 |
| Diuretics | 4,262 (0.2%) | 490 (2.1%) | 0.18 | 14,545 (1.5%) | 1,838 (9.0%) | 0.34 | 35,205 (5.2%) | 4,587 (16.0%) | 0.36 |
| Thiazide diuretics | 12,223 (0.5%) | 610 (2.6%) | 0.17 | 33,604 (3.6%) | 2,390 (11.7%) | 0.31 | 63,303 (9.3%) | 5,376 (18.8%) | 0.28 |
| Other diuretics | 14,960 (0.6%) | 891 (3.8%) | 0.22 | 23,930 (2.5%) | 1,610 (7.9%) | 0.24 | 35,763 (5.2%) | 3,508 (12.3%) | 0.25 |
| Statin | 3,329 (0.1%) | 947 (4.0%) | 0.28 | 16,551 (1.8%) | 4,605 (22.6%) | 0.67 | 48,514 (7.1%) | 10,352 (36.2%) | 0.76 |
| Fibrate | 166 (0.0%) | 49 (0.2%) | 0.06 | 547 (0.1%) | 182 (0.9%) | 0.12 | 1,054 (0.2%) | 272 (1.0%) | 0.11 |
| Other lipid lowering agents | 444 (0.0%) | 48 (0.2%) | 0.06 | 1,249 (0.1%) | 241 (1.2%) | 0.13 | 3,095 (0.5%) | 572 (2.0%) | 0.14 |

All parameters are expressed in either number (percentage) or mean (SD).

DM = Diabetes mellitus; SMD = Standard mean difference.

**Supplementary table 2b. Baseline characteristics of subjects in different age groups before weighting**

|  | Age 60-69 (N=535,743) | | | Age 70-79 (N=271,410) | | | Age ≥ 80 (N=165,191) | | |
| --- | --- | --- | --- | --- | --- | --- | --- | --- | --- |
|  | No T2DM (N=506,387) | New onset T2DM (N=29,356) | SMD | No T2DM (N=254,843) | New onset T2DM (N=16,567) | SMD | No T2DM (N=158,927) | New onset T2DM (N=6,264) | SMD |
| ***United Kingdom*** |  |  |  |  |  |  |  |  |  |
| Male | 251,720 (49.7%) | 18,049 (61.5%) | 0.24 | 115,696 (45.4%) | 9,070 (54.7%) | 0.19 | 52,396 (33.0%) | 2,691 (43.0%) | 0.21 |
| Age, years | 63.9 (2.8) | 64.2 (2.8) | 0.13 | 74.0 (2.9) | 73.8 (2.8) | 0.06 | 85.7 (4.7) | 84.5 (4.0) | 0.25 |
| Current smoker | 79,868 (15.8%) | 4,576 (15.6%) | 0.01 | 26,881 (10.5%) | 1,565 (9.4%) | 0.04 | 8,384 (5.3%) | 291 (4.6%) | 0.03 |
| Obesity | 88,169 (17.4%) | 15,343 (52.3%) | 0.79 | 38,965 (15.3%) | 6,650 (40.1%) | 0.58 | 11,937 (7.5%) | 1,243 (19.8%) | 0.36 |
| Atrial fibrillation | 8,030 (1.6%) | 1,148 (3.9%) | 0.14 | 10,707 (4.2%) | 1,292 (7.8%) | 0.15 | 13,270 (8.3%) | 787 (12.6%) | 0.14 |
| Peripheral vascular disease | 4,562 (0.9%) | 603 (2.1%) | 0.10 | 4,582 (1.8%) | 502 (3.0%) | 0.08 | 3,374 (2.1%) | 203 (3.2%) | 0.07 |
| Amputation | 1,438 (0.3%) | 139 (0.5%) | 0.03 | 1,031 (0.4%) | 95 (0.6%) | 0.02 | 848 (0.5%) | 42 (0.7%) | 0.02 |
| Dementia | 1,523 (0.3%) | 102 (0.3%) | 0.01 | 5,553 (2.2%) | 279 (1.7%) | 0.04 | 15,564 (9.8%) | 510 (8.1%) | 0.06 |
| Chronic lung disease | 18,505 (3.7%) | 2,031 (6.9%) | 0.15 | 16,524 (6.5%) | 1,569 (9.5%) | 0.11 | 9,302 (5.9%) | 492 (7.9%) | 0.08 |
| Connective tissue disease | 10,843 (2.1%) | 798 (2.7%) | 0.04 | 8,853 (3.5%) | 649 (3.9%) | 0.02 | 6,935 (4.4%) | 300 (4.8%) | 0.02 |
| Peptic ulcer disease | 10,966 (2.2%) | 848 (2.9%) | 0.05 | 8,083 (3.2%) | 628 (3.8%) | 0.03 | 5,304 (3.3%) | 209 (3.3%) | 0.00 |
| Liver disease | 1,354 (0.3%) | 200 (0.7%) | 0.06 | 642 (0.3%) | 75 (0.5%) | 0.03 | 274 (0.2%) | 15 (0.2%) | 0.01 |
| Cardiovascular disease | 31,775 (6.3%) | 4,363 (14.9%) | 0.28 | 32,473 (12.7%) | 3,571 (21.6%) | 0.24 | 28,573 (18.0%) | 1,501 (24.0%) | 0.15 |
| Hemiplegia | 722 (0.1%) | 55 (0.2%) | 0.01 | 513 (0.2%) | 47 (0.3%) | 0.02 | 354 (0.2%) | 12 (0.2%) | 0.01 |
| Leukemia | 673 (0.1%) | 52 (0.2%) | 0.01 | 588 (0.2%) | 47 (0.3%) | 0.01 | 452 (0.3%) | 20 (0.3%) | 0.01 |
| Malignant lymphoma | 1,380 (0.3%) | 100 (0.3%) | 0.01 | 970 (0.4%) | 67 (0.4%) | 0.00 | 573 (0.4%) | 26 (0.4%) | 0.01 |
| Cancer | 23,610 (4.7%) | 1,897 (6.5%) | 0.08 | 18,857 (7.4%) | 1,632 (9.9%) | 0.09 | 13,071 (8.2%) | 630 (10.1%) | 0.06 |
| Hypertension | 117,889 (23.3%) | 14,071 (47.9%) | 0.53 | 85,816 (33.7%) | 8,583 (51.8%) | 0.37 | 55,937 (35.2%) | 2,882 (46.0%) | 0.22 |
| Retinopathy | 1,995 (0.4%) | 272 (0.9%) | 0.07 | 1,611 (0.6%) | 223 (1.3%) | 0.07 | 1,190 (0.7%) | 100 (1.6%) | 0.08 |
| Hyperfiltration | 408 (0.1%) | 159 (0.5%) | 0.08 | 198 (0.1%) | 68 (0.4%) | 0.07 | 125 (0.1%) | 23 (0.4%) | 0.06 |
| Renin-angiotensin-system agents | 71,692 (14.2%) | 11,371 (38.7%) | 0.58 | 50,122 (19.7%) | 6,496 (39.2%) | 0.44 | 26,058 (16.4%) | 2,040 (32.6%) | 0.38 |
| Beta blockers | 48,583 (9.6%) | 6,290 (21.4%) | 0.33 | 35,156 (13.8%) | 4,199 (25.3%) | 0.29 | 19,117 (12.0%) | 1,452 (23.2%) | 0.30 |
| Calcium channel blockers | 50,521 (10.0%) | 7,733 (26.3%) | 0.43 | 41,685 (16.4%) | 4,999 (30.2%) | 0.33 | 24,830 (15.6%) | 1,673 (26.7%) | 0.27 |
| Diuretics | 56,526 (11.2%) | 7,286 (24.8%) | 0.36 | 49,601 (19.5%) | 5,501 (33.2%) | 0.32 | 37,022 (23.3%) | 2,295 (36.6%) | 0.29 |
| Thiazide diuretics | 88,706 (17.5%) | 7,650 (26.1%) | 0.21 | 65,583 (25.7%) | 4,877 (29.4%) | 0.08 | 35,226 (22.2%) | 1,562 (24.9%) | 0.07 |
| Other diuretics | 55,698 (11.0%) | 5,707 (19.4%) | 0.24 | 59,937 (23.5%) | 4,923 (29.7%) | 0.14 | 56,421 (35.5%) | 2,519 (40.2%) | 0.10 |
| Statin | 84,915 (16.8%) | 14,343 (48.9%) | 0.73 | 64,269 (25.2%) | 8,680 (52.4%) | 0.58 | 25,323 (15.9%) | 2,492 (39.8%) | 0.55 |
| Fibrate | 1,360 (0.3%) | 283 (1.0%) | 0.09 | 972 (0.4%) | 126 (0.8%) | 0.05 | 290 (0.2%) | 23 (0.4%) | 0.04 |
| Other lipid lowering agents | 5,094 (1.0%) | 792 (2.7%) | 0.13 | 3,173 (1.2%) | 427 (2.6%) | 0.10 | 808 (0.5%) | 79 (1.3%) | 0.08 |

All parameters are expressed in either number (percentage) or mean (SD).

DM = Diabetes mellitus; SMD = Standard mean difference.

**Supplementary table 2c. Baseline characteristics of subjects in different age groups before weighting**

|  | Age 18-39 (N=1,285,362) | | | Age 40-49 (N=707,852) | | | Age 50-59 (N=711,423) | | |
| --- | --- | --- | --- | --- | --- | --- | --- | --- | --- |
|  | No T2DM (N=1,276,571) | New onset T2DM (N=8,791) | SMD | No T2DM (N=680,899) | New onset T2DM (N=26,953) | SMD | No T2DM (N=653,332) | New onset T2DM (N=58,091) | SMD |
| ***Hong Kong*** |  | | |  | | |  | | |
| Male | 563,829 (44.2%) | 4,598 (52.3%) | 0.16 | 285,041 (41.9%) | 15,358 (57.0%) | 0.31 | 300,642 (46.0%) | 31,552 (54.3%) | 0.17 |
| Age, years | 29.0 (6.2) | 33.9 (4.8) | 0.80 | 44.7 (2.9) | 45.5 (2.8) | 0.29 | 54.2 (2.9) | 54.7 (2.8) | 0.17 |
| Current smoker | 4,324 (0.3%) | 177 (2.0%) | 0.16 | 3,575 (0.5%) | 710 (2.6%) | 0.17 | 4,797 (0.7%) | 1,494 (2.6%) | 0.14 |
| Obesity | 29,947 (2.3%) | 3,328 (37.9%) | 0.99 | 44,481 (6.5%) | 10,148 (37.7%) | 0.81 | 57,178 (8.8%) | 19,987 (34.4%) | 0.66 |
| Atrial fibrillation | 437 (0.0%) | 11 (0.1%) | 0.03 | 1,057 (0.2%) | 105 (0.4%) | 0.04 | 2,707 (0.4%) | 516 (0.9%) | 0.06 |
| Peripheral vascular disease | 91 (0.0%) | 14 (0.2%) | 0.05 | 149 (0.0%) | 69 (0.3%) | 0.06 | 293 (0.0%) | 173 (0.3%) | 0.06 |
| Amputation | 501 (0.0%) | 11 (0.1%) | 0.03 | 438 (0.1%) | 33 (0.1%) | 0.02 | 467 (0.1%) | 65 (0.1%) | 0.01 |
| Dementia | 45 (0.0%) | 0 (0.0%) | 0.01 | 50 (0.0%) | 3 (0.0%) | 0.00 | 174 (0.0%) | 35 (0.1%) | 0.02 |
| Chronic lung disease | 561 (0.0%) | 15 (0.2%) | 0.04 | 731 (0.1%) | 56 (0.2%) | 0.03 | 2,627 (0.4%) | 312 (0.5%) | 0.02 |
| Connective tissue disease | 1,629 (0.1%) | 25 (0.3%) | 0.03 | 1,302 (0.2%) | 55 (0.2%) | 0.00 | 1,320 (0.2%) | 167 (0.3%) | 0.02 |
| Peptic ulcer disease | 4,035 (0.3%) | 35 (0.4%) | 0.01 | 4,138 (0.6%) | 211 (0.8%) | 0.02 | 6,881 (1.1%) | 695 (1.2%) | 0.01 |
| Liver disease | 3,690 (0.3%) | 382 (4.3%) | 0.27 | 3,874 (0.6%) | 939 (3.5%) | 0.21 | 5,126 (0.8%) | 1,853 (3.2%) | 0.17 |
| Cardiovascular disease | 2,168 (0.2%) | 163 (1.9%) | 0.17 | 6,215 (0.9%) | 1,408 (5.2%) | 0.25 | 16,226 (2.5%) | 4,720 (8.1%) | 0.25 |
| Hemiplegia | 979 (0.1%) | 35 (0.4%) | 0.07 | 856 (0.1%) | 158 (0.6%) | 0.08 | 1,564 (0.2%) | 524 (0.9%) | 0.09 |
| Leukemia | 475 (0.0%) | 17 (0.2%) | 0.05 | 304 (0.0%) | 29 (0.1%) | 0.02 | 302 (0.0%) | 40 (0.1%) | 0.01 |
| Malignant lymphoma | 287 (0.0%) | 12 (0.1%) | 0.04 | 228 (0.0%) | 12 (0.0%) | 0.01 | 385 (0.1%) | 47 (0.1%) | 0.01 |
| Cancer | 7,142 (0.6%) | 154 (1.8%) | 0.11 | 12,670 (1.9%) | 663 (2.5%) | 0.04 | 18,131 (2.8%) | 1,998 (3.4%) | 0.04 |
| Hypertension | 5,996 (0.5%) | 1,418 (16.1%) | 0.59 | 32,800 (4.8%) | 8,637 (32.0%) | 0.75 | 83,709 (12.8%) | 25,186 (43.4%) | 0.72 |
| Retinopathy | 42 (0.0%) | 51 (0.6%) | 0.11 | 68 (0.0%) | 179 (0.7%) | 0.11 | 145 (0.0%) | 368 (0.6%) | 0.11 |
| Hyperfiltration | 7,435 (0.6%) | 186 (2.1%) | 0.13 | 127 (0.0%) | 16 (0.1%) | 0.02 | 27 (0.0%) | 17 (0.0%) | 0.02 |
| Renin-angiotensin-system agents | 3,572 (0.3%) | 1,093 (12.4%) | 0.51 | 12,064 (1.8%) | 5,374 (19.9%) | 0.61 | 24,698 (3.8%) | 13,011 (22.4%) | 0.57 |
| Beta blockers | 15,618 (1.2%) | 872 (9.9%) | 0.39 | 29,274 (4.3%) | 4,716 (17.5%) | 0.43 | 57,281 (8.8%) | 13,299 (22.9%) | 0.39 |
| Calcium channel blockers | 5,769 (0.5%) | 1,190 (13.5%) | 0.53 | 21,942 (3.2%) | 6,262 (23.2%) | 0.62 | 51,397 (7.9%) | 17,597 (30.3%) | 0.60 |
| Diuretics | 2,392 (0.2%) | 289 (3.3%) | 0.24 | 8,694 (1.3%) | 1,794 (6.7%) | 0.28 | 23,170 (3.5%) | 5,355 (9.2%) | 0.23 |
| Thiazide diuretics | 684 (0.1%) | 139 (1.6%) | 0.17 | 3,813 (0.6%) | 869 (3.2%) | 0.20 | 10,746 (1.6%) | 2,551 (4.4%) | 0.16 |
| Other diuretics | 1,745 (0.1%) | 160 (1.8%) | 0.17 | 5,025 (0.7%) | 963 (3.6%) | 0.20 | 12,797 (2.0%) | 2,897 (5.0%) | 0.17 |
| Statin | 1,122 (0.1%) | 654 (7.4%) | 0.39 | 4,318 (0.6%) | 3,464 (12.9%) | 0.50 | 10,997 (1.7%) | 10,979 (18.9%) | 0.59 |
| Fibrate | 171 (0.0%) | 222 (2.5%) | 0.23 | 817 (0.1%) | 843 (3.1%) | 0.24 | 1,833 (0.3%) | 1,610 (2.8%) | 0.20 |
| Other lipid lowering agents | 68 (0.0%) | 7 (0.1%) | 0.04 | 116 (0.0%) | 35 (0.1%) | 0.04 | 230 (0.0%) | 80 (0.1%) | 0.03 |

All parameters are expressed in either number (percentage) or mean (SD).

DM = Diabetes mellitus; SMD = Standard mean difference.

**Supplementary table 2d. Baseline characteristics of subjects in different age groups before weighting**

|  | Age 60-69 (N=396,836) | | | Age 70-79 (N=257,295) | | | Age ≥ 80 (N=115,208) | | |
| --- | --- | --- | --- | --- | --- | --- | --- | --- | --- |
|  | No T2DM (N=345,681) | New onset T2DM (N=51,155) | SMD | No T2DM (N=228,639) | New onset T2DM (N=28,656) | SMD | No T2DM (N=105,740) | New onset T2DM (N=9,468) | SMD |
| ***Hong Kong*** |  |  |  |  |  |  |  |  |  |
| Male | 177,403 (51.3%) | 27,249 (53.3%) | 0.04 | 114,364 (50.0%) | 13,958 (48.7%) | 0.03 | 40,977 (38.8%) | 3,808 (40.2%) | 0.03 |
| Age, years | 63.8 (2.9) | 64.0 (2.8) | 0.06 | 74.1 (2.8) | 74.0 (2.8) | 0.04 | 84.6 (4.2) | 83.5 (3.4) | 0.25 |
| Current smoker | 3,831 (1.1%) | 1,342 (2.6%) | 0.11 | 2,999 (1.3%) | 578 (2.0%) | 0.06 | 722 (0.7%) | 105 (1.1%) | 0.05 |
| Obesity | 33,146 (9.6%) | 16,232 (31.7%) | 0.57 | 18,326 (8.0%) | 7,499 (26.2%) | 0.50 | 4,705 (4.4%) | 1,697 (17.9%) | 0.44 |
| Atrial fibrillation | 3,312 (1.0%) | 899 (1.8%) | 0.07 | 4,569 (2.0%) | 958 (3.3%) | 0.08 | 3,813 (3.6%) | 533 (5.6%) | 0.10 |
| Peripheral vascular disease | 271 (0.1%) | 211 (0.4%) | 0.07 | 368 (0.2%) | 162 (0.6%) | 0.07 | 282 (0.3%) | 62 (0.7%) | 0.06 |
| Amputation | 162 (0.0%) | 52 (0.1%) | 0.02 | 112 (0.0%) | 22 (0.1%) | 0.01 | 88 (0.1%) | 7 (0.1%) | 0.00 |
| Dementia | 448 (0.1%) | 109 (0.2%) | 0.02 | 1,981 (0.9%) | 297 (1.0%) | 0.02 | 4,063 (3.8%) | 349 (3.7%) | 0.01 |
| Chronic lung disease | 5,090 (1.5%) | 810 (1.6%) | 0.01 | 9,641 (4.2%) | 1,066 (3.7%) | 0.03 | 6,437 (6.1%) | 557 (5.9%) | 0.01 |
| Connective tissue disease | 571 (0.2%) | 112 (0.2%) | 0.01 | 419 (0.2%) | 71 (0.2%) | 0.01 | 151 (0.1%) | 16 (0.2%) | 0.01 |
| Peptic ulcer disease | 5,143 (1.5%) | 877 (1.7%) | 0.02 | 5,274 (2.3%) | 734 (2.6%) | 0.02 | 3,557 (3.4%) | 340 (3.6%) | 0.01 |
| Liver disease | 3,017 (0.9%) | 1,421 (2.8%) | 0.14 | 2,154 (0.9%) | 643 (2.2%) | 0.10 | 1,021 (1.0%) | 192 (2.0%) | 0.09 |
| Cardiovascular disease | 19,468 (5.6%) | 6,625 (13.0%) | 0.25 | 24,927 (10.9%) | 5,811 (20.3%) | 0.26 | 17,072 (16.1%) | 2,506 (26.5%) | 0.25 |
| Hemiplegia | 1,737 (0.5%) | 739 (1.4%) | 0.10 | 2,348 (1.0%) | 665 (2.3%) | 0.10 | 1,843 (1.7%) | 308 (3.3%) | 0.10 |
| Leukemia | 130 (0.0%) | 39 (0.1%) | 0.02 | 69 (0.0%) | 5 (0.0%) | 0.01 | 30 (0.0%) | 5 (0.1%) | 0.01 |
| Malignant lymphoma | 265 (0.1%) | 55 (0.1%) | 0.01 | 238 (0.1%) | 31 (0.1%) | 0.00 | 89 (0.1%) | 6 (0.1%) | 0.01 |
| Cancer | 12,050 (3.5%) | 2,414 (4.7%) | 0.06 | 11,103 (4.9%) | 1,790 (6.2%) | 0.06 | 5,400 (5.1%) | 680 (7.2%) | 0.09 |
| Hypertension | 80,099 (23.2%) | 27,832 (54.4%) | 0.68 | 76,440 (33.4%) | 17,894 (62.4%) | 0.61 | 39,677 (37.5%) | 5,999 (63.4%) | 0.53 |
| Retinopathy | 142 (0.0%) | 342 (0.7%) | 0.11 | 171 (0.1%) | 188 (0.7%) | 0.10 | 84 (0.1%) | 61 (0.6%) | 0.09 |
| Hyperfiltration | 9 (0.0%) | 4 (0.0%) | 0.01 | 2 (0.0%) | 4 (0.0%) | 0.02 | 2 (0.0%) | 1 (0.0%) | 0.01 |
| Renin-angiotensin-system agents | 21,356 (6.2%) | 12,737 (24.9%) | 0.53 | 20,971 (9.2%) | 7,581 (26.5%) | 0.46 | 10,783 (10.2%) | 2,518 (26.6%) | 0.43 |
| Beta blockers | 48,017 (13.9%) | 14,117 (27.6%) | 0.34 | 42,103 (18.4%) | 8,730 (30.5%) | 0.28 | 17,653 (16.7%) | 2,614 (27.6%) | 0.27 |
| Calcium channel blockers | 51,274 (14.8%) | 19,986 (39.1%) | 0.57 | 52,862 (23.1%) | 13,447 (46.9%) | 0.52 | 28,774 (27.2%) | 4,845 (51.2%) | 0.51 |
| Diuretics | 22,876 (6.6%) | 6,336 (12.4%) | 0.20 | 25,369 (11.1%) | 4,822 (16.8%) | 0.17 | 14,250 (13.5%) | 1,744 (18.4%) | 0.14 |
| Thiazide diuretics | 10,608 (3.1%) | 3,026 (5.9%) | 0.14 | 10,506 (4.6%) | 2,040 (7.1%) | 0.11 | 5,163 (4.9%) | 596 (6.3%) | 0.06 |
| Other diuretics | 12,642 (3.7%) | 3,447 (6.7%) | 0.14 | 15,379 (6.7%) | 2,889 (10.1%) | 0.12 | 9,381 (8.9%) | 1,198 (12.7%) | 0.12 |
| Statin | 12,190 (3.5%) | 12,457 (24.4%) | 0.63 | 11,373 (5.0%) | 7,289 (25.4%) | 0.59 | 4,055 (3.8%) | 2,178 (23.0%) | 0.59 |
| Fibrate | 1,963 (0.6%) | 1,286 (2.5%) | 0.16 | 2,101 (0.9%) | 592 (2.1%) | 0.09 | 741 (0.7%) | 153 (1.6%) | 0.09 |
| Other lipid lowering agents | 183 (0.1%) | 76 (0.1%) | 0.03 | 155 (0.1%) | 39 (0.1%) | 0.02 | 44 (0.0%) | 13 (0.1%) | 0.03 |

All parameters are expressed in either number (percentage) or mean (SD).

DM = Diabetes mellitus; SMD = Standard mean difference.

**Supplementary table 3. Baseline clinical characteristics of in different age groups among patients with new onset Type 2 Diabetes mellitus**

|  | Age 18-39 | | Age 40-49 | | Age 50-59 | |
| --- | --- | --- | --- | --- | --- | --- |
|  | United Kingdom (N=23,479) | Hong Kong (N=8,791) | United Kingdom (N=20,353) | Hong Kong (N=26,953) | United Kingdom (N=28,591) | Hong Kong (N=58,091) |
| Body Mass Index, kg/m^2^ | 49.8 (2408.5) | 29.2 (6.1) | 60.6 (2505.2) | 27.8 (4.8) | 101.8 (4160.6) | 26.8 (4.3) |
| Systolic Blood Pressure, mmHg | 122.5 (15.3) | 131.5 (18.2) | 134.2 (17.1) | 134.5 (18.6) | 138.0 (17.2) | 136.9 (18.8) |
| Diastolic Blood Pressure, mmHg | 76.5 (11.1) | 81.3 (12.2) | 83.8 (11.1) | 82.3 (11.4) | 83.7 (10.3) | 81.2 (10.7) |
| Fasting glucose, mmol/L | 7.5 (4.0) | 9.2 (3.6) | 9.6 (15.0) | 9.1 (3.2) | 9.0 (3.7) | 8.7 (3.0) |
| Haemoglobin A1c, % | 7.9 (3.4) | 8.8 (2.7) | 8.4 (2.8) | 8.7 (2.4) | 8.2 (2.8) | 8.4 (2.3) |
| Low-density lipoprotein-cholesterol, mmol/L | 3.2 (6.2) | 3.2 (1.0) | 3.3 (1.0) | 3.2 (1.0) | 3.3 (3.1) | 3.3 (1.0) |
| Estimated glomerular filtration rate, mL/min/1.73m^2^ | 88.1 (159.1) | 113.4 (14.1) | 88.3 (238.5) | 102.9 (12.8) | 89.9 (457.1) | 96.5 (12.0) |
|  | Age 60-69 | | Age 70-79 | | Age ≥ 80 | |
|  | United Kingdom (N=29,356) | Hong Kong (N=51,155) | United Kingdom (N=16,567) | Hong Kong (N=28,656) | United Kingdom (N=6,264) | Hong Kong (N=9,468) |
| Body Mass Index, kg/m^2^ | 69.8 (3002.4) | 26.2 (4.0) | 46.7 (1292.1) | 25.7 (3.8) | 114.6 (3754.3) | 25.1 (3.8) |
| Systolic Blood Pressure, mmHg | 139.8 (17.1) | 139.4 (18.8) | 139.8 (17.2) | 140.8 (19.2) | 139.0 (18.2) | 140.8 (19.8) |
| Diastolic Blood Pressure, mmHg | 81.0 (9.9) | 78.6 (10.5) | 77.8 (10.0) | 74.4 (10.5) | 75.4 (10.2) | 71.3 (10.7) |
| Fasting glucose, mmol/L | 8.4 (3.2) | 8.3 (2.8) | 8.0 (3.0) | 8.0 (2.5) | 7.7 (2.5) | 7.9 (2.5) |
| Haemoglobin A1c, % | 7.8 (2.5) | 8.0 (2.1) | 7.5 (2.4) | 7.8 (2.0) | 7.4 (2.1) | 7.6 (1.9) |
| Low-density lipoprotein-cholesterol, mmol/L | 3.1 (4.8) | 3.2 (1.0) | 2.8 (1.0) | 3.1 (0.9) | 2.7 (1.0) | 3.0 (0.9) |
| Estimated glomerular filtration rate, mL/min/1.73m^2^ | 87.7 (249.3) | 89.0 (11.7) | 88.0 (302.7) | 82.2 (10.9) | 84.9 (202.7) | 78.6 (9.7) |

All parameters are expressed in mean (SD).

Supplementary Table 4a. Comparison of baseline characteristics between the UK and HK cohorts before weighting

|  | Age 18-39 (N=3,729,169) | | | Age 40-49 (N=1,673,238) | | | Age 50-59 (N=1,423,315) | | |
| --- | --- | --- | --- | --- | --- | --- | --- | --- | --- |
|  | UK cohort (N=2,443,807) | HK cohort (N=1,285,362) | SMD | UK cohort (N=965,386) | HK cohort (N=707,852) | SMD | UK cohort (N=711,892) | HK cohort (N=711,423) | SMD |
| Male | 1,157,817 (47.4%) | 568,427 (44.2%) | 0.06 | 498,093 (51.6%) | 300,399 (42.4%) | 0.18 | 362,033 (50.9%) | 332,194 (46.7%) | 0.08 |
| Age, years | 28.4 (6.2) | 29.0 (6.2) | 0.10 | 44.3 (2.8) | 44.8 (2.9) | 0.15 | 54.3 (2.9) | 54.2 (2.9) | 0.02 |
| Current smoker | 593,245 (24.3%) | 4,501 (0.4%) | 0.78 | 220,475 (22.8%) | 4,285 (0.6%) | 0.74 | 144,408 (20.3%) | 6,291 (0.9%) | 0.66 |
| Obesity | 214,541 (8.8%) | 33,275 (2.6%) | 0.27 | 161,571 (16.7%) | 54,629 (7.7%) | 0.28 | 138,151 (19.4%) | 77,165 (10.8%) | 0.24 |
| Atrial fibrillation | 999 (0.0%) | 448 (0.0%) | 0.00 | 2,014 (0.2%) | 1,162 (0.2%) | 0.01 | 4,347 (0.6%) | 3,223 (0.5%) | 0.02 |
| Peripheral vascular disease | 117 (0.0%) | 105 (0.0%) | 0.00 | 497 (0.1%) | 218 (0.0%) | 0.01 | 2,312 (0.3%) | 466 (0.1%) | 0.06 |
| Amputation | 1,867 (0.1%) | 512 (0.0%) | 0.02 | 1,574 (0.2%) | 471 (0.1%) | 0.03 | 1,525 (0.2%) | 532 (0.1%) | 0.04 |
| Dementia | 20 (0.0%) | 45 (0.0%) | 0.01 | 78 (0.0%) | 53 (0.0%) | 0.00 | 384 (0.1%) | 209 (0.0%) | 0.01 |
| Lung | 667 (0.0%) | 576 (0.0%) | 0.01 | 3,251 (0.3%) | 787 (0.1%) | 0.05 | 10,210 (1.4%) | 2,939 (0.4%) | 0.11 |
| Cumulative trauma disorder | 7,068 (0.3%) | 1,654 (0.1%) | 0.04 | 7,720 (0.8%) | 1,357 (0.2%) | 0.09 | 10,035 (1.4%) | 1,487 (0.2%) | 0.13 |
| Peptic ulcer | 4,390 (0.2%) | 4,070 (0.3%) | 0.03 | 7,853 (0.8%) | 4,349 (0.6%) | 0.02 | 10,802 (1.5%) | 7,576 (1.1%) | 0.04 |
| Liver | 1,381 (0.1%) | 4,072 (0.3%) | 0.06 | 1,731 (0.2%) | 4,813 (0.7%) | 0.08 | 1,974 (0.3%) | 6,979 (1.0%) | 0.09 |
| CVD | 2,823 (0.1%) | 2,331 (0.2%) | 0.02 | 7,367 (0.8%) | 7,623 (1.1%) | 0.03 | 20,100 (2.8%) | 20,946 (2.9%) | 0.01 |
| Hemiplegia | 1,563 (0.1%) | 1,014 (0.1%) | 0.01 | 864 (0.1%) | 1,014 (0.1%) | 0.02 | 855 (0.1%) | 2,088 (0.3%) | 0.04 |
| Leukemia | 702 (0.0%) | 492 (0.0%) | 0.01 | 286 (0.0%) | 333 (0.0%) | 0.01 | 441 (0.1%) | 342 (0.0%) | 0.01 |
| Malignant_lymphoma | 1,264 (0.1%) | 299 (0.0%) | 0.01 | 1,077 (0.1%) | 240 (0.0%) | 0.03 | 1,168 (0.2%) | 432 (0.1%) | 0.03 |
| Cancer | 6,111 (0.3%) | 7,296 (0.6%) | 0.05 | 9,259 (1.0%) | 13,333 (1.9%) | 0.08 | 16,613 (2.3%) | 20,129 (2.8%) | 0.03 |
| Hypertension | 17,615 (0.7%) | 7,414 (0.6%) | 0.02 | 49,695 (5.1%) | 41,437 (5.9%) | 0.03 | 99,833 (14.0%) | 108,895 (15.3%) | 0.04 |
| Retinopathy | 1,163 (0.0%) | 93 (0.0%) | 0.02 | 1,361 (0.1%) | 247 (0.0%) | 0.04 | 1,911 (0.3%) | 513 (0.1%) | 0.05 |
| Hyperfiltration | 2,353 (0.1%) | 7,621 (0.6%) | 0.08 | 835 (0.1%) | 143 (0.0%) | 0.03 | 709 (0.1%) | 44 (0.0%) | 0.04 |
| Renin-angiotensin-system agents | 8,122 (0.3%) | 4,665 (0.4%) | 0.01 | 30,679 (3.2%) | 17,438 (2.5%) | 0.04 | 65,929 (9.3%) | 37,709 (5.3%) | 0.15 |
| Beta blockers | 21,404 (0.9%) | 16,490 (1.3%) | 0.04 | 25,154 (2.6%) | 33,990 (4.8%) | 0.12 | 41,614 (5.8%) | 70,580 (9.9%) | 0.15 |
| Calcium channel blockers | 4,348 (0.2%) | 6,959 (0.5%) | 0.06 | 14,793 (1.5%) | 28,204 (4.0%) | 0.15 | 35,868 (5.0%) | 68,994 (9.7%) | 0.18 |
| Diuretics | 4,752 (0.2%) | 2,681 (0.2%) | 0.00 | 16,383 (1.7%) | 10,488 (1.5%) | 0.02 | 39,792 (5.6%) | 28,525 (4.0%) | 0.07 |
| Statin | 4,276 (0.2%) | 1,776 (0.1%) | 0.01 | 21,156 (2.2%) | 7,782 (1.1%) | 0.09 | 58,866 (8.3%) | 21,976 (3.1%) | 0.23 |
| Fibrate | 215 (0.0%) | 393 (0.0%) | 0.02 | 729 (0.1%) | 1,660 (0.2%) | 0.04 | 1,326 (0.2%) | 3,443 (0.5%) | 0.05 |
| Other lipid lowering agents | 492 (0.0%) | 75 (0.0%) | 0.01 | 1,490 (0.2%) | 151 (0.0%) | 0.04 | 3,667 (0.5%) | 310 (0.0%) | 0.09 |

All parameters are expressed in either number (percentage) or mean (SD).

DM = Diabetes mellitus; SMD = Standard mean difference.

Supplementary Table 4b. Comparison of baseline characteristics between the UK and HK cohorts before weighting

|  | Age 60-69 (N=932,579) | | | Age 70-79 (N=528,705) | | | Age ≥ 80 (N=280,399) | | |
| --- | --- | --- | --- | --- | --- | --- | --- | --- | --- |
|  | UK cohort (N=535,743) | HK cohort (N=396,836) | SMD | UK cohort (N=271,410) | HK cohort (N=257,295) | SMD | UK cohort (N=165,191) | HK cohort (N=115,208) | SMD |
| Male | 269,769 (50.4%) | 204,652 (51.6%) | 0.02 | 124,766 (46.0%) | 128,322 (49.9%) | 0.08 | 55,087 (33.3%) | 44,785 (38.9%) | 0.12 |
| Age, years | 63.9 (2.8) | 63.8 (2.9) | 0.03 | 74.0 (2.9) | 74.1 (2.8) | 0.05 | 85.6 (4.6) | 84.5 (4.1) | 0.26 |
| Current smoker | 84,444 (15.8%) | 5,173 (1.3%) | 0.54 | 28,446 (10.5%) | 3,577 (1.4%) | 0.39 | 8,675 (5.3%) | 827 (0.7%) | 0.27 |
| Obesity | 103,512 (19.3%) | 49,378 (12.4%) | 0.19 | 45,615 (16.8%) | 25,825 (10.0%) | 0.20 | 13,180 (8.0%) | 6,402 (5.6%) | 0.10 |
| Atrial fibrillation | 9,178 (1.7%) | 4,211 (1.1%) | 0.06 | 11,999 (4.4%) | 5,527 (2.1%) | 0.13 | 14,057 (8.5%) | 4,346 (3.8%) | 0.20 |
| Peripheral vascular disease | 5,165 (1.0%) | 482 (0.1%) | 0.11 | 5,084 (1.9%) | 530 (0.2%) | 0.16 | 3,577 (2.2%) | 344 (0.3%) | 0.17 |
| Amputation | 1,577 (0.3%) | 214 (0.1%) | 0.06 | 1,126 (0.4%) | 134 (0.1%) | 0.08 | 890 (0.5%) | 95 (0.1%) | 0.08 |
| Dementia | 1,625 (0.3%) | 557 (0.1%) | 0.03 | 5,832 (2.1%) | 2,278 (0.9%) | 0.10 | 16,074 (9.7%) | 4,412 (3.8%) | 0.24 |
| Lung | 20,536 (3.8%) | 5,900 (1.5%) | 0.15 | 18,093 (6.7%) | 10,707 (4.2%) | 0.11 | 9,794 (5.9%) | 6,994 (6.1%) | 0.01 |
| Cumulative trauma disorder | 11,641 (2.2%) | 683 (0.2%) | 0.19 | 9,502 (3.5%) | 490 (0.2%) | 0.25 | 7,235 (4.4%) | 167 (0.1%) | 0.29 |
| Peptic ulcer | 11,814 (2.2%) | 6,020 (1.5%) | 0.05 | 8,711 (3.2%) | 6,008 (2.3%) | 0.05 | 5,513 (3.3%) | 3,897 (3.4%) | 0.00 |
| Liver | 1,554 (0.3%) | 4,438 (1.1%) | 0.10 | 717 (0.3%) | 2,797 (1.1%) | 0.10 | 289 (0.2%) | 1,213 (1.1%) | 0.11 |
| CVD | 36,138 (6.7%) | 26,093 (6.6%) | 0.01 | 36,044 (13.3%) | 30,738 (11.9%) | 0.04 | 30,074 (18.2%) | 19,578 (17.0%) | 0.03 |
| Hemiplegia | 777 (0.1%) | 2,476 (0.6%) | 0.08 | 560 (0.2%) | 3,013 (1.2%) | 0.12 | 366 (0.2%) | 2,151 (1.9%) | 0.16 |
| Leukemia | 725 (0.1%) | 169 (0.0%) | 0.03 | 635 (0.2%) | 74 (0.0%) | 0.06 | 472 (0.3%) | 35 (0.0%) | 0.06 |
| Malignant_lymphoma | 1,480 (0.3%) | 320 (0.1%) | 0.05 | 1,037 (0.4%) | 269 (0.1%) | 0.06 | 599 (0.4%) | 95 (0.1%) | 0.06 |
| Cancer | 25,507 (4.8%) | 14,464 (3.6%) | 0.06 | 20,489 (7.5%) | 12,893 (5.0%) | 0.10 | 13,701 (8.3%) | 6,080 (5.3%) | 0.12 |
| Hypertension | 131,960 (24.6%) | 107,931 (27.2%) | 0.06 | 94,399 (34.8%) | 94,334 (36.7%) | 0.04 | 58,819 (35.6%) | 45,676 (39.6%) | 0.08 |
| Retinopathy | 2,267 (0.4%) | 484 (0.1%) | 0.06 | 1,834 (0.7%) | 359 (0.1%) | 0.08 | 1,290 (0.8%) | 145 (0.1%) | 0.10 |
| Hyperfiltration | 567 (0.1%) | 13 (0.0%) | 0.04 | 266 (0.1%) | 6 (0.0%) | 0.04 | 148 (0.1%) | 3 (0.0%) | 0.04 |
| Renin-angiotensin-system agents | 83,063 (15.5%) | 34,093 (8.6%) | 0.21 | 56,618 (20.9%) | 28,552 (11.1%) | 0.27 | 28,098 (17.0%) | 13,301 (11.5%) | 0.16 |
| Calcium channel blockers | 58,254 (10.9%) | 71,260 (18.0%) | 0.20 | 46,684 (17.2%) | 66,309 (25.8%) | 0.21 | 26,503 (16.0%) | 33,619 (29.2%) | 0.32 |
| Diuretics | 63,812 (11.9%) | 29,212 (7.4%) | 0.15 | 55,102 (20.3%) | 30,191 (11.7%) | 0.24 | 39,317 (23.8%) | 15,994 (13.9%) | 0.26 |
| Statin | 99,258 (18.5%) | 24,647 (6.2%) | 0.38 | 72,949 (26.9%) | 18,662 (7.3%) | 0.54 | 27,815 (16.8%) | 6,233 (5.4%) | 0.37 |
| Fibrate | 1,643 (0.3%) | 3,249 (0.8%) | 0.07 | 1,098 (0.4%) | 2,693 (1.0%) | 0.08 | 313 (0.2%) | 894 (0.8%) | 0.08 |
| Other lipid lowering agents | 5,886 (1.1%) | 259 (0.1%) | 0.14 | 3,600 (1.3%) | 194 (0.1%) | 0.15 | 887 (0.5%) | 57 (0.0%) | 0.09 |

All parameters are expressed in either number (percentage) or mean (SD).

DM = Diabetes mellitus; SMD = Standard mean difference.

**Supplementary Table 5 Variance Inflation Factor for Baseline Characteristics**

| Variable | Variance Inflation Factor | | | | | | | | | | | |
| --- | --- | --- | --- | --- | --- | --- | --- | --- | --- | --- | --- | --- |
|  | United Kingdom Cohort | | | | | | Hong Kong cohort | | | | | |
|  | Age 18-39 | Age 40-49 | Age 50-59 | Age 60-69 | Age 70-79 | Age ≥ 80 | Age 18-39 | Age 40-49 | Age 50-59 | Age 60-69 | Age 70-79 | Age ≥ 80 |
| New onset T2DM | 1.03 | 1.08 | 1.09 | 1.08 | 1.05 | 1.03 | 1.11 | 1.17 | 1.20 | 1.21 | 1.14 | 1.11 |
| Age, years | 1.03 | 1.01 | 1.02 | 1.02 | 1.02 | 1.08 | 1.03 | 1.01 | 1.01 | 1.02 | 1.01 | 1.05 |
| Sex | 1.02 | 1.02 | 1.03 | 1.04 | 1.05 | 1.05 | 1.01 | 1.02 | 1.03 | 1.04 | 1.05 | 1.06 |
| Current smoker | 1.01 | 1.01 | 1.02 | 1.03 | 1.03 | 1.03 | 1.00 | 1.01 | 1.02 | 1.02 | 1.02 | 1.01 |
| Obesity | 1.04 | 1.06 | 1.09 | 1.10 | 1.07 | 1.05 | 1.07 | 1.12 | 1.13 | 1.12 | 1.09 | 1.06 |
| Atrial fibrillation | 1.00 | 1.01 | 1.02 | 1.04 | 1.05 | 1.06 | 1.01 | 1.02 | 1.03 | 1.03 | 1.03 | 1.05 |
| Peripheral vascular disease | 1.00 | 1.01 | 1.02 | 1.03 | 1.04 | 1.03 | 1.03 | 1.05 | 1.04 | 1.04 | 1.05 | 1.10 |
| Amputation | 1.00 | 1.00 | 1.00 | 1.00 | 1.00 | 1.00 | 1.03 | 1.05 | 1.03 | 1.03 | 1.05 | 1.09 |
| Dementia | NA | 1.00 | 1.00 | 1.00 | 1.01 | 1.03 | NA | 1.00 | 1.00 | 1.00 | 1.01 | 1.03 |
| Chronic lung disease | 1.00 | 1.00 | 1.02 | 1.03 | 1.04 | 1.04 | 1.00 | 1.00 | 1.01 | 1.02 | 1.04 | 1.05 |
| Connective tissue disease | 1.00 | 1.00 | 1.00 | 1.00 | 1.01 | 1.01 | 1.01 | 1.00 | 1.00 | 1.00 | 1.00 | 1.00 |
| Peptic ulcer disease | 1.00 | 1.00 | 1.01 | 1.01 | 1.01 | 1.01 | 1.00 | 1.00 | 1.00 | 1.01 | 1.01 | 1.01 |
| Liver disease | 1.00 | 1.00 | 1.00 | 1.00 | 1.00 | 1.00 | 1.01 | 1.01 | 1.01 | 1.01 | 1.01 | 1.01 |
| Cardiovascular disease | 1.03 | 1.13 | 1.22 | 1.26 | 1.26 | 1.21 | 1.08 | 1.28 | 1.32 | 1.35 | 1.35 | 1.30 |
| Hemiplegia | 1.00 | 1.00 | 1.00 | 1.00 | 1.00 | 1.00 | 1.02 | 1.07 | 1.08 | 1.09 | 1.09 | 1.10 |
| Leukemia | 1.13 | 1.03 | 1.02 | 1.03 | 1.03 | 1.03 | 1.07 | 1.03 | 1.02 | 1.01 | 1.01 | 1.01 |
| Malignant lymphoma | 1.19 | 1.09 | 1.05 | 1.04 | 1.03 | 1.02 | 1.04 | 1.02 | 1.02 | 1.02 | 1.02 | 1.02 |
| Cancer | 1.32 | 1.12 | 1.08 | 1.07 | 1.06 | 1.06 | 1.12 | 1.05 | 1.04 | 1.04 | 1.03 | 1.03 |
| Hypertension | 1.43 | 1.85 | 2.00 | 1.87 | 1.65 | 1.38 | 1.59 | 2.15 | 2.29 | 2.17 | 1.90 | 1.73 |
| Hyperfiltration | 1.00 | 1.00 | 1.00 | 1.00 | 1.00 | 1.00 | 1.01 | 1.00 | 1.00 | 1.00 | 1.00 | 1.00 |
| Retinopathy | 1.00 | 1.00 | 1.00 | 1.00 | 1.00 | 1.00 | 1.00 | 1.01 | 1.01 | 1.00 | 1.00 | 1.00 |
| Renin-angiotensin-system agents | 1.47 | 1.83 | 1.85 | 1.64 | 1.45 | 1.32 | 1.25 | 1.29 | 1.26 | 1.22 | 1.18 | 1.16 |
| Beta blockers | 1.03 | 1.12 | 1.22 | 1.25 | 1.22 | 1.18 | 1.08 | 1.29 | 1.37 | 1.33 | 1.26 | 1.20 |
| Calcium channel blockers | 1.15 | 1.27 | 1.31 | 1.33 | 1.30 | 1.25 | 1.43 | 1.60 | 1.62 | 1.61 | 1.50 | 1.45 |
| Diuretics | 1.10 | 1.22 | 1.30 | 1.35 | 1.33 | 1.27 | 1.09 | 1.13 | 1.16 | 1.16 | 1.14 | 1.12 |
| Statin | 1.09 | 1.27 | 1.40 | 1.43 | 1.41 | 1.36 | 1.10 | 1.28 | 1.33 | 1.36 | 1.30 | 1.19 |
| Fibrate | 1.02 | 1.02 | 1.01 | 1.01 | 1.01 | 1.00 | 1.02 | 1.02 | 1.02 | 1.02 | 1.01 | 1.01 |
| Other lipid lowering agents | 1.03 | 1.06 | 1.05 | 1.04 | 1.02 | 1.01 | 1.01 | 1.01 | 1.00 | 1.00 | 1.00 | 1.00 |

NA: not applicable due to insufficient number of cases
