# Supplementary material for: Impact of onset age of type 2 diabetes mellitus on risk of renal complications compared to age‐matched non‐diabetic patients: Two cohort studies in the United Kingdom and Hong Kong
Source: Diabetes Obes Metab. 2025 Sep 1;27(11):6577–93. doi: 10.1111/dom.70061 (PMC12515758; doi:10.1111/dom.70061)
Supplement: Supplementary file 2 — Data S2 [file DOM-27-6577-s001.pdf]

S. Figure 1. The association between onset of diabetes mellitus and kidney disease/mortality in different age groups with at least 1-year follow up using Cox regression

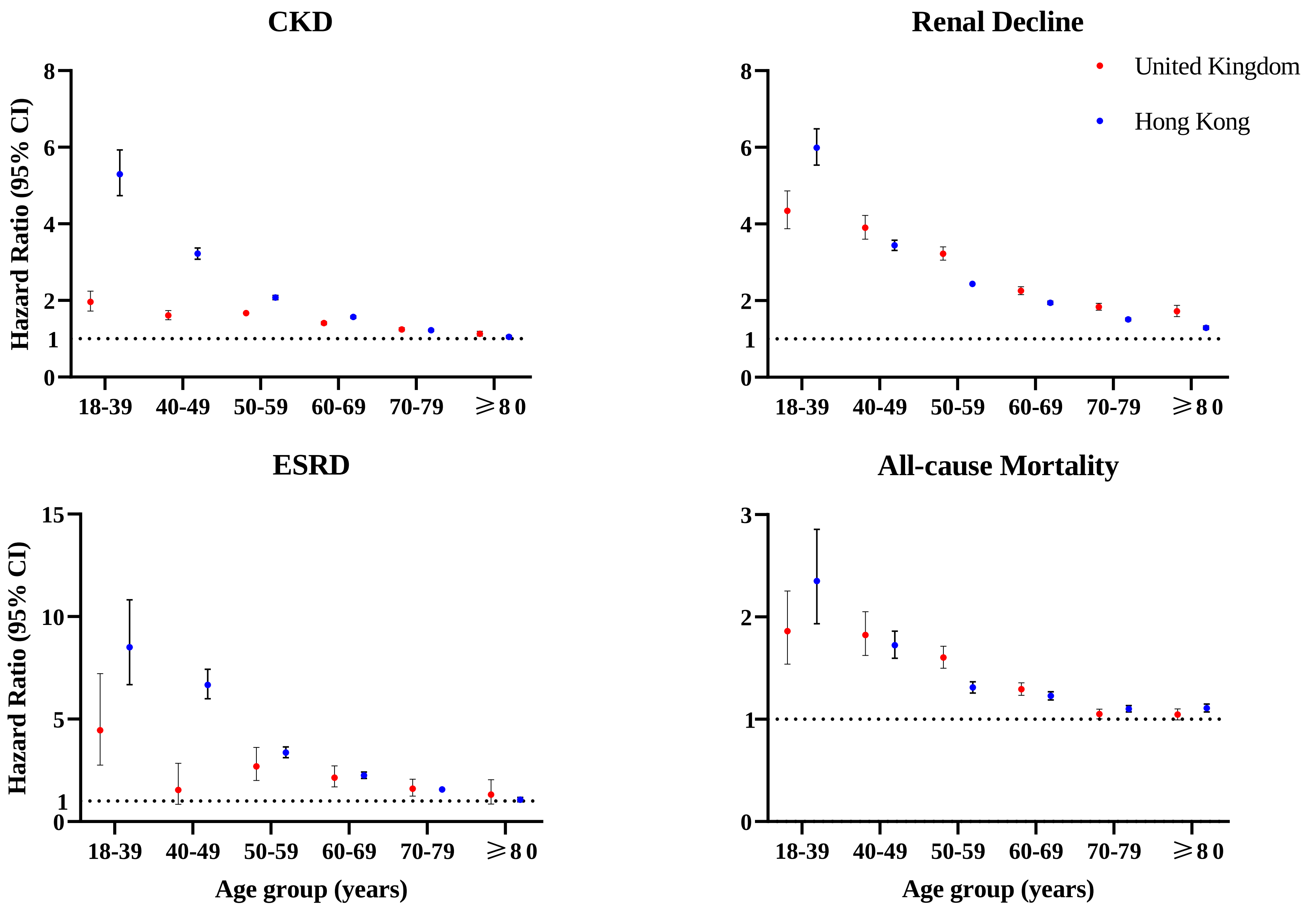

Hazard ratio with 95% confidence interval adjusted by age, sex, smoking status, comorbidities (i.e., obesity, atrial fibrillation, peripheral vascular disease, amputation, dementia, chronic lung disease, connective tissue disease, peptic ulcer disease, liver disease, cardiovascular disease, hemiplegia, leukemia, malignant lymphoma, cancer, hypertension, retinopathy, and hyperfiltration), and the use of renin-angiotensin system agents, beta-blockers, calcium channel blockers, diuretics, statins, fibrates, other lipid-lowering agents, and weighting.CKD = Chronic kidney disease; ESRD = End-stage renal disease; CI = Confidence interval.

S. Figure 2. The association between onset of diabetes mellitus and kidney disease/mortality in different age groups for age and sex matched subjects using Cox regression

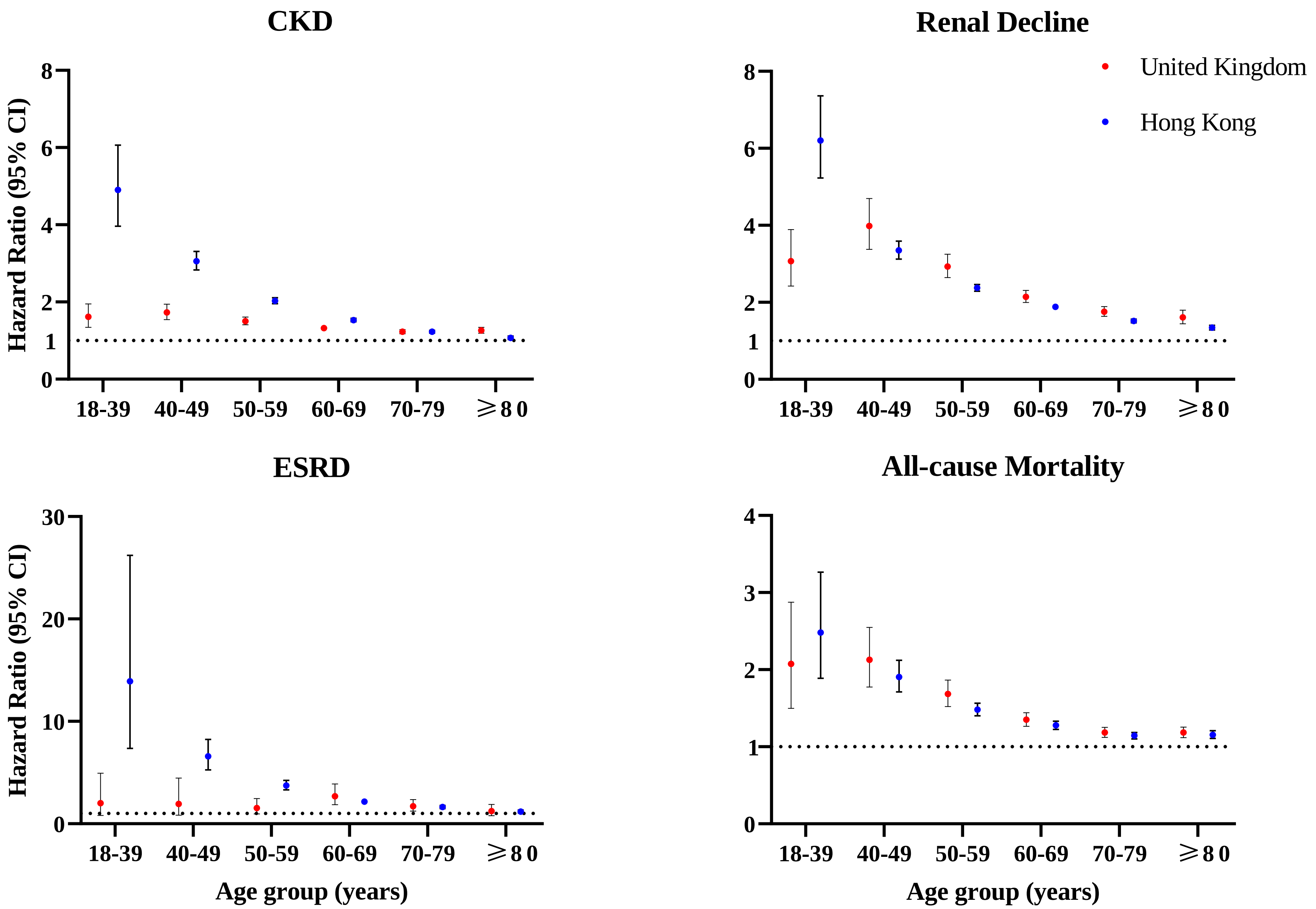

Hazard ratio with 95% confidence interval adjusted by age, sex, smoking status, comorbidities (i.e., obesity, atrial fibrillation, peripheral vascular disease, amputation, dementia, chronic lung disease, connective tissue disease, peptic ulcer disease, liver disease, cardiovascular disease, hemiplegia, leukemia, malignant lymphoma, cancer, hypertension, retinopathy, and hyperfiltration), and the use of renin-angiotensin system agents, beta-blockers, calcium channel blockers, diuretics, statins, fibrates, other lipid-lowering agents. CKD = Chronic kidney disease; ESRD = End-stage renal disease; CI = Confidence interval.

S. Figure 3. The association between onset of diabetes mellitus and kidney disease/mortality in different age groups excluding subjects with diabetes mellitus 1-year after baseline using Cox regression

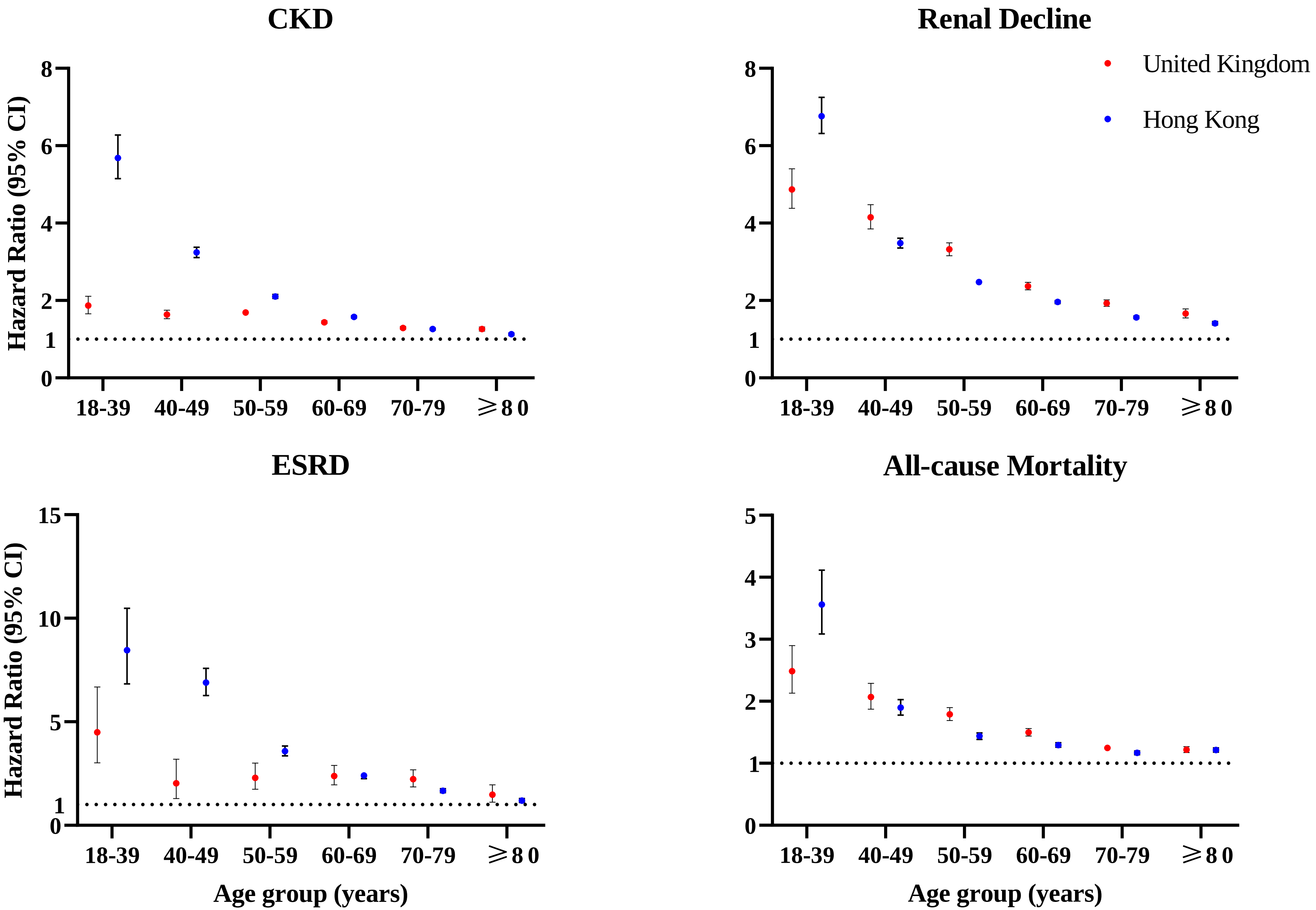

Hazard ratio with 95% confidence interval adjusted by age, sex, smoking status, comorbidities (i.e., obesity, atrial fibrillation, peripheral vascular disease, amputation, dementia, chronic lung disease, connective tissue disease, peptic ulcer disease, liver disease, cardiovascular disease, hemiplegia, leukemia, malignant lymphoma, cancer, hypertension, retinopathy, and hyperfiltration), and the use of renin-angiotensin system agents, beta-blockers, calcium channel blockers, diuretics, statins, fibrates, other lipid-lowering agents, and weighting.CKD = Chronic kidney disease; ESRD = End-stage renal disease; CI = Confidence interval.

S. Figure 4. The association between onset of diabetes mellitus and kidney disease/mortality in different age groups excluding subjects with diabetes mellitus during all the period using Cox regression

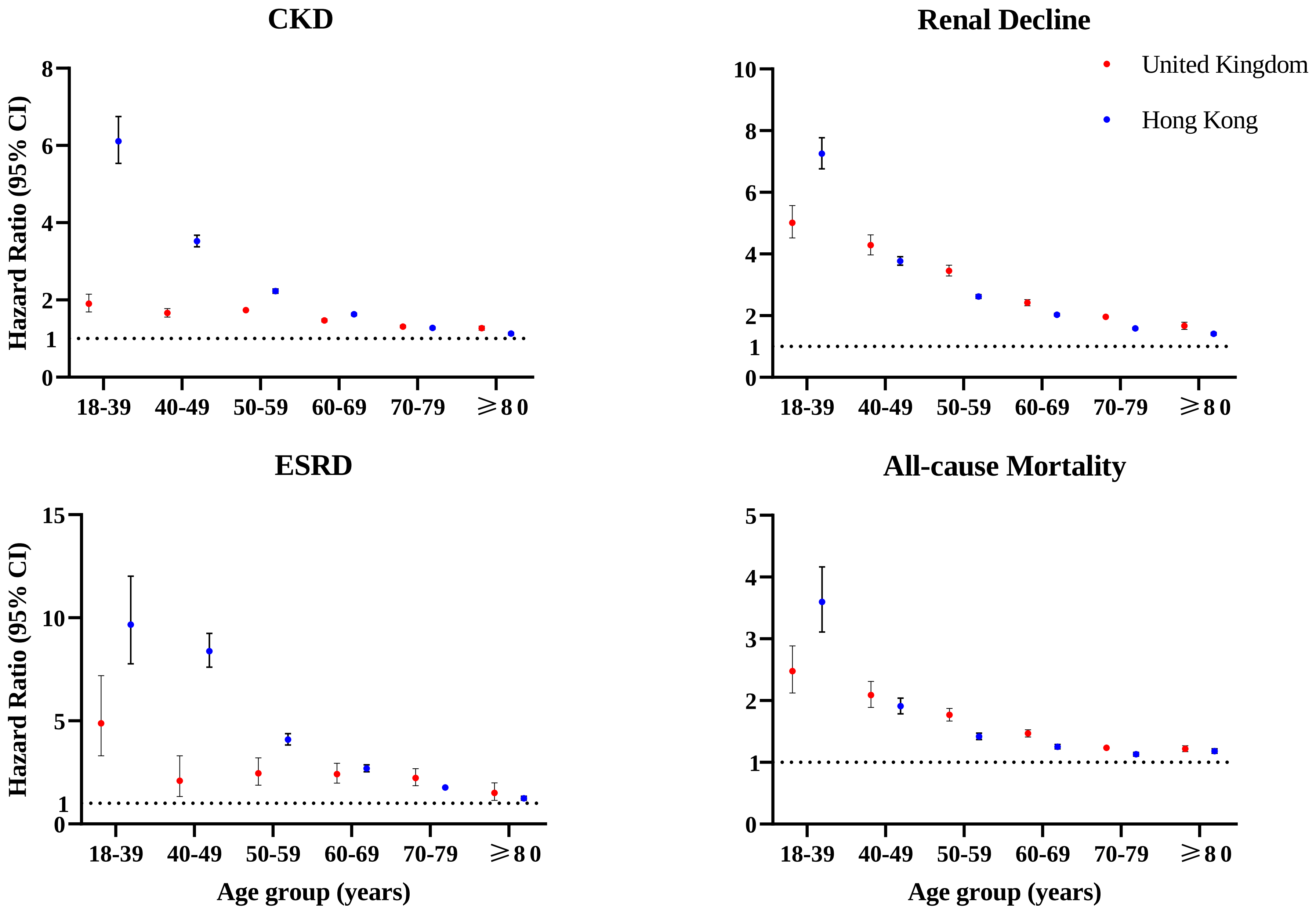

Hazard ratio with 95% confidence interval adjusted by age, sex, smoking status, comorbidities (i.e., obesity, atrial fibrillation, peripheral vascular disease, amputation, dementia, chronic lung disease, connective tissue disease, peptic ulcer disease, liver disease, cardiovascular disease, hemiplegia, leukemia, malignant lymphoma, cancer, hypertension, retinopathy, and hyperfiltration), and the use of renin-angiotensin system agents, beta-blockers, calcium channel blockers, diuretics, statins, fibrates, other lipid-lowering agents, and weighting.CKD = Chronic kidney disease; ESRD = End-stage renal disease; CI = Confidence interval.

S. Figure 5. The association between onset of diabetes mellitus and kidney disease/mortality in different age groups for 1:1 matched subject using Cox regression

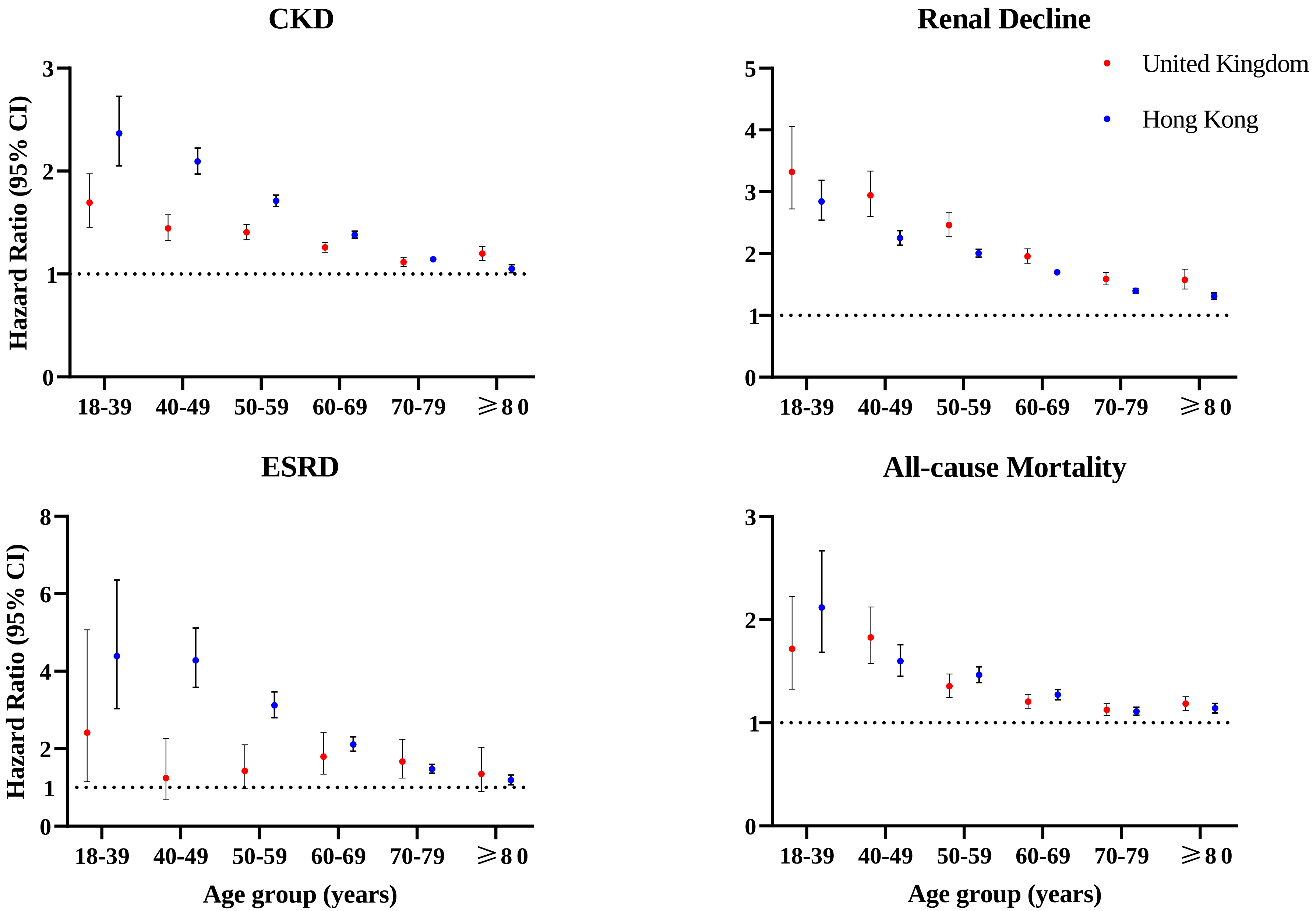

Hazard ratio with 95% confidence interval adjusted by age, sex, smoking status, comorbidities (i.e., obesity, atrial fibrillation, peripheral vascular disease, amputation, dementia, chronic lung disease, connective tissue disease, peptic ulcer disease, liver disease, cardiovascular disease, hemiplegia, leukemia, malignant lymphoma, cancer, hypertension, retinopathy, and hyperfiltration), and the use of renin-angiotensin system agents, beta-blockers, calcium channel blockers, diuretics, statins, fibrates, other lipid-lowering agents. CKD = Chronic kidney disease; ESRD = End-stage renal disease; CI = Confidence interval.

S. Figure 6. The association between onset of diabetes mellitus and kidney disease/mortality after censoring subjects with diagnosis of diabetes mellitus after baseline in the control group

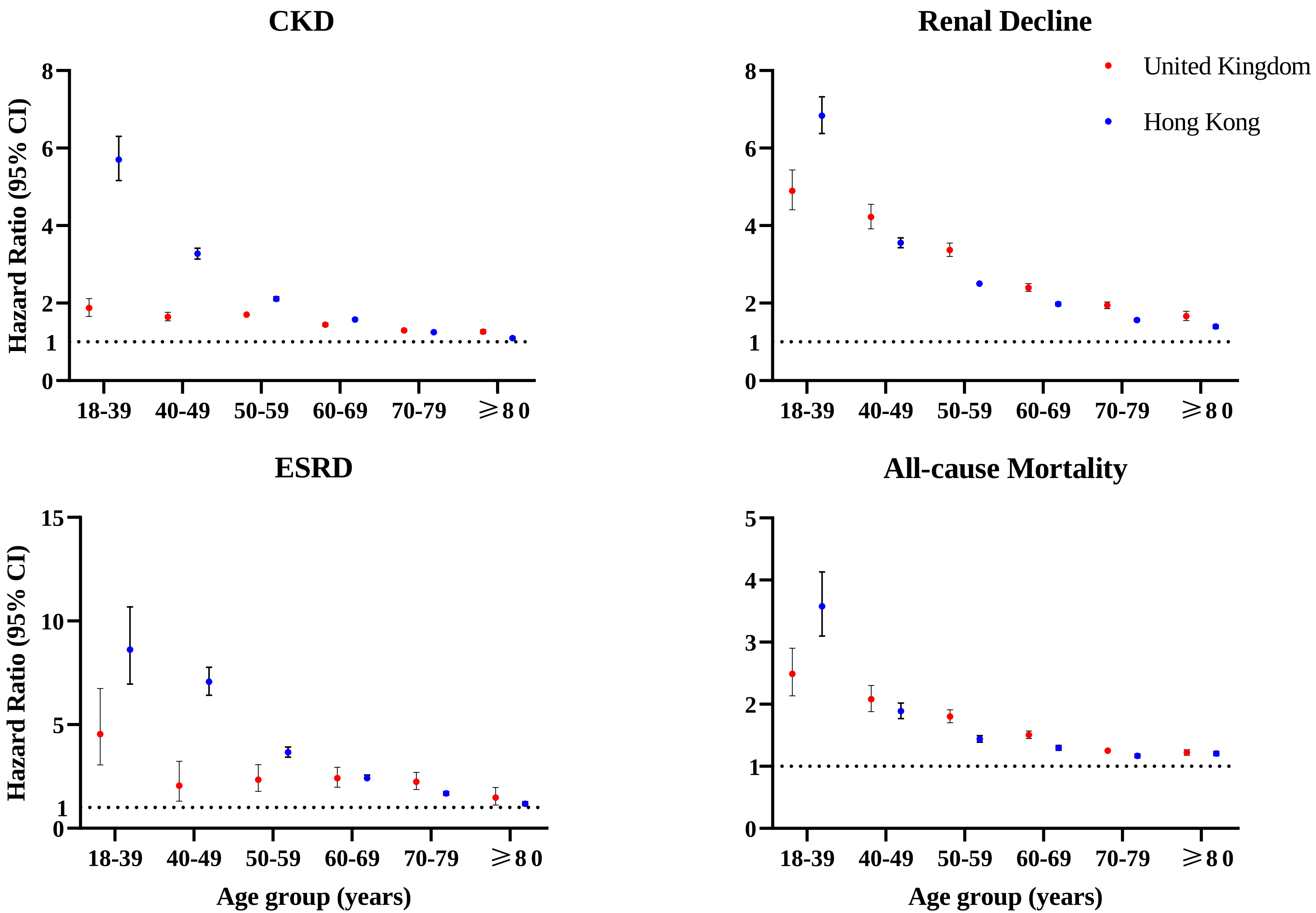

Hazard ratio with 95% confidence interval adjusted by age, sex, smoking status, comorbidities (i.e., obesity, atrial fibrillation, peripheral vascular disease, amputation, dementia, chronic lung disease, connective tissue disease, peptic ulcer disease, liver disease, cardiovascular disease, hemiplegia, leukemia, malignant lymphoma, cancer, hypertension, retinopathy, and hyperfiltration), and the use of renin-angiotensin system agents, beta-blockers, calcium channel blockers, diuretics, statins, fibrates, other lipid-lowering agents, and weighting.CKD = Chronic kidney disease; ESRD = End-stage renal disease; CI = Confidence interval.

S. Figure 7. The associations between onset of diabetes mellitus and kidney disease in different age groups adjusting mortality as the competing risk by Fine and Gray method.

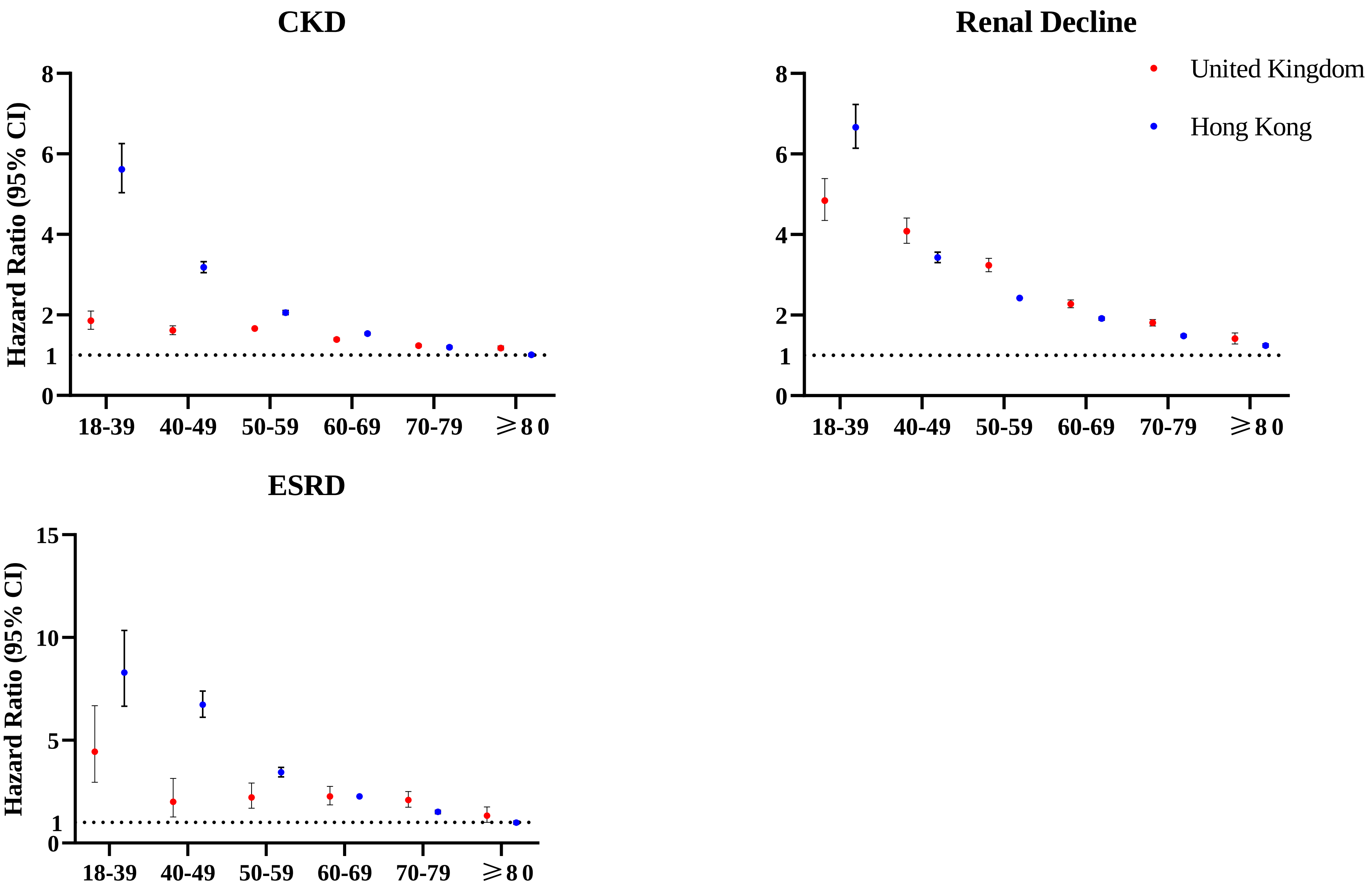

Hazard ratio with 95% confidence interval adjusted by age, sex, smoking status, comorbidities (i.e., obesity, atrial fibrillation, peripheral vascular disease, amputation, dementia, chronic lung disease, connective tissue disease, peptic ulcer disease, liver disease, cardiovascular disease, hemiplegia, leukemia, malignant lymphoma, cancer, hypertension, retinopathy, and hyperfiltration), and the use of renin-angiotensin system agents, beta-blockers, calcium channel blockers, diuretics, statins, fibrates, other lipid-lowering agents, and weighting. CKD = Chronic kidney disease; ESRD = End-stage renal disease; CI = Confidence interval.

S.Figure 8. Association between onset age of diabetes mellitus and the risk of kidney disease and mortality after treating age as a continuous variable using restricted cubic splines

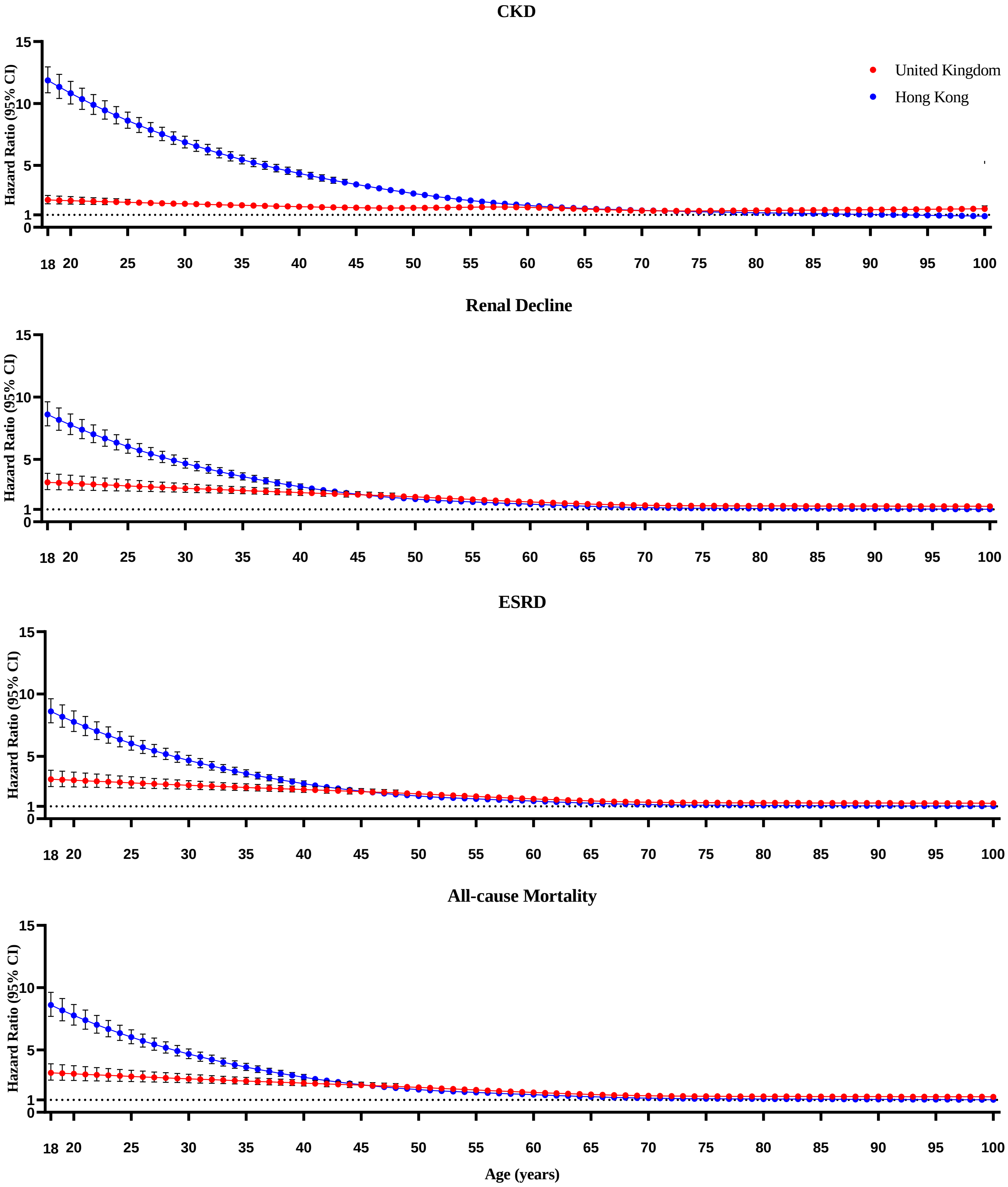

Hazard ratio with 95% confidence interval adjusted by age, sex, smoking status, comorbidities (i.e., obesity, atrial fibrillation, peripheral vascular disease, amputation, dementia, chronic lung disease, connective tissue disease, peptic ulcer disease, liver disease, cardiovascular disease, hemiplegia, leukemia, malignant lymphoma, cancer, hypertension, retinopathy, and hyperfiltration), and the use of renin-angiotensin system agents, beta-blockers, calcium channel blockers, diuretics, statins, fibrates, other lipid-lowering agents, and weighting. CKD = Chronic kidney disease; ESRD = End-stage renal disease; CI = Confidence interval.

S. Figure 9. Association between onset age of diabetes mellitus and the risk of renal decline, defined using a 40% cut-off value

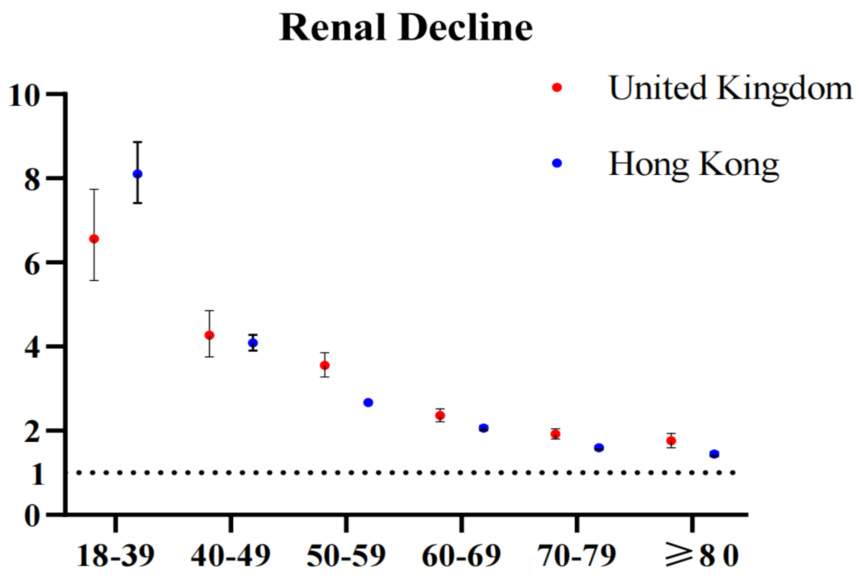

Hazard ratio with 95% confidence interval adjusted by age, sex, smoking status, comorbidities (i.e., obesity, atrial fibrillation, peripheral vascular disease, amputation, dementia, chronic lung disease, connective tissue disease, peptic ulcer disease, liver disease, cardiovascular disease, hemiplegia, leukemia, malignant lymphoma, cancer, hypertension, retinopathy, and hyperfiltration), and the use of renin-angiotensin system agents, beta-blockers, calcium channel blockers, diuretics, statins, fibrates, other lipid-lowering agents, and weighting. CKD = Chronic kidney disease; ESRD = End-stage renal disease; CI = Confidence interval.

S. Figure 10. Association between onset age of diabetes mellitus and the risk of kidney disease or mortality across different age groups among subjects with baseline eGFR.

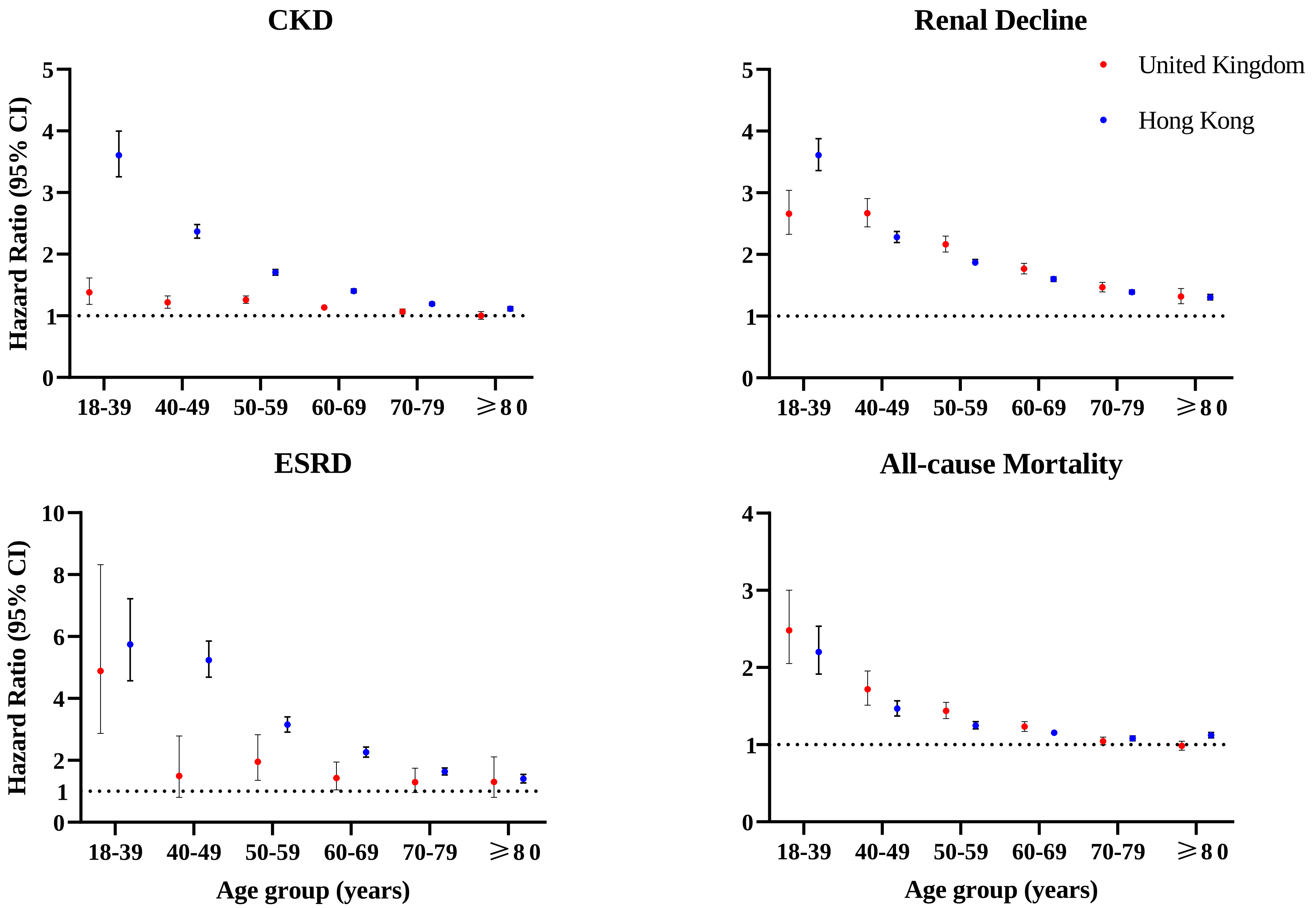

Hazard ratio with 95% confidence interval adjusted by age, sex, smoking status, comorbidities (i.e., obesity, atrial fibrillation, peripheral vascular disease, amputation, dementia, chronic lung disease, connective tissue disease, peptic ulcer disease, liver disease, cardiovascular disease, hemiplegia, leukemia, malignant lymphoma, cancer, hypertension, retinopathy, and hyperfiltration), and the use of renin-angiotensin system agents, beta-blockers, calcium channel blockers, diuretics, statins, fibrates, other lipid-lowering agents, and weighting. CKD = Chronic kidney disease; ESRD = End-stage renal disease; CI = Confidence interval.

S. Figure 11. Association between onset age of diabetes mellitus and the risk renal decine across different age groups by defining renal decline as a 30% decrease in eGFR within two years and the decline had to be observed in two consecutive eGFR measurements.

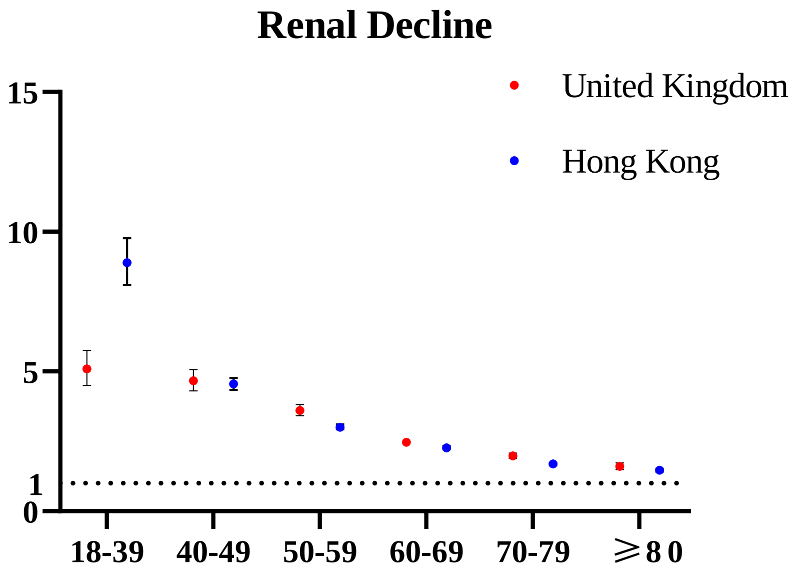

Hazard ratio with 95% confidence interval adjusted by age, sex, smoking status, comorbidities (i.e., obesity, atrial fibrillation, peripheral vascular disease, amputation, dementia, chronic lung disease, connective tissue disease, peptic ulcer disease, liver disease, cardiovascular disease, hemiplegia, leukemia, malignant lymphoma, cancer, hypertension, retinopathy, and hyperfiltration), and the use of renin-angiotensin system agents, beta-blockers, calcium channel blockers, diuretics, statins, fibrates, other lipid-lowering agents, and weighting. CKD = Chronic kidney disease; ESRD = End-stage renal disease; CI = Confidence interval.

Figure 12. The association between onset of diabetes mellitus and kidney disease/mortality in different age groups using Cox regression with time-dependent covariates.

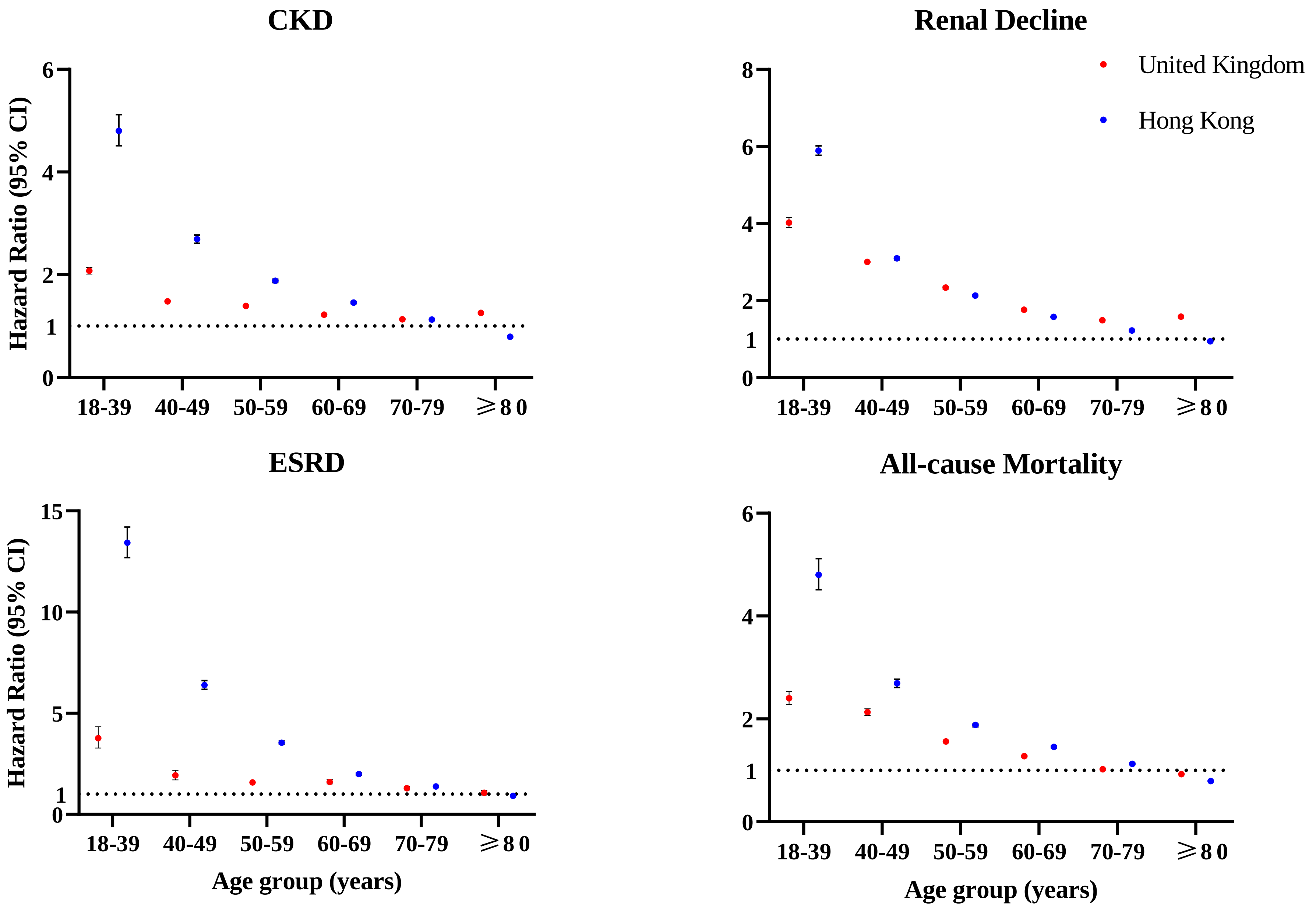

Hazard ratio with 95% confidence interval adjusted by age, sex, smoking status, comorbidities (i.e., obesity, atrial fibrillation, peripheral vascular disease, amputation, dementia, chronic lung disease, connective tissue disease, peptic ulcer disease, liver disease, cardiovascular disease, hemiplegia, leukemia, malignant lymphoma, cancer, hypertension, retinopathy, and hyperfiltration), and the use of renin-angiotensin system agents, beta-blockers, calcium channel blockers, diuretics, statins, fibrates, other lipid-lowering agents.CKD = Chronic kidney disease; ESRD = End-stage renal disease; CI = Confidence interval.

Figure 13. Association between onset age of diabetes mellitus and the risk of kidney disease or mortality across different age groups using Cox regression, among subjects with available blood pressure and low-density lipoprotein cholesterol (LDL-C) data at the index date.

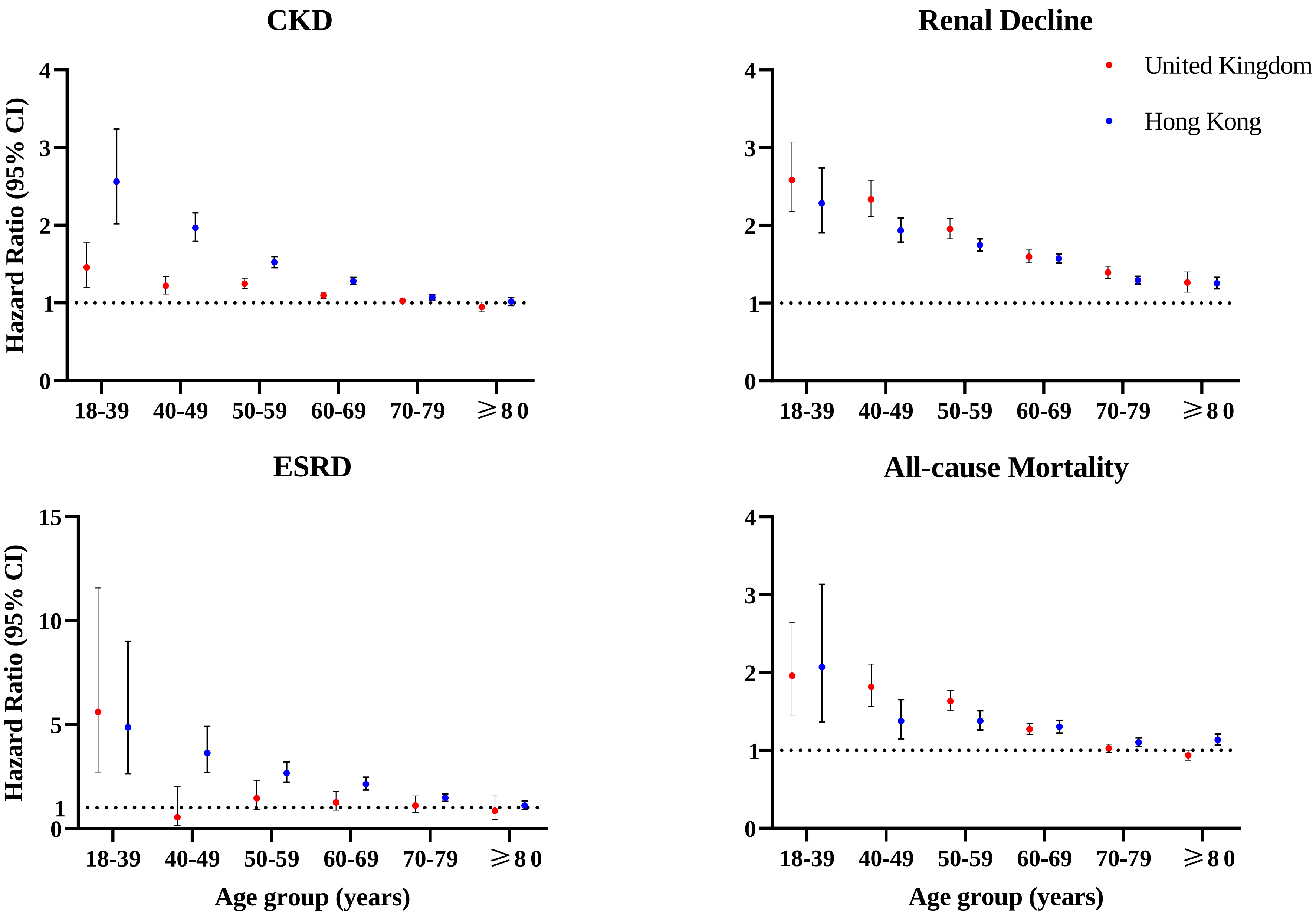

Hazard ratio with 95% confidence interval adjusted by age, sex, smoking status, comorbidities (i.e., obesity, atrial fibrillation, peripheral vascular disease, amputation, dementia, chronic lung disease, connective tissue disease, peptic ulcer disease, liver disease, cardiovascular disease, hemiplegia, leukemia, malignant lymphoma, cancer, hypertension, retinopathy, and hyperfiltration), and the use of renin-angiotensin system agents, beta-blockers, calcium channel blockers, diuretics, statins, fibrates, other lipid-lowering agents, systolic blood pressure, diastolic blood pressure, low-density lipoprotein cholesterol, and weighting. CKD = Chronic kidney disease; ESRD = End-stage renal disease; CI = Confidence interval.

Figure 14. The association between onset of diabetes mellitus and kidney disease/mortality in different age groups using Cox regression, in subjects without history of albuminuria (UACR ≥ 3 mg/mmol) at baseline.

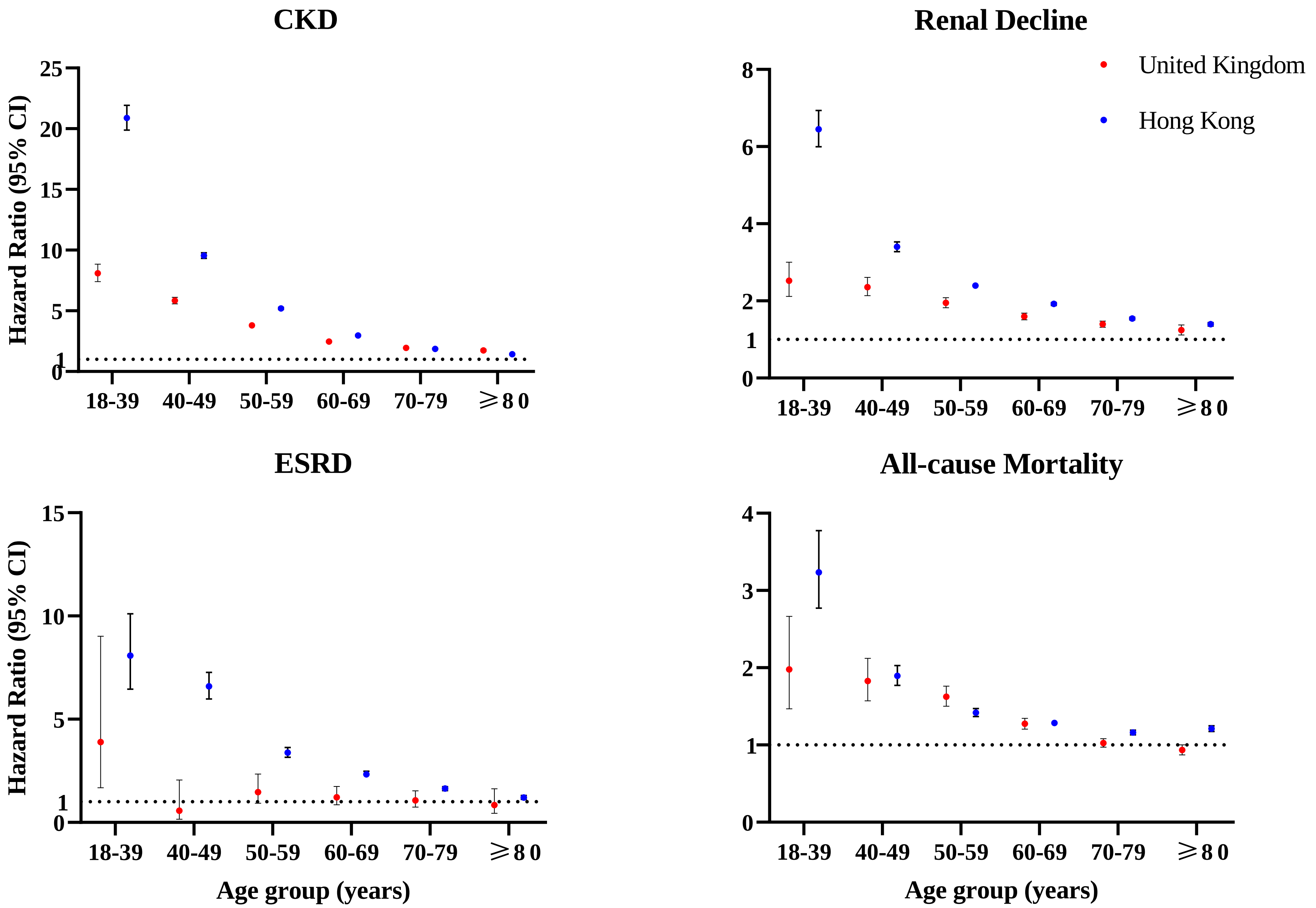

Hazard ratio with 95% confidence interval adjusted by age, sex, smoking status, comorbidities (i.e., obesity, atrial fibrillation, peripheral vascular disease, amputation, dementia, chronic lung disease, connective tissue disease, peptic ulcer disease, liver disease, cardiovascular disease, hemiplegia, leukemia, malignant lymphoma, cancer, hypertension, retinopathy, and hyperfiltration), and the use of renin-angiotensin system agents, beta-blockers, calcium channel blockers, diuretics, statins, fibrates, other lipid-lowering agents, and weighting. CKD = Chronic kidney disease; ESRD = End-stage renal disease; CI = Confidence interval. Chronic kidney disease (CKD) was defined based on diagnosis records, estimated glomerular filtration rate (eGFR) < 60 mL/min/1.73 m², or urine albumin-to-creatinine ratio (UACR) ≥ 3 mg/mmol.

Figure 15. Association between onset of diabetes mellitus and risk of kidney disease or mortality across different age groups, based on Cox regression after redefining hypertension

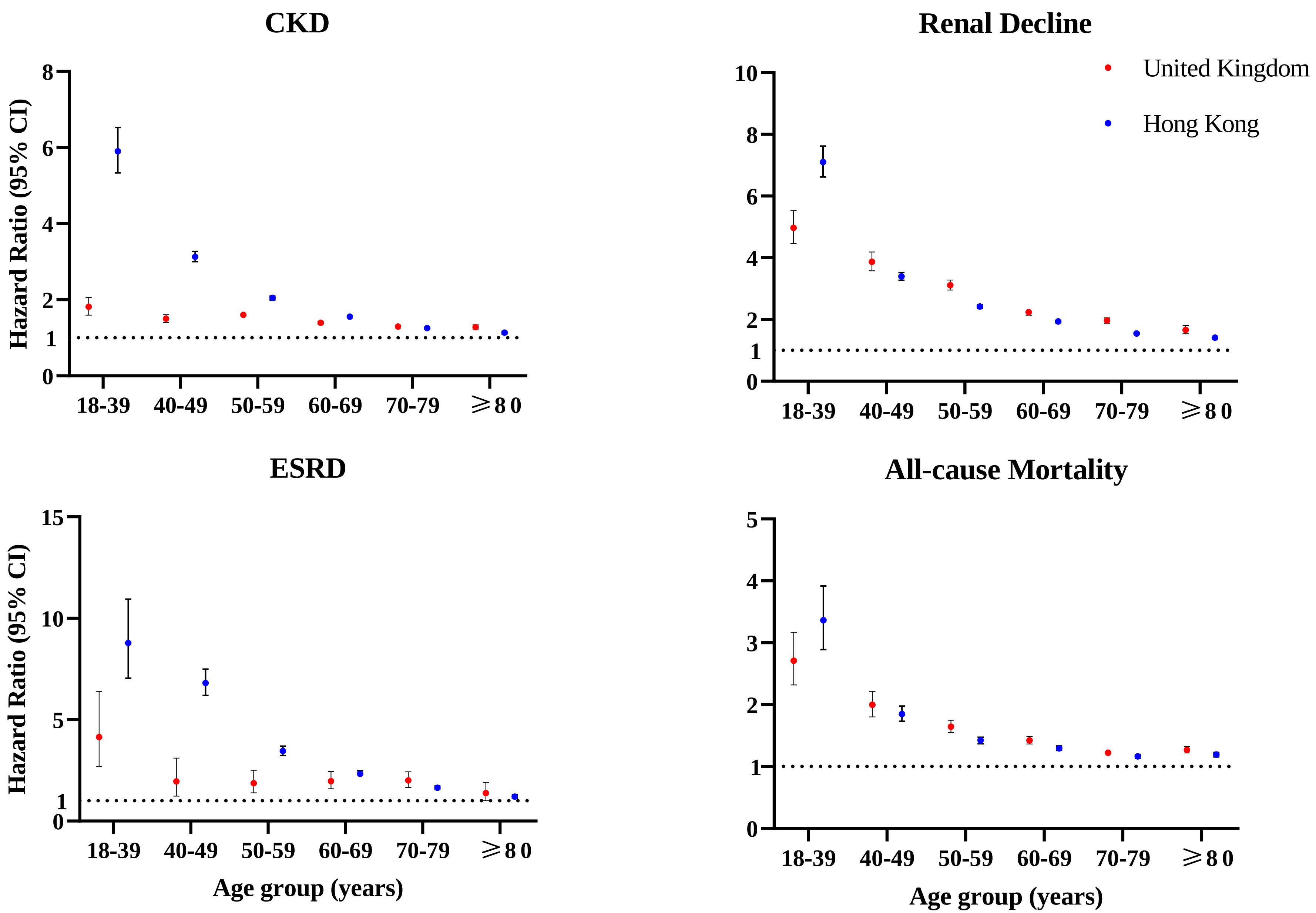

Hazard ratio with 95% confidence interval adjusted by age, gender, smoking status, obesity, hypertension, atrial fibrillation, peripheral vascular disease, amputation, dementia, lung disease, cumulative trauma disorder, peptic ulcer, liver, cardiovascular disease, hemiplegia, leukaemia, malignant lymphoma, cancer, retinopathy, hyperfiltration, the use of renin-angiotensin-system agents, beta blockers, calcium channel blockers, thiazide diuretics, other diuretics, statin, fibrate, other lipid lowering agents, and weighting. CKD = Chronic kidney disease; ESRD = End-stage renal disease; CI = Confidence interval.
